# Assembly-formed bioorthogonal chimeric artificial receptors enable high-contrast fluorescence imaging of tumors

**DOI:** 10.1126/sciadv.aed9403

**Published:** 2026-08-05

**Authors:** Jianli Zuo, Yuxi Tan, Zhaode Mu, Yuhan Dong, Yijie Wu, Yanzhi Li, Yonghua Yuan, Xiaobo Wang, Yongjun Dang, Lijuan Bai, Hongwen Liang

**Affiliations:** ^1^ Chongqing Research Center for Pharmaceutical Engineering, College of Pharmacy, Chongqing Medical University, Chongqing, China.; ^2^ Department of Urology of Jiangbei Campus, The First Affiliated Hospital of Army Medical University, Chongqing, China.; ^3^ Basic Medicine Research and Innovation Center for Novel Target and Therapeutic Intervention, Ministry of Education, College of Pharmacy, Chongqing Medical University, Chongqing, China.; ^4^ Department of Respiratory and Critical Care Medicine, Department of Tuberculosis, the First Affiliated Hospital of Chongqing Medical University, Chongqing, China.

## Abstract

Conventional receptor-targeted fluorescent probes have shown promise in tumor imaging, yet achieving a high tumor-to-normal (T/N) tissue ratio in vivo remains challenging due to limited biomarker density on tumor cell membranes. Here, we present an in situ assembly strategy of bioorthogonal-functionalized chimeric artificial receptors (BCARs) that locally constructs BCARs on tumor surfaces, which amplify fluorescence signals and enable high-contrast imaging. Rapid, selective membrane engineering under physiological conditions increases effective receptor density, enhancing fluorophore binding and tumor visualization. Mechanistic studies reveal that BCARs exhibit exceptional membrane retention and spatial precision, sustaining signal amplification in heterogeneous tumor microenvironments. In air-pouch and orthotopic bladder cancer models, BCARs notably improve the T/N imaging ratio and tumor boundary delineation. Translational validation with surgical specimens from 14 patients with bladder cancer confirms clinical feasibility. This work establishes a versatile platform for on-site receptor reprogramming and signal amplification, offering a powerful tool for high-contrast tumor margin detection.

## Teaser

Bioorthogonal assembly of chimeric receptors enables high-contrast imaging and real-time tumor margin detection in bladder cancer.

## INTRODUCTION

Bladder cancer is the most prevalent malignancy of the urinary tract and exhibits a high recurrence rate (*1*). Transurethral resection of bladder tumors (TURBT) remains the gold standard for the diagnosis, treatment, and staging of non–muscle-invasive bladder cancer (NMIBC) and clinicians often combine it with intravesical therapy to minimize tumor recurrence and progression (*2*, *3*). However, patients with positive surgical margins face a notably higher risk of recurrence as residual tumor cells may remain hidden within seemingly normal mucosa or erythematous areas that are challenging to distinguish from inflammation, contributing to recurrence rates of up to 78% (*4*–*6*). Therefore, there is a critical clinical need for real-time, accurate, and effective intraoperative assessment methods to identify positive surgical margins and reduce the likelihood of reoperation.

Fluorescence imaging represents a promising tumor detection method that researchers have applied in TURBT for margin assessment to help reduce the risk of recurrence (*7*, *8*). For example, clinicians have used the US Food and Drug Administration (FDA)–approved fluorescence agent Cysview for intraoperative margin evaluation in bladder cancer (*9*). However, because of its limited tumor-targeting ability, Cysview exhibits relatively low specificity, with an efficacy rate of only 61.2% (*10*). This highlights the urgent need for more precise diagnostic tools and strategies to improve the early detection of bladder cancer. Clinical and preclinical studies suggest that combining fluorescent probes with tumor-specific biomarkers can enhance tumor specificity and improve diagnostic accuracy (*11*–*16*). On the basis of this concept, Wang and our group developed an in vivo self-assembly strategy inspired by supramolecular chemistry (*17*–*20*). Researchers widely recognize this strategy as a promising approach to overcoming biological and physical barriers, increasing the local accumulation of fluorescent probes at target sites, and extending imaging signal retention (*21*). Notably, in situ assembled fibrous nanomaterials exhibited an assembly-induced retention (AIR) effect, which notably enhanced photoacoustic imaging signals (*22*).

Clinicians currently use intraoperative imaging systems in certain clinical settings, which have been shown to be easy to operate, cost-effective, capable of rapidly generating high-sensitivity images, and enhance real-time surgical navigation. Wang *et al.* developed a tumor-selective cascade activatable self-detained system targeting the overexpression of the X-linked inhibitor of apoptosis protein (XIAP) in tumor cells, which successfully applied to imaging guided bladder surgery in patients (*23*, *24*). Notably, the AIR effect notably enhanced the intracellular accumulation, retention, and photostability of fluorescent molecules in tumor cells, leading to distinct clinical advantages. However, they still face three notable challenges that require urgent attention: (i) The recognition efficiency of fluorescent probes remains limited by their reliance on a restricted number of biomarkers (∼10^6^ per cell) expressed on tumor cell surfaces, leading to suboptimal tumor cell targeting (*25*); (ii) relatively large antibodies or nanoparticles may hinder penetration into tumor tissue, particularly in noninvasive imaging requiring transurethral access to the bladder (*26*); and (iii) large fluorophores may reduce incorporation efficiency and cause photobleaching during surgery, affecting imaging performance (*27*).

To address these challenges, we developed a peptide recognized enhanced targeted imaging platform (PRE-targeted platform) that leverages the advantages of in vivo self-assembly and bioorthogonal coupling strategies (*28*–*32*). This PRE-targeted platform constructs bioorthogonal-functionalized chimeric artificial receptors (BCARs) through in situ self-assembly on the surface of tumor cells, thereby enhancing the number of bioorthogonal handles and improving the accuracy of fluorescence imaging agent labeling. Unlike the receptor-targeted strategy that relies on noncovalent interactions based on ligand-receptor recognition (*33*), the PRE-targeted platform aims to selectively and rapidly install high-density artificial receptors on the tumor cell membrane in a highly dynamic and complex in vivo environment, thereby reducing cellular interference caused by ligand-receptor recognition (*34*). The PRE-targeted platform enables two-step enrichment of reporters. First, peptides carrying a bioorthogonal handle, dibenzocyclooctyne (DBCO) are brought closer to the tumor cell membrane via receptors on the cell membrane. Then, enzymatic activity selective triggers in situ self-assembly, leading to efficient embedding on the cell membrane to form BCARs. Second, the azide-labeled fluorescent dye (azide-Cy5) binds to the BCAR through a bioorthogonal reaction, stably and chemically attaching fluorescent probe to the tumor cell membrane for efficient tumor imaging (fig. S1). As a proof of concept, our results demonstrated the efficacy of the PRE-targeted platform in enhancing the accumulation of azide-Cy5 specifically within tumor lesions using the mouse air-pouch bladder cancer (APBC) model, mouse orthotopic model, and ex vivo human bladder tissue, thereby notably improving the tumor-to-normal (T/N) ratio in imaging. Meanwhile, we substantially improved the photostability of azide-Cy5 by incorporating it into the nanomatrix, thereby preventing quenching during the bladder tumor imaging process. This advancement holds great promise for enhancing the diagnostic accuracy of bladder cancer. Moving forward, our aim is to further refine and translate this technology for clinical applications, potentially revolutionizing bladder cancer diagnosis and treatment monitoring.

## RESULTS

### CD44v6 is prominent biomarker in bladder cancer

CD44 is a transmembrane glycoprotein that plays a crucial role in tumor initiation, progression, invasion, metastasis, and drug resistance (*35*). On the basis of RNA sequencing (RNA-seq) data from over 1000 cell lines in the Cancer Cell Line Encyclopedia (CCLE) database, we systematically evaluated the expression patterns of CD44 across various cancers (fig. S2). These results revealed that CD44 is highly expressed in several tumor types, including gliomas, lung squamous cell carcinoma, and bladder cancer. Thus, we speculated that CD44 holds potential as a tumor-specific biomarker for bladder cancer.

**Fig. 1.**
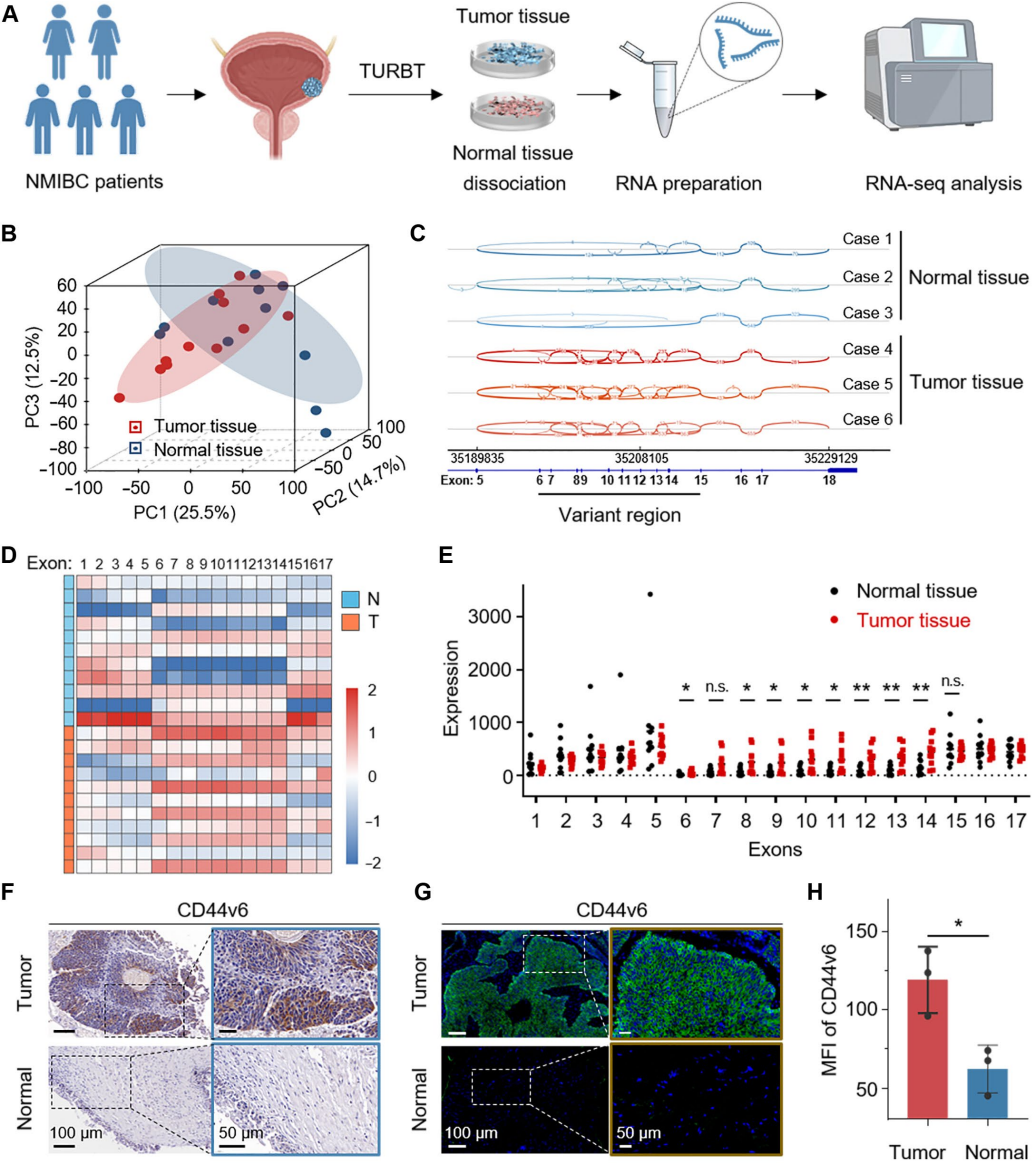
CD44v6 is highly expressed in human NMIBC. (**A**) Schematic of RNA-seq analysis in patients with NMIBC. (**B**) PCA using the DEGs identified in tumor tissue (*n* = 11) and normal tissue (*n* = 11) from patients with bladder cancer. (**C**) Representative sashimi image of CD44 in tumors tissue and normal tissue. (**D**) Heatmaps from RNA-seq analysis of all CD44 exons in tumor tissue (*n* = 11) and normal tissue (*n* = 11). (**E**) mRNA expression level of each CD44 exon in clinical tissue samples. The horizontal bars represent the mean. (**F**) IHC analysis with anti-CD44v6 antibody in tumor and normal tissues. *n* = 3. Original magnification: 30× (left) and 60× (right). (**G**) IF staining of tumor and normal tissues. *n* = 3. (**H**) Quantitative MFI of CD44v6. Data (*n* = 3) are presented as means ± SD. Statistical differences were analyzed using the two-tailed unpaired Student’s *t* test. n.s. represents no statistical difference and *P < 0.05.

To identify gene signatures predictive of clinical outcome, we collected 11 human bladder cancer tissues and 11 normal bladder tissues at the Department of Urology of Jiangbei Campus of the First Affiliated Hospital of Army Medical University performed eukaryotic transcriptome analysis (Fig. 1A). The three-dimensional (3D) principal components analysis (PCA) revealed the biological differences between the groups, with the first three principal components collectively explaining 52.7% of the total variance (PC1 25.5%, PC2 14.7%, and PC3 12.5%) (Fig. 1B). The tumor and normal tissues separated along the PC1 axis, indicating global differences in gene expression between the two groups. On the basis of stringent selection criteria (|log_2_FoldChange| > 1, *P* value < 0.05), we identified a total of 1729 differentially expressed genes (DEGs), among which 838 genes were notably up-regulated and 891 genes were notably down-regulated. We further validated the distribution characteristics of these DEGs through hierarchical clustering heatmaps and volcano plots (figs. S3 and S4).

Recent studies demonstrate that alternative splicing (AS) is a key mechanism in regulating cancer biology, with specific splice variants potentially driving oncogenesis and serving as therapeutic targets (*36*). Although studies have shown that the expression profiles of CD44 variant isoforms differ among various cancer types, researchers still lack a comprehensive quantitative characterization of these splice variants in bladder cancer (*37*). Therefore, we further analyzed CD44 splice variants using RNA-seq. Sashimi plot analysis revealed notable differences in splicing patterns between normal and bladder cancer tissues (Fig. 1C). Among the variable splice exons of CD44 (exons 6 to 15), the splicing frequencies of exons 6 (CD44v1), 8 (CD44v3), 9 (CD44v4), 10 (CD44v5), 11 (CD44v6), 12 (CD44v7), 13 (CD44v8), and 14 (CD44v9) increased notably in bladder cancer tissues compared to normal tissues (Fig. 1, D and E), indicating a distinct alteration in their expression patterns. These differences suggest that these specific exons may play a unique role in the development and progression of bladder cancer (*38*).

Furthermore, Gene Ontology (GO) and Kyoto Encyclopedia of Genes and Genomes (KEGG) enrichment analyses showed notable activation of pathways related to the extracellular matrix (ECM), cell-cell adhesion, focal adhesion, ECM-receptor interaction, proteoglycans in cancer, and cell adhesion molecules, suggesting a potential role in tumor progression and microenvironment remodeling (fig. S5, A to D). GO enrichment analysis showed that DEGs primarily enriched in ECM-related pathways, including collagen matrix, ECM-receptor interaction, ECM remodeling, and ECM structural components. The ECM signaling pathway not only participates in cell cycle regulation and proliferation but also plays a crucial role in tumor metastasis (*39*). Among these, CD44v6 acts as a key ECM receptor that binds to hyaluronic acid (HA), activates ERM/VEGFR and EGFR/LARG complexes, thereby promoting cytoskeletal remodeling and angiogenesis. CD44v6 interacts with the c-Met/HGF complex to activate the Ras/MAPK signaling pathway and further enhances the invasive capacity of bladder cancer cells (*40*). KEGG enrichment analysis further showed that DEGs were notably enriched in cell migration and proliferation-related pathways, such as focal adhesion, ECM-receptor interaction, proteoglycans in cancer, and cell adhesion molecules pathway (fig. S5C). Focal adhesions, as key sites of interaction between cells and the ECM, play a crucial role in this process. ECM molecules bind to the CD44 receptor, activating focal adhesion signaling and regulating the cytoskeleton to enhance the migratory capacity of tumor cells. This subsequently triggers a cascade of signaling events that activate the PI3K-Akt pathway, promoting cell proliferation and inhibiting apoptosis and thereby driving tumor progression (*41*). According to previous studies, CD44v6 can facilitate activation of both the Ras/MAPK pathway and the PI3K-Akt signaling pathway by acting as a co-receptor for HGF/c-MET. In addition, interaction of CD44v6 with HA can further contribute to activation of the PI3K-Akt signaling pathway, thereby promoting tumor cell proliferation and metastasis (*42*). Last, we performed immunohistochemistry (IHC) and immunofluorescence (IF) experiments to validate the expression of CD44v6 in human bladder cancer tissues (Fig. 1, F to H). These results showed that CD44v6 expression was nearly undetectable in normal tissues, whereas in bladder cancer tissues, its immunostaining intensity increased notably. Overall, these results highlight the critical role of CD44v6 in bladder cancer initiation and progression and suggest that CD44v6 may serve as a potential molecular biomarker for bladder cancer, providing a theoretical basis for clinical diagnosis.

### Design of DBCO-TPP and characterization of its self-assembly behavior in solution

**Fig. 2.**
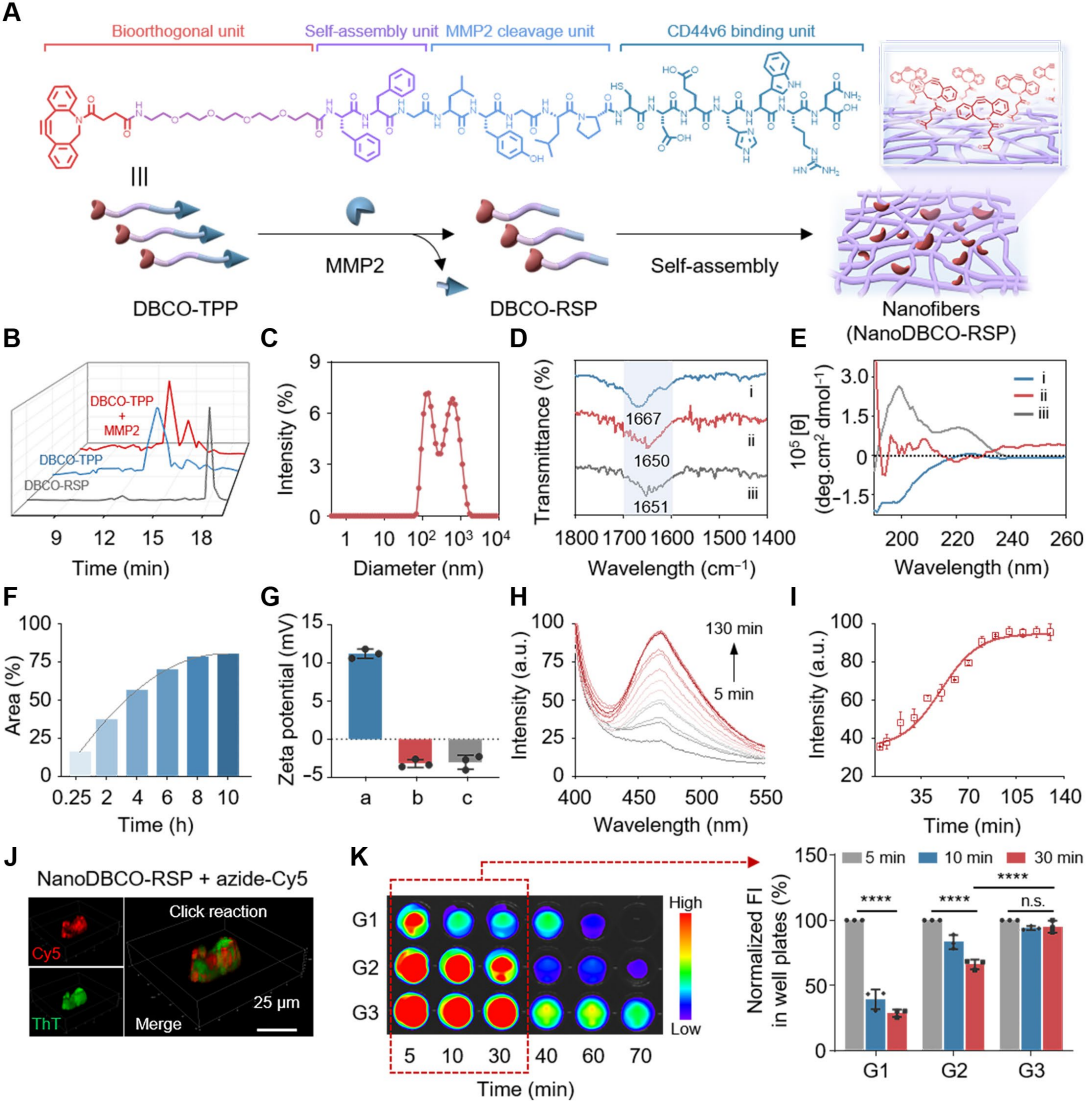
Characterization of the self-assembly behavior and photostability enhancement of fluorophores. (**A**) Schematic diagram of the MMP2 enzyme cleavage and self-assembly into nanofibers. (**B**) HPLC analysis of DBCO-TPP, DBCO-RSP, and DBCO-TPP + MMP2. (**C**) DLS of DBCO-TPP incubated with MMP2. FTIR spectra (**D**) and CD (**E**) of DBCO-TPP (50 μM), DBCO-TPP (50 μM) + MMP2 (2 μg ml^−1^), and DBCO-RSP (50 μM). i: DBCO-TPP; ii: DBCO-TPP + MMP2; iii: DBCO-RSP. (**F**) High-content screening (HCS) quantitative analysis of DBCO-TPP (50 μM) + MMP2 (5 μg ml^−1^) incubated with ThT (30 μM) for different times. h, hours. (**G**) Zeta potential of DBCO-TPP, DBCO-TPP + MMP2, and DBCO-RSP. Fluorescence spectra (**H**) and kinetic curve (**I**) of the click reactions of DBCO-RSP (50 μM) and coumarin (50 μM) at different times (5, 10, 20, 30, 40, 50, 60, 70, 80, 90, 100, 110, 120, and 130 min). a.u., arbitrary units. (**J**) CLSM images of DBCO-RSP (50 μM) incubated with ThT (30 μM) and azide-Cy5 (60 μM). (**K**) Photostability investigation of each group under laser irradiation (660 nm, 0.165 W cm^−2^) with different times. G1: PBS + azide-Cy5; G2: DBCO-TPP + azide-Cy5; G3: DBCO-TPP + MMP2 + azide-Cy5. Data (*n* = 3) are presented as means ± SD. Statistical differences were analyzed using the two-way ANOVA method. n.s. represents no statistical difference and ****P < 0.0001.

The PRE-targeted platform consists of DBCO-covalent modification of CD44v6 targeting precursor peptide (DBCO-TPP) and azide-Cy5. We synthesized DBCO-TPP [(DBCO)-PEG_4_-FFGLY↓GLPCDEHWRN] and the residue segment peptide [DBCO-RSP, (DBCO)-PEG_4_-FFGLY] after matrix metalloproteinase 2 (MMP2) enzyme cleavage of DBCO-TPP (figs. S6 and S7). As shown in Fig. 2A, MMP2-mediated enzymolysis of DBCO-TPP generated DBCO-RSP, which subsequently self-assembled into nanofibers. To verify the enzymolysis process, we used high-performance liquid chromatography (HPLC) analysis, which revealed the emergence of a new peak corresponding to DBCO-RSP (*T*_R_ = 18.1 min) and a concomitant decrease in the DBCO-TPP peak (*T*_R_ = 13.7 min) upon incubation with recombinant MMP2 in assay buffer (Fig. 2B). We further confirmed the formation of the conjugated product by HPLC and mass spectrometry (MS), with MS validating the expected molecular weight and structural identity, thereby demonstrating successful MMP2 cleavage and subsequent click conjugation to generate DBCO-RSP (fig. S8).

Subsequently, after enzymolysis of DBCO-TPP, the residues self-assembled into nanofibers (nanoDBCO-RSP). First, we measured the critical aggregation concentration (CAC) of DBCO-TPP before and after incubation with MMP2 using a fluorescent pyrene probe method (*43*). The CAC of DBCO-TPP exceeded 100 μM and then decreased to 23.7 μM after MMP2 incubation (fig. S9). Scanning electron microscopy (SEM) and transmission electron microscopy (TEM) analyses demonstrated the morphological transformation of DBCO-TPP from monomers to nanofibers after MMP2 incubation (fig. S10). The dynamic light scattering (DLS) measurements at different time points show that both the average hydrodynamic diameter and the polydispersity index (PDI) change over time, reflecting the assembly process of the system from monomers to nanofibers (Fig. 2C and figs. S11 and S12). By monitoring the time-dependent changes in 8-anilino-1-naphthalenesulfonic acid (ANS) fluorescence intensity (FI), we obtained a kinetic profile of β sheet conformational evolution and determined its half-life (*t*_1/2_ ≈ 1 hour) (fig. S13). We attribute the self-assembly mechanism of DBCO-TPP to disruption of the hydrophilic-hydrophobic balance caused by the departure of hydrophilic amino acids, which drives β sheet formation through intermolecular hydrogen bonding of Phe-Phe residues (*29*). Fourier transform infrared (FTIR) showed a shift of the amide I band from 1667 to 1650 cm^−1^ in the FTIR spectra after incubating DBCO-TPP with MMP2, almost consistent with the peak observed at 1651 cm^−1^ for DBCO-RSP (Fig. 2D) (*44*). In the circular dichroism (CD) spectrum, the DBCO-TPP + MMP2 exhibits a positive signal around 200 nm, suggesting the presence of regular π-π stacking interactions among aromatic residues (Phe-Phe). This signal further supports the formation of an ordered spatial arrangement of aromatic groups within nanofibers (Fig. 2E). We then used thioflavin T (ThT) as an amyloid sensor dye that exhibits strong fluorescence upon binding to amyloid fibrils (Fig. 2J and fig. S14) (*45*). After 1 hour of MMP2 incubation, the FI increased 2.76-fold, demonstrating the presence of β sheet structures within the nanofibers (fig. S15). Moreover, we monitored the self-assembly kinetics of DBCO-TPP at 25°C using the ThT assay. Figure 2F shows rapid initial growth followed by a slower phase, likely due to autocatalytic morphological transformation (fig. S16). We further measured the zeta potential of DBCO-TPP before and after MMP2 incubation. As shown in Fig. 2G, the surface charge shifted from +11.20 ± 0.60 mV to −3.21 ± 0.52 mV, showing no notable difference compared to DBCO-RSP (*P* = 0.92). Because positively charged molecules favor penetration through the bladder epithelium barrier, we anticipated that DBCO-TPP could enhance penetration into bladder cancer (*46*, *47*).

As illustrated the procedure for PRE-targeted platform imaging in vivo via successive self-assembly of DBCO-TPP (first step) and the bioorthogonal reaction with DBCO (second step) (fig. S1). We evaluated the grafting of Cy5 onto nanoDBCO-RSP using a model reaction: Nonfluorescent coumarin (3-azido-7-hydroxycoumarin) reacted with nanoDBCO-RSP in H_2_O/dimethyl sulfoxide (DMSO) (95:5) at 25°C to form a fluorescent product (fig. S17, A and B). Figure 2 (H and I) shows a notable increase in FI within 60 min, attributed to the reaction between the azido group in 3-azido-7-hydroxycoumarin and the DBCO group in nanoDBCO-RSP, facilitated by internal charge transfer (ICT) (*48*). On the basis of the ratios of nanoDBCO-RSP to 3-azido-7-hydroxycoumarin ranging from 1:0.002 to 1:1.4, we found that the optimal DBCO-RSP/azide-Cy5 ratio for maximum FI is 1:1 (fig. S17, C and D).

Photostability is a critical factor in determining the clinical applicability of fluorescent molecules, and the PRE-targeted platform effectively enhances their photostability. In clinical practice, TURBT typically takes between 20 and 30 min to complete. Therefore, we principally compared photobleaching resistance within the first 30 min. First, we performed control experiments using azide-Cy5 alone and time-resolved fluorescence measurements to exclude background signals and quenching effects from unreacted probe (fig. S18). Then, we separately incubated three groups in phosphate-buffered saline (PBS) (G1), DBCO-TPP (G2), and DBCO-TPP + MMP2 for 2 hours (G3). We treated each group with azide-Cy5 and irradiated them with a 660-nm laser (0.165 W cm^−2^) for 5, 10, 30, 40, 60, and 70 min in 96-well plates. As shown in Fig. 2K, within 30 min of laser irradiation, the FI of G1 notably decreased and quantitative normalization of FI indicated that the FI of the azide-Cy5 decreased to 29%. In G2, there was a slight decrease, with quantitative normalization results showing a decrease of 34% (figs. S19 and S20). We optimized the molar ratio of DBCO-TPP to azide-Cy5 and selected 1:1 as the optimal condition based on stable fluorescence response and reaction efficiency across tested ratios (1:0.25 to 1:4) (fig. S21). Light dose-dependent photobleaching analysis revealed that the fluorescence of G1 and G2 gradually decreased with increasing light dose, whereas G3 remained highly stable (fig. S22). This decrease could be attributed to the grafting of azide-Cy5 to DBCO-TPP monomers, which, to some extent, inhibits the nonradiative internal conversion of Cy5. In contrast, in G3 that formed nanoDBCO-RSP, which could be grafting by azide-Cy5, the FI remained almost unchanged. The above results further demonstrate that the β sheet structure of nanoDBCO-RSP can improve the photostability of fluorescent molecules (*27*).

### DBCO-TPP formed nanofibers on CD44v6-overexpressing cells in vitro

**Fig. 3.**
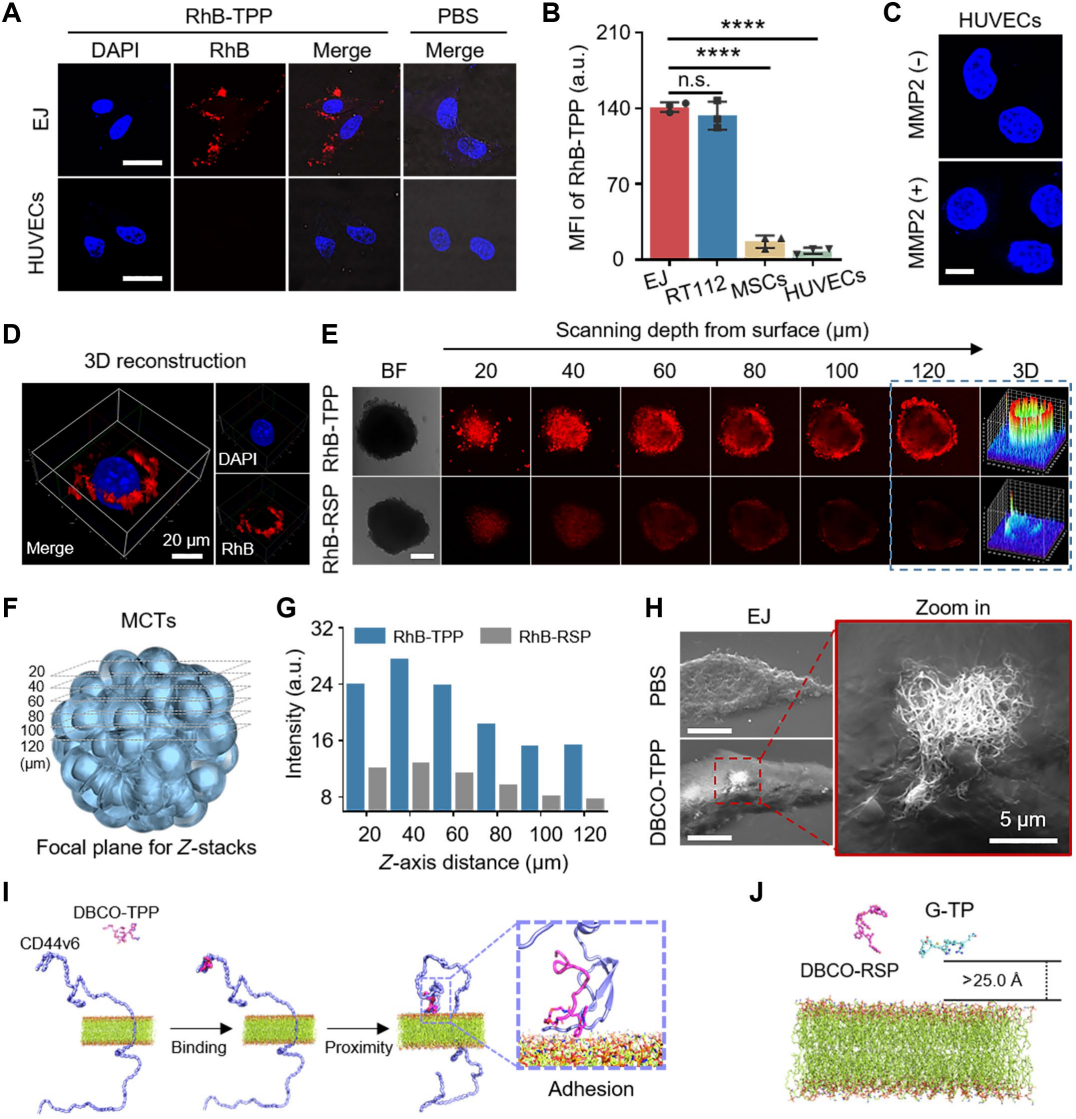
In situ self-assembly of DBCO-TPP in cell membranes and permeability in vitro. (**A**) CLSM images and (**B**) FI of EJ and HUVECs incubated with RhB-TPP (50 μM) for 2 hours. Scale bars, 20 μm. DAPI, 4′,6-diamidino-2-phenylindole. (**C**) CLSM images of HUVECs incubated with RhB-TPP in the presence and absence of MMP2. (**D**) The 3D construction diagram of the incubation with RhB-TPP in RT112 cells for 2 hours. (**E**) CLSM images of EJ MCTs (20 to 120 μm) taken at 20-μm intervals after incubation with RhB-TPP (50 μM) and RhB-RSP (50 μM) for 2 hours. Scale bar, 100 μm. (**F**) Schematic illustration of 3D CLSM imaging in tumor spheroid cross sections. (**G**) FI of RhB-TPP and RhB-RSP in the *z*-axis distance (20 to 120 μm). (**H**) SEM images of EJ cells incubated with PBS and DBCO-TPP for 12 hours. Scale bars, 20 μm. (**I**) Kinetic simulation and structural changes of the phospholipid membranes after binding of the CD44v6 protein to DBCO-TPP. Cyan: phospholipid membrane; blue: CD44v6 protein; magenta: DBCO-TPP. (**J**) Structural phase plots of the formation of DBCO-RSP and G-TP from DBCO-TPP after it is brought close to the phospholipid membranes and cleaved by MMP2. Data (*n* = 3) are expressed as means ± SD. Statistical differences were analyzed using the ANOVA method. n.s. represents no statistical difference and ****P < 0.0001.

Before evaluating in situ self-assembly of DBCO-TPP on tumor cells, we evaluated the biocompatibility of DBCO-TPP via cell viability assay. We found no obvious cytotoxicity (fig. S23). We selected EJ and RT112 cells that highly express CD44v6 to explore the recognition specificity of DBCO-TPP. We incubated rhodamine B–labeled TPP (RhB-TPP) with EJ and RT112 cells for 2 hours and observed colocalization via confocal laser scanning microscopy (CLSM). Meanwhile, we selected human umbilical cord endothelial cells (HUVECs) and mesenchymal stem cells (MSCs), which express minimal CD44v6, as control groups. Consequently, we observed notable fluorescence on the membranes of EJ and RT112 cells, whereas we detected no fluorescence in HUVECs and MSCs (Fig. 3, A and B, and figs. S24 to S29). It is worth emphasizing that, even after adding MMP2 to HUVECs, we still observed no fluorescence signal, further confirming that CD44v6-targeted recognition is a critical prerequisite for self-assembly on the cell surface (Fig. 3C). The 3D CLSM images further validated that DBCO-TPP localized on the cell membrane surface (Fig. 3D and figs. S30 and S31). All these CLSM results indicate that DBCO-TPP exhibits high CD44v6-targeting specificity and that its self-assembly process depends on CD44v6 and MMP2 enzymatic activity.

Multicellular tumor spheroids (MCTs) simulate the complicated tumor microenvironment in vitro (*49*). Therefore, we established MCTs based on EJ cells to evaluate the penetration and retention performance of DBCO-TPP (fig. S32). To investigate whether the penetration performance of DBCO-TPP in CD44v6-positive MCTs depends on the CD44v6 targeting segment, we selected DBCO-RSP as a control group, which does not include the CD44v6 targeting segment. The fluorescence signals of RhB-TPP remained visible at depths of up to 120 μm, whereas those of RhB-RSP became barely detectable beyond 60 μm (Fig. 3, E to G, and fig. S33). We observed fluorescence spots, which likely correspond to self-assembled nanofibers formed after MMP2 cleavage of DBCO-TPP. Bio-TEM provided visual evidence of reticulated nanofiber structures in the intercellular space (fig. S34). Furthermore, we evaluated the retention performance of RhB-TPP in MCTs over time, and CLSM results showed that fluorescence signals remained nearly unchanged at 0.5, 2, 3, 5, and 7 hours (fig. S35). Collectively, these findings indicate that DBCO-TPP has excellent penetration capability and undergoes in situ self-assembly into nanofibers, enabling prolonged membrane retention via the AIR effect (*22*).

Subsequently, as depicted in Fig. 3H and fig. S36, SEM revealed that nanofibers visibly attached to the surfaces of DBCO-TPP–treated EJ and RT112 cells. On the contrary, we detected no nanofiber-like structures in the PBS group or in HUVECs and MDA-MB-231 cells, regardless of the presence of MMP2 in the culture medium. We investigated the process by which DBCO-TPP forms DBCO-RSP and adheres to the cell membrane using molecular dynamics (MD) simulations (*50*). The structure of the CD44v6 protein is highly dynamic. Upon binding to DBCO-TPP, CD44v6 pulls the DBCO-TPP from its free-floating position in the solvent, originally distant from the phospholipid membrane, and brings it closer to the membrane, partially adhering to it (Fig. 3I). This result suggests that DBCO-TPP specifically targets and binds to CD44v6 on the cell membrane, thereby reducing the distance between DBCO-TPP and the membrane. In conclusion, these results demonstrate that DBCO-TPP undergoes successful in situ self-assembly through a cascade process involving recognition and enzyme-triggered mechanisms and exhibits an assembly-enhanced proximity adhesion (AEPA) effect.

### Dynamic mechanism of the AEPA effect of DBCO-TPP

**Fig. 4.**
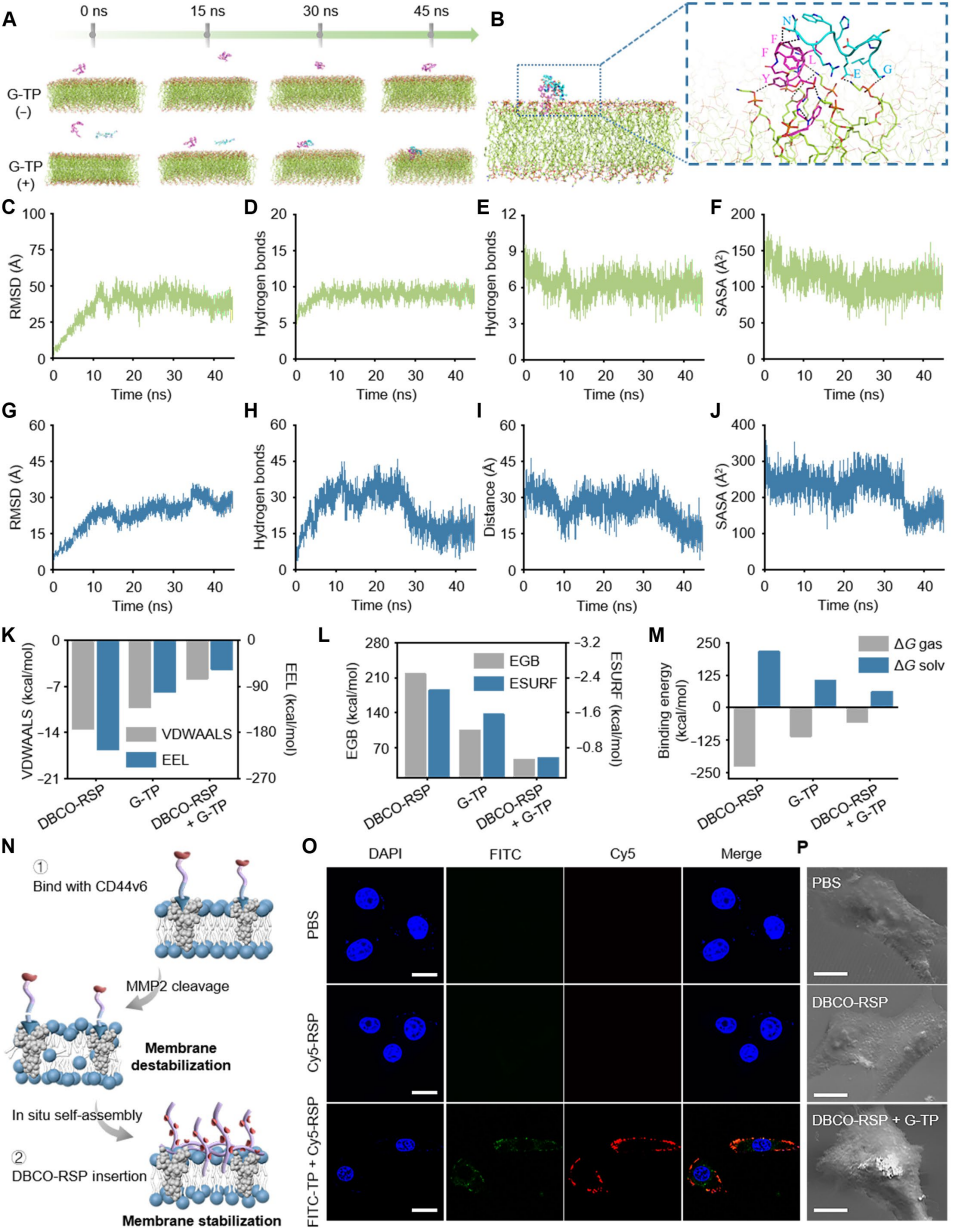
Mechanistic study and validation of the AEPA effect triggered by the in situ self-assembly of DBCO-TPP. (**A**) Dynamic conformational changes of DBCO-RSP and G-TP + DBCO-RSP at 0, 15, 30, and 45 ns. (**B**) Representative configurations of peptides and the bilayer at an equilibrium stage in MD simulations. (**C**) RMSD, (**D**) intramolecular hydrogen bonds, (**E**) DBCO-RSP water hydrogen bonding, and (**F**) SASA during the self-assembly of DBCO-RSP in water. In the presence of G-TP, (**G**) RMSD, (**H**) intramolecular hydrogen bonding, (**I**) distance to the phospholipid bilayer, and (**J**) SASA were analyzed following the interaction of DBCO-RSP with the cell membrane. (**K** to **M**) Binding energy between different peptides and phospholipid membranes, including van der Waals energy (VDWAALS), electrostatic energy (EEL), polar solvation energy (EGB), nonpolar solvation energy (ESURF), total gas-phase free energy (Δ*G* gas), and total solvation free energy (Δ*G* solv). (**N**) Schematic illustration of the AEPA process. (**O**) Representative CLSM image of EJ cells treated with PBS, FITC-TP, and Cy5-RSP + FITC-TP. Scale bars, 20 μm. (**P**) Representative SEM image of EJ cells treated with PBS, DBCO-RSP, and DBCO-RSP + G-TP. Scale bars, 20 μm.

To gain a deeper understanding dynamic mechanism of the AEPA effect, we constructed pure phospholipid membrane bilayers containing 320 lipid molecules and 160 POPE (palmitoyloleoylphosphatidylethanolamine) molecules using the membrane builder module of the CHARMM-GUI software and performed all-atom MD simulations, elucidating the molecular interactions between the nanofibers and the phospholipid membranes. Initially, after DBCO-TPP recognized the CD44v6 receptor, MMP2 cleaved it into DBCO-RSP and peptides containing TP (GLPCDEHWRN, G-HP) (fig. S37A). By default, DBCO-RSP and G-TP adopt a free state near the phospholipid membrane with a distance of less than 25 Å (Fig. 3J). Figure 4A shows snapshots at different time points, which indicate that DBCO-RSP undergoes structural rearrangement, migration, and gradual self-aggregation, eventually reaching a stable and nondissociative equilibrium state (fig. S37, B and E to G). Subsequently, we simulated the assembly process of DBCO-RSP in water. MD simulation results showed that DBCO-RSP self-assembled into a stable aggregate after 10 Å. The root mean square deviation (RMSD) of the system stabilized at 40 Å after 10 ns, with fluctuations within a range of 10 Å (Fig. 4C). The aggregate tended to stabilize in the solvent system, which was consistent with the results of the RMSF analysis (fig. S37C). The trend in the number of intermolecular hydrogen bonds between DBCO-RSP also increased initially and stabilized after 10 ns at ∼10, possibly due to the presence of multiple amide bonds in DBCO-RSP, which interact with other DBCO-RSP (Fig. 4D). Moreover, the number of hydrogen bonds between DBCO-RSP and water molecules gradually decreased, reduced the surface interactions with water molecules (Fig. 4E). In addition, the solvent-accessible surface area (SASA) of the whole system reached a stable level after 10 ns, indicated that the aggregate was unlikely to dissociate (Fig. 4F). These results suggest that DBCO-RSP rapidly forms stable aggregates in aqueous solution.

We further investigated the interaction between DBCO-RSP and the cell membrane in the presence of G-TP. As shown in Fig. 4A, snapshots taken at different time points indicate that in the presence of G-TP, the hydrophobic amino acids of G-TP can combine with DBCO-RSP to produce agglomeration effects, as well as the good binding effect between DBCO-RSP and the phospholipid membrane, enabling DBCO-RSP and G-TP to aggregate and adhere to the phospholipid membrane (Fig. 4B and fig. S37B). Time-dependent CLSM imaging of the NBD (NBD serves as an environment-sensitive fluorescent probe that exhibits enhanced fluorescence in hydrophobic or aggregated states)–labeled peptide (NBD-TPP) demonstrates pronounced membrane-associated assembly and punctate clustering in EJ and T24 cells, whereas TUBO and HUVECs show negligible fluorescence, providing an evidence that DBCO-TPP selectively assembles at the plasma membrane (figs. S38 and S39). Kinetic simulation results showed that the RMSD of this system stabilizes at 10 ns, exhibits a change at 35 ns, and then quickly enters a steady state, which was consistent with the results from the root-mean-square fluctuation (RMSF) analysis (Fig. 4G and fig. S37, D and H to J). The number of intermolecular hydrogen bonds in DBCO-RSP follows a similar trend as in aqueous solution before 35 ns but then decreases notably and stabilizes after 35 ns (Fig. 4H). We attribute this change to interactions between nanofibers and the cell membrane, where DBCO-RSP forms hydrogen bonds with the phospholipid bilayer, reducing intermolecular bonding. Figure 4I shows that the nanofiber front end adheres to the phospholipid layer, gradually approaching one side of the bilayer until the distance reaches ∼10 Å and remains stable. The SASA of the system decreases by ∼30% at 35 ns and quickly stabilizes, which indicates that insertion into the membrane enhances DBCO-RSP stability on the cell surface (Fig. 4J). At 45-ns equilibration, we calculated binding energies between peptides and phospholipid membranes (Fig. 4, K to M). Van der Waals, electrostatic, polar solvation, nonpolar solvation, and total binding free energies all indicate that DBCO-RSP interacts most strongly with the phospholipid membrane, whereas interaction with G-TP is the weakest (fig. S40). Compared with DBCO-RSP alone, G-TP + DBCO-RSP exhibits a larger contact area and lower binding energy and distance (fig. S41 and table S1). Thermodynamic and dynamic parameters further indicate that insertion of DBCO-RSP into the membrane promotes a more stable state. Collectively, MD simulations indicate that DBCO-RSP preferentially inserts into the membrane after G-TP binding to CD44v6 and forms stable nanofibers.

In support of these findings, we validated the significance of G-TP binding CD44v6 in promoting the insertion of DBCO-RSP into the cell membrane (Fig. 4N). We incubated fluorescein isothiocyanate (FITC)–labeled G-TP with EJ cells for 30 min and used untreated FITC-TP as control. We then added Cy5-labeled RSP and incubated for another 20 min. CLSM imaging showed that the FITC-TP–treated group exhibited green fluorescence on the cell surface, whereas the control group showed no signal (Fig. 4O). SEM confirmed these findings, showing nanofibers on G-TP–treated cells, whereas control cells showed none (Fig. 4P and fig. S42). These findings agree with MD predictions and confirm that G-TP binding to CD44v6 is essential for DBCO-RSP membrane insertion. Furthermore, to investigate the potential impact of membrane-anchored nanofibers on the biophysical properties of tumor cells, fluorescence recovery after photobleaching (FRAP) experiments were performed (fig. S43). The results showed that, compared with the control group, cells treated with CD44v6–G-TP exhibited an increased fluorescence recovery (85.66%), whereas an even higher recovery was observed in the DBCO-TPP group (99.31%). These findings indicate enhanced lateral mobility or exchange dynamics of membrane-associated components. We speculate that the insertion and assembly of nanofiber structures on the cell membrane may modulate lipid organization, thereby influencing membrane dynamics.

**Fig. 5.**
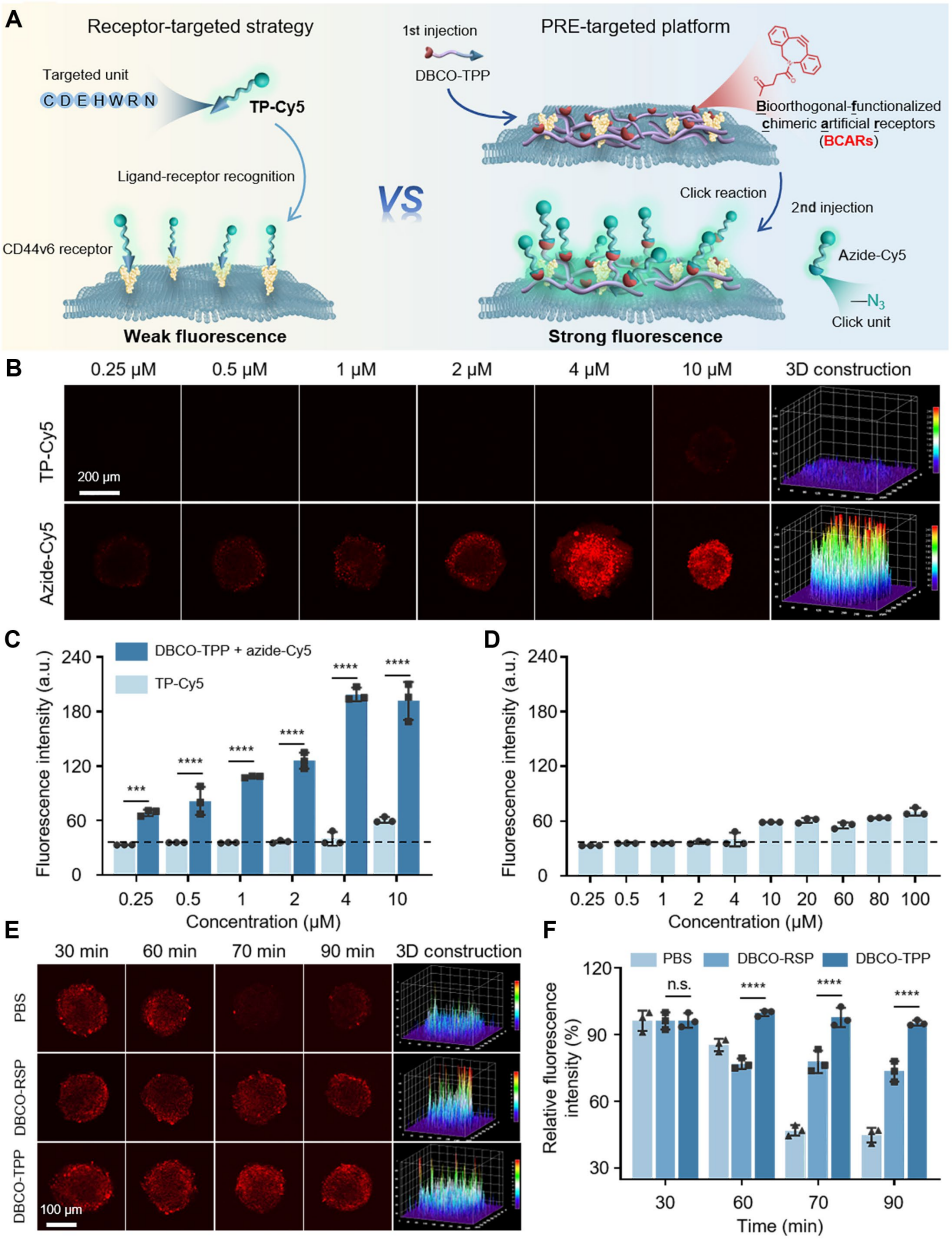
BCAR enhances fluorophore imaging performance. (**A**) Diagram illustrating the improved recognition efficiency of fluorescent molecules mediated by BCARs. (**B**) CLSM images and (**C**) FI quantitative analysis of EJ MCTs were pretreated with DBCO-TPP (50 μM) followed by the addition of azide-Cy5 (0.25 to 10 μM) for a further 30 min and TP-Cy5 (0.25 to 10 μM) as control group for 30 min. Scale bar, 200 μm. (**D**) FI quantitative analysis of TP-Cy5 in the concentration range from 0.25 to 100 μM. (**E**) CLSM images and (**F**) relative FI of DBCO-TPP (50 μM), DBCO-RSP (50 μM), and PBS coincubated with azide-Cy5 (2 μM) in EJ MCTs and irradiated under 660 nm for different times. Data (*n* = 3) were expressed as means ± SD, and statistical differences were analyzed by the two-way ANOVA method. n.s. represents no statistical difference, ***P < 0.001, and ****P < 0.0001.

To compare imaging efficiency between conventional receptor-targeted probes and PRE-targeted imaging, we synthesized TP-Cy5, which binds specifically to CD44v6 receptors (Fig. 5A) (*51*). We first preincubated EJ MCTs with DBCO-TPP for 0.5 hours, followed by coincubation with azide-Cy5. CLSM imaging showed that the FI increased with concentration (0.25 to 4 μM) and reached saturation, indicating efficient probe accumulation due to high-density BCAR formation. In contrast, TP-Cy5 labeling showed nearly constant fluorescence across 0.25 to 10 μM and no notable increase even at 100 μM, suggesting receptor saturation at low concentrations. At 4 μM, BCARs constructed with DBCO-TPP notably enhanced FI compared with native receptors, achieving up to a 5.0-fold increase (Fig. 5, B to D, and fig. S44). Moreover, the limit of detection of EJ cells labeled with DBCO-TPP/azide-Cy5 was determined to be 108 cells per well based on the minimum detectable fluorescence signal (fig. S45). We attribute this effect to limited native receptor expression on tumor cells, which restricts probe binding. These findings demonstrate that the PRE-targeted platform increases BCAR density and enhances fluorescent probe accumulation, leading to improved tumor imaging performance.

Then, we verified that the PRE-targeted platform enhances the photostability of fluorescent probes. We preincubated MCTs with PBS, DBCO-RSP, and DBCO-TPP for 0.5 hours and then incubated them with azide-Cy5 for another 30 min and irradiated them with a 660nm laser for 30, 60, 70, and 90 min to simulate fluorescence cystoscopy. As shown in Fig. 5E, CLSM imaging showed that after 30 min of irradiation, fluorescence intensities of PBS + azide-Cy5, DBCO-RSP + azide-Cy5, and DBCO-TPP + azide-Cy5 were nearly identical. However, with prolonged irradiation, the FI of DBCO-TPP + azide-Cy5 remained almost unchanged, whereas DBCO-RSP + azide-Cy5 decreased slightly, and PBS + azide-Cy5 decreased most notably (Fig. 5F). We speculate that, in the DBCO-TPP + azide-Cy5 group, DBCO-TPP undergoes in situ self-assembly and forms nanofibers within MCTs that are more ordered than preassembled DBCO-RSP structures in the DBCO-RSP + azide-Cy5 group. This highly ordered arrangement of grafted fluorescent molecules enhances molecular rigidity and planarity, thereby restricts nonradiative relaxation processes and reduces photobleaching (*52*).

### Air-pouch and orthotopic tumor imaging in mice via the PRE-targeted platform

**Fig. 6.**
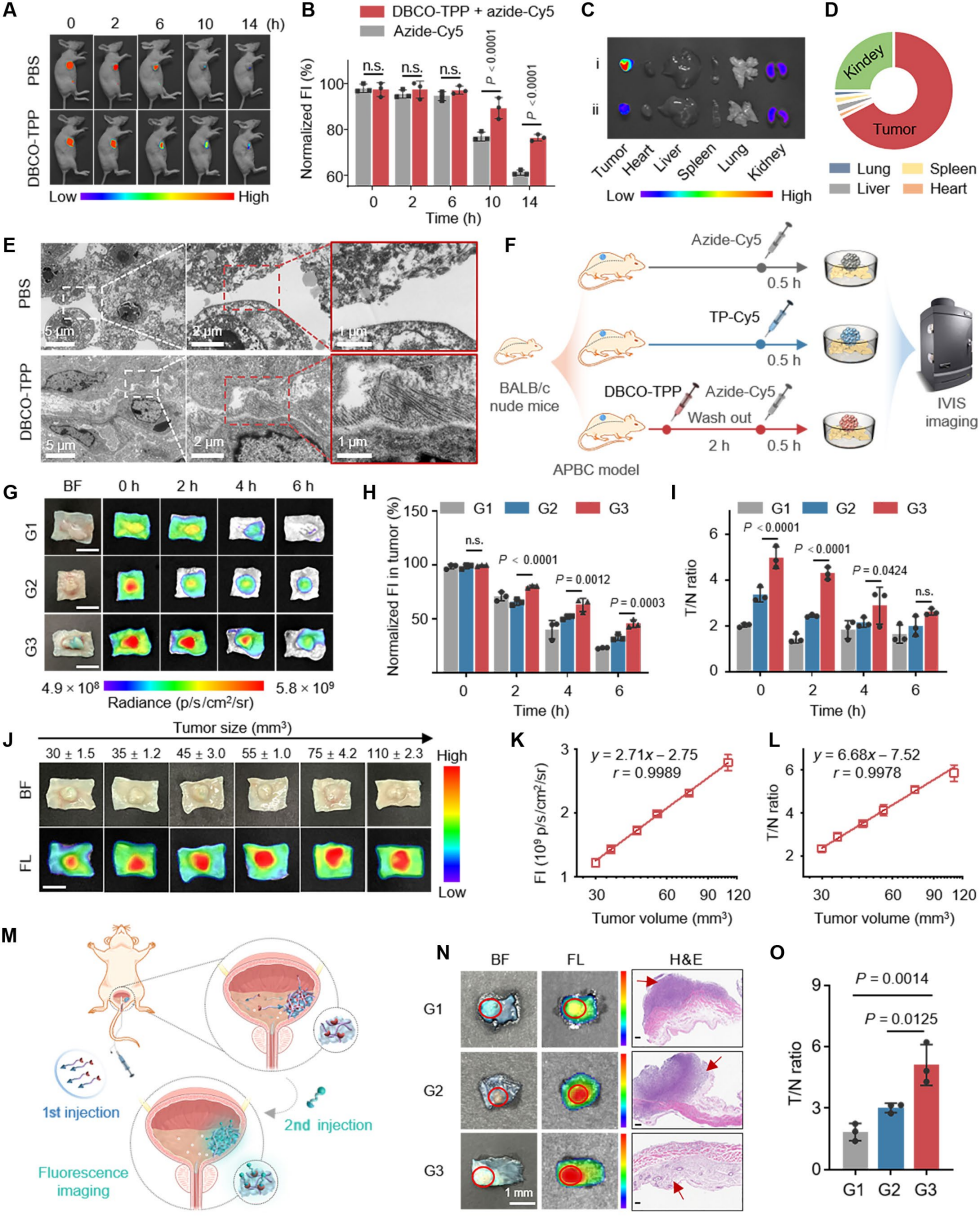
In vivo evaluation of tumor imaging in APBC and orthotopic models. (**A**) Representative IVIS images and (**B**) corresponding normalized FI of mice following intratumoral injection of DBCO-TPP + azide-Cy5 or azide-Cy5. (**C**) Ex vivo imaging of tumors and major organs 10 hours after intratumoral injection. (**D**) Quantitative analysis of FI in tumors after 10 hours. (**E**) Bio-TEM images of EJ tumor after treatment with PBS and DBCO-TPP. (**F**) Schematic illustration of imaging in the APBC model. (**G**) IVIS images, (**H**) corresponding normalized FI, and the (**I**) T/N ratio of APBC imaged with G1 (azide-Cy5, 10 μM), G2 (TP-Cy5, 10 μM), and G3 (DBCO-TPP, 50 μM + azide-Cy5, 10 μM) and then irradiated with a 660-nm laser for 0 min and 2, 4, and 6 hours. Scale bars, 2 mm. (**J**) IVIS images, (**K**) corresponding normalized FI, and (**L**) the T/N ratio of APBC models with varying tumor volumes imaged with G3 (DBCO-TPP, 50 μM + azide-Cy5, 10 μM). Scale bar, 2 mm. (**M**) Schematic illustration of imaging in the orthotopic bladder cancer model. (**N**) IVIS images and (**O**) the corresponding T/N ratio of orthotopic bladder cancer imaged with G1 (azide-Cy5, 10 μM), G2 (TP-Cy5, 10 μM), and G3 (DBCO-TPP, 50 μM + azide-Cy5, 10 μM). Data (*n* = 3) are expressed as means ± SD, and statistical differences were analyzed using the two-way ANOVA method. n.s. represents no statistical difference.

To evaluate the targeting capability and retention effect of DBCO-TPP in vivo, we intratumorally injected DBCO-TPP (100 μM, 50 μl) into EJ tumor-bearing mice and then administered azide-Cy5 (10 μM, 50 μl) after 30 min. In the control group, we directly administered the same dose of azide-Cy5. In vivo imaging system (IVIS) imaging showed that the DBCO-TPP + azide-Cy5 group exhibited a more prolonged and stable fluorescence signal in the tumor region. The FI in the DBCO-TPP + azide-Cy5 group remained almost unchanged for 8 hours and retained 83% of its initial intensity after 12 hours. In contrast, the FI in the control group began to decline after 6 hours and decreased to 72% at 12 hours (Fig. 6, A and B, and fig. S46). Moreover, ex vivo imaging of tumors and major organs collected 10 hours postadministration revealed that the fluorescence signal in tumors increased notably, showing a 2.5-fold enhancement compared to the control group (Fig. 6, C and D). Because of transdermal absorption and renal clearance of fluorescent molecules, we observed some accumulation in the kidneys, whereas we detected no notable fluorescence in other major organs (fig. S47). Bio-TEM analysis of tumor sections after 2 hours of DBCO-TPP perfusion showed abundant nanofibers in the ECM, whereas the control mice showed none (Fig. 6E and fig. S48). Thus, we speculate that enhanced retention results from DBCO-TPP self-assembly, where the resulting BCAR covalently captures azide-Cy5 via a bioorthogonal reaction and prevents its rapid clearance.

We established an APBC model in female BALB/c nude mice to evaluate the performance and photostability of the PRE-targeted platform in tumor imaging (fig. S49A). As shown in Fig. 6F, in group 1 (G1; azide-Cy5, 10 μM) and group 2 (G2; TP-Cy5, 10 μM), we injected probes for 30 min and then washed them out. In group 3 (G3; DBCO-TPP + azide-Cy5), we injected DBCO-TPP (50 μM) into the air-pouch for 2 hours and then washed it, followed by azide-Cy5 injection for 30 min and another wash. We then dissected the air-pouch and performed IVIS imaging, which revealed stronger fluorescence signals in G3 compared to other groups. Because DBCO-TPP forms nanofibers that construct BCARs and increase binding sites, the FI in G3 was 2.0-fold higher than in G2 and 3.8-fold higher than in the nontargeting Cy5 group.

In clinical bladder cancer surgery, continuous light exposure can cause photobleaching of fluorescent molecules. To assess photostability, we applied 660nm irradiation for 0 to 6 hours to simulate fluorescence cystoscopy (*27*). Over time, G3 maintained stronger tumor fluorescence signals than G1 and G2 (Fig. 6G and fig. S50). Quantitative analysis showed that, after 6 hours, G3 retained 46% of its initial FI, compared to 33 and 23% in G2 and G1, respectively (Fig. 6H). The photostability of azide-Cy5 in G3 was 2.0-fold higher than in G1, consistent with MCT data, confirming that in situ nanofiber formation enhances stability via β sheet structures. In addition, at 2 hours postinjection, G3 achieved a T/N tissue ratio of 4.3, representing a 1.8- and 2.9-fold increase compared to G2 and G1, respectively (Fig. 6I). These results demonstrate that the PRE-targeted platform notably improves imaging quality. Compared with receptor-targeted probes, the T/N ratio increased by 1.7-fold, providing improved support for intraoperative navigation (fig. S51).

Building on the enhanced FI and long-lasting photostability of the PRE-targeted platform, we further applied it to monitor APBC tumor of varying sizes. As shown in Fig. 6J, the IVIS imaging showed tumors with mean volumes ranging from 30 ± 1.5 mm^3^ to 110 ± 2.3 mm^3^. Notably, as the tumor volume increased, both the mean FI and the T/N ratio displayed a strong linear relationship, demonstrating a positive correlation with the logarithm of tumor volume (Fig. 6, K and L). This linear correlation highlights robust capability of the PRE-targeted platform in detecting tumor lesions in live mice, as well as its potential for evaluating tumor size in orthotopic bladder cancer models.

To achieve a more comprehensive investigation into the precise localization of bladder cancer, we established an orthotopic T24 bladder cancer mouse model (Fig. 6M and fig. S52A). Mice treated with G3 showed strong fluorescence signals, with a T/N ratio of 5.1, clearly delineating the tumor boundary. In contrast, mice treated with G2 (T/N ratio: 3.0) or G1 (T/N ratio: 1.8) failed to effectively distinguish the tumor margin (Fig. 6, N and O, and fig. S52B). Further analysis revealed that the T/N ratio of G3 was 1.7 times higher than that of G2, confirming that the DBCO-TPP–formed BCAR on the cell surface of tumor plays a critical role in enhancing imaging performance.

Last, after imaging the orthotopic model, we collected major organs (heart, liver, spleen, lungs, and kidneys) for histological evaluation using hematoxylin and eosin (H&E) staining. The H&E results revealed no notable histopathological abnormalities (fig. S52C), and similar findings were observed in the APBC model (fig. S52D). Furthermore, to assess the potential in vivo toxicity of DBCO-TPP combined with azide-Cy5, we collected serum samples for biochemical analysis. Liver and kidney function markers, including alanine aminotransferase (ALT), aspartate aminotransferase (AST), alkaline phosphatase (ALP), blood urea nitrogen (BUN), and creatinine (CRE) remained within normal ranges in both the PBS group and DBCO-TPP + azide-Cy5 group (fig. S53). In addition, the results of blood routine examination, including red blood cell (RBC), white blood cell (WBC), platelet (PLT) and hemoglobin (HGB), total protein (TP), albumin (ALB), and globulin (GLOB), showed no notable differences between the two groups (fig. S54). Collectively, these results demonstrate that DBCO-TPP + azide-Cy5 exhibits excellent biocompatibility in vivo, supporting its potential for clinical translation in biomedical applications.

### Ex vivo imaging of human bladder tumor via the PRE-targeted platform

**Fig. 7.**
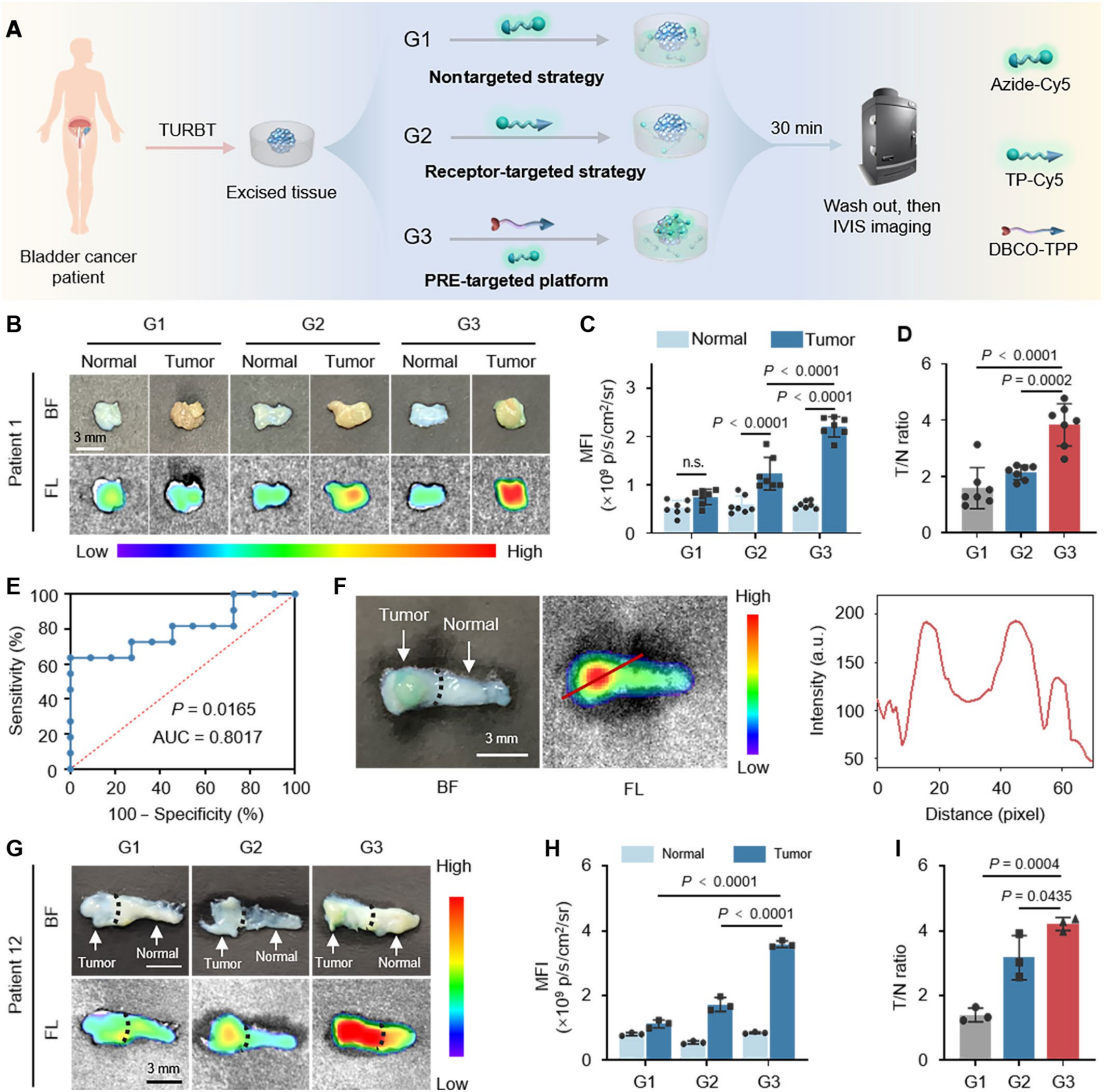
Evaluation of the PRE-targeted platform for imaging of human bladder tumor specimens. (**A**) Schematic illustration of ex vivo imaging of human bladder tumor. (**B**) White light, fluorescence images and H&E images of freshly isolated normal and tumor bladder tissues incubated with azide-Cy5 (G1), TP-Cy5 (G2), and DBCO-TPP + azide-Cy5 (G3), along with (**C**) the corresponding MFI and (**D**) T/N ratio. Data are presented as means ± SD and were analyzed by the two/one-way ANOVA. *n* = 7. (**E**) Receiver operating characteristic (ROC) analysis and AUC of normal and tumor bladder tissue G3 imaging intensity. *n* = 11. (**F**) Fluorescence image and signal intensity of resected transition zone tissue from the patient and the FI under the red line. (**G**) Tumor margin fluorescence imaging in G1, G2, and G3, along with (**H**) the corresponding MFI and (**I**) T/N ratio. Data (*n* = 3) are presented as means ± SD and were analyzed by the two/one-way ANOVA. n.s. represents no statistical difference.

Our goal is to develop a method for providing case validation results during surgery without affecting tissue integrity or morphology, assisting medical professionals in pathological diagnosis and margin assessment. Initially, to evaluate the diagnostic accuracy of the imaging strategy in identifying tumor tissue, including true positive and false positive rates, we enrolled 11 patients with pathologically confirmed bladder cancer who underwent surgical resection. During the procedure, we collected tumor and normal tissues and divided them into three groups for different treatments. For G3, we soaked samples in DBCO-TPP (50 μM) for 2 hours, washed them, and then incubated them with azide-Cy5 (10 μM) for 30 min, followed by a final wash. For G2, we treated samples directly with TP-Cy5 (10 μM) for 30 min, whereas for G1, we soaked samples in azide-Cy5 (10 μM) for 30 min. After treatment, we measured fluorescence signals using the IVIS spectrum system to distinguish tumor from normal tissues (Fig. 7A). Ex vivo staining with the nontargeted G1 showed that the mean fluorescence intensity (MFI) of bladder tumors was similar to that of normal tissues (*P* = 0.1225), indicating minimal discriminatory capability. In contrast, G2 demonstrated a moderate ability to differentiate tumor from normal tissue (1.24 ± 0.34 × 10^9^ versus 0.59 ± 0.19 × 10^9^, *P* < 0.0001), whereas G3 exhibited a notably enhanced distinction, reflecting superior tumor-targeting performance (2.2 ± 0.2 × 10^9^ versus 0.59 ± 0.08 × 10^9^, *P* < 0.0001) (Fig. 7, B and C). Collectively, as a conventional fluorescence probe targeting strategy, the receptor-targeted probe approach used in G2 showed a slightly higher T/N ratio compared to the nontargeted G1 (1.4-fold), indicating a certain level of imaging capability. However, G3, which involved an increased number of artificial targets (BCARs), demonstrated enhanced tumor selectivity, with a notably higher T/N ratio than G1, improving by 2.4-fold (Fig. 7D and fig. S55). Moreover, we observed higher CD44v6 expression in tumor tissues than in normal tissues (figs. S56 and S57). Furthermore, analysis of all patient samples identified two false-negative and one false-positive cases (table S2), with an area under the curve (AUC) value of 0.8017 (95% confidence interval: 0.6085 to 0.9948) (Fig. 7E) (*53*). In the cohort of patients with bladder cancer, the method exhibited a sensitivity of 81.8% and a specificity of 90.9% (*P* = 0.0165). Further analysis indicated that compared to true-positive samples, false-negative cases showed relatively lower CD44v6 expression in tumor tissues.

Precise surgical resection is crucial for treating bladder cancer, but incomplete removal at the tumor margins remains a notable challenge, often leading to recurrence. To further demonstrate the high spatial resolution of the PRE-targeted platform, we collected transition zone tissues for analysis. IVIS imaging showed that ex vivo stained tissues exhibited both high- and low-fluorescence regions. As shown in Fig. 7F, we drew a line and quantified signal intensity, which revealed higher fluorescence in tumor interiors and lower intensity at tumor margins.

To further evaluate the differences in tumor margin imaging between the receptor-targeted strategy and our proposed PRE-targeted platform, samples from three cases were collected and assigned to different groups for comparative analysis. IVIS imaging revealed that G3 exhibited clear delineation between tumor and normal tissues (3.59 ± 0.10 × 10^9^ versus 0.85 ± 0.03 × 10^9^, *P* < 0.0001), with a T/N ratio of G3 (4.2 ± 0.2) 1.3 times higher than that of G2 (3.2 ± 0.7) and 3.0 times higher than G1 (1.4 ± 0.2). In G2, tumor tissue remained detectable (1.73 ± 0.22 × 10^9^ versus 0.56 ± 0.05 × 10^9^, *P* < 0.0001), but margins appeared indistinct, whereas in G1 (1.12 ± 0.13 × 10^9^ versus 0.81 ± 0.05 × 10^9^, *P* = 0.0163), we could not distinguish tumor boundaries (Fig. 7, G to I). These findings suggest that, during surgery, PRE-targeted platform probe incubation enables rapid labeling and imaging of CD44v6-expressing bladder cancer tissues, providing an effective tool for precise intraoperative tumor margin identification.

## DISCUSSION

We analyzed the expression of CD44v6 (a variant of human CD44 containing exon 11) in bladder cancer using eukaryotic transcriptome analysis based on clinical specimens. Through a systematic RNA-seq–based screening workflow, we identified CD44v6 as a candidate target due to its differential expression, tumor specificity, and membrane localization. The results showed that the splicing pattern of CD44 in primary tumors exhibited notable differences compared to normal tissues. In normal tissues, variable exons (6 to 15) remained almost undetectable, whereas bladder cancer tissues exhibited notably increased AS events. We found that high CD44v6 expression correlates with tumor invasiveness, metastasis, and poor prognosis, supporting its biological relevance and highlighting its suitability as a tumor-specific imaging target. These findings suggest that CD44v6 may serve as a potential therapeutic target for bladder cancer and warrants further clinical investigation.

Clinical and preclinical studies have demonstrated that fluorescent probes targeting tumor-specific biomarkers can enhance tumor specificity and improve diagnostic accuracy (*12*). However, commercially available targeted fluorescent probes still face two major challenges: first, the limited expression of tumor-specific biomarkers on the cell membrane, resulting in weak fluorescence signals; second, the relatively large size of fluorescent dyes reduces incorporation efficiency and promotes photobleaching during surgery. To address these issues, we leveraged the advantages of in vivo self-assembly and bioorthogonal conjugation strategies to develop a PRE-targeted platform. This platform enables DBCO-TPP to selectively and rapidly embed high-density BCARs onto the tumor cell membrane in a highly dynamic and complex in vivo environment, thereby increasing the number of bioorthogonal handles (DBCO) on the tumor cell surface and improving the labeling efficiency of azide-Cy5. To further investigate how DBCO-TPP self-assembles into fibers and embeds into the cell membrane, we used MD simulations to elucidate the molecular interactions between DBCO-RSP and phospholipid membranes and validated these findings through SEM and CLSM experiments.

In this study, we evaluated two CD44v6-targeted imaging strategies: a receptor-targeted strategy, in which we covalently linked a CD44v6-targeted peptide to Cy5 (TP-Cy5), relying on the inherent specific target (CD44v6) on the tumor cell surface for imaging, and a PRE-targeted platform, where DBCO-TPP was first assembled in situ on the tumor cell membrane, increasing the number of DBCO sites, followed by covalent labeling with azide-Cy5 via bioorthogonal reaction to enhance fluorescent probe accumulation. In MCT model, CLSM results showed that DBCO-TPP notably increased the number of specific targets (bioorthogonal handles) in the tumor region, and when treated with equal doses of azide-Cy5 and TP-Cy5, azide-Cy5–labeled tumors exhibited superior imaging performance. In a xenograft tumor model, we further confirmed that DBCO-TPP effectively prolonged the retention time of fluorescent molecules in tumors, with the FI being 1.3-fold higher than the control group at 14 hours.

During bladder cancer surgery, prolonged exposure to light may lead to photobleaching of fluorescent molecules (*54*). To evaluate the antiphotobleaching advantages of the PRE-targeted platform, we established an APBC and orthotopic mouse model. In the APBC, the PRE-targeted platform maintained 81% FI after 2 hours, which is sufficient for most bladder cancer surgeries (*55*). In addition, in bladder cancer monitoring with air-pouch tumors of different sizes, when the tumor volume ranged from 30 ± 1.5 mm^3^ to 110 ± 2.3 mm^3^, both the average FI and the T/N ratio exhibited strong linear correlations with the logarithm of tumor volume, with correlation coefficients of 0.9989 and 0.9978, respectively. To further investigate the precise localization of bladder cancer, we established an orthotopic T24 bladder cancer model to better simulate human conditions, and the results demonstrated that, compared to the receptor-targeted strategy, the PRE-targeted platform exhibited a higher T/N ratio (increased by 1.3-fold) and clearer tumor margins.

Although fluorescent probe development has advanced notably, in vivo fluorescence-guided surgery still remains influenced by vascular dynamics, and systemic administration leads to nonspecific uptake (*56*). Moreover, regulatory agencies require extensive safety evaluations before fluorescent contrast agents can enter clinical trials, which makes their clinical translation a lengthy and costly process. Therefore, a molecular imaging technique that enables rapid imaging without systemic administration could notably affect bladder cancer resection surgery (*57*, *58*). To this end, we developed a bladder perfusion imaging technique and investigated its potential application in human bladder cancer resection specimens. The results showed that the PRE-targeted platform effectively distinguished tumor tissues from normal tissues, with an AUC value of 0.8017 for tumor versus nontumor discrimination. Histopathological analysis further confirmed the colocalization of fluorescence signals with tumor tissues. Moreover, the optimized rapid imaging process required only 30 min to complete intraoperative margin assessment. Compared to existing tumor margin detection methods, this technique offers several advantages, including high-resolution, wide-field imaging of entire tissue specimens.

Despite its advantages, this study has some limitations. First, this is a single-center clinical study with a small sample size, which may affect the statistical power. Therefore, future multicenter studies are needed to include patients with bladder cancer with different molecular subtypes and varying levels of CD44v6 expression. Second, ∼18% of patients with bladder cancer may be CD44v6 negative, although our approach enhances target abundance, it does not fully address intratumoral heterogeneity. Furthermore, although the ex vivo rapid incubation imaging process requires only 30 min, further optimization of the staining protocol is needed to accelerate clinical decision making.

In conclusion, our study demonstrates that the PRE-targeted platform can be used for noninvasive bladder cancer imaging and enables precise intraoperative visualization of malignant lesions. This technology could assist surgeons in real-time determination of surgical margins and optimizes bladder cancer resection strategies. In the future, it may potentially replace intraoperative white-light navigation and improve the precision management of bladder cancer.

## MATERIALS AND METHODS

### Study design

The aim of our study is to develop a peptide recognition enhanced targeted imaging platform through in situ self-assembly of BCARs on tumor cell surfaces. We first used human-derived samples to investigate the role of CD44v6 in bladder cancer initiation and progression and to assess its potential as a biomarker. On the basis of these findings, we rationally designed and synthesized peptides with specific recognition ability toward CD44v6 to assemble artificial receptors on the cell membrane. We systematically explored the theoretical basis and experimental mechanisms underlying BCAR construction under dynamic and complex in vivo conditions. We further evaluated the platform in multiple animal models, including xenograft, balloon, and orthotopic bladder cancer models. Last, we assessed its clinical applicability using surgical specimens from 14 patients with bladder cancer and randomly assigned them into experimental groups under a single-blind design.

### Patient tissue sample collection and RNA-seq analysis

We enrolled a total of 22 tissues from patients who underwent radical cystectomy and received a pathological diagnosis of NMIBC at the Department of Urology, Jiangbei Campus of the First Affiliated Hospital of Army Medical University. At least two experienced pathologists confirmed the pathological diagnosis using formalin-fixed and paraffin-embedded tissue blocks and classified tumors according to established histopathological criteria. Detailed patient characteristics are provided in fig. S58 and table S3. For transcriptomic profiling, we extracted total RNA from bladder cancer tissues (*n* = 11) and normal bladder epithelial tissues (*n* = 11). We then purified, fragmented, reverse transcribed, and amplified the RNA samples to construct RNA-seq libraries. We performed sequencing on the Illumina PE150 platform at a depth of 100 million reads per sample. Subsequently, we conducted quality control and alignment to the reference genome, followed by AS analysis. Last, we identified DEGs using statistical methods and performed functional enrichment analysis using GO and KEGG pathways.

### Fluorescence imaging of isolated human bladder tissue

We collected 11 bladder tumor tissues and matched normal bladder epithelial tissues from patients undergoing radical cystectomy at the Department of Urology, Jiangbei Campus, First Affiliated Hospital of Army Medical University. Detailed patient characteristics are provided in fig. S59 and table S4. Immediately after surgical resection, we immersed the specimens in RNAlater solution for preservation. Before analysis, we rinsed the tissues three times with PBS to remove residual RNAlater and then divided them evenly into three groups (G1, G2, and G3). For G3, we immersed samples in DBCO-TPP (50 μM) for 2 hours and then washed them with PBS and incubated them in azide-Cy5 solution in the dark for 30 min. For G2 and G1, we incubated samples in the dark with TP-Cy5 (20 μM) and azide-Cy5 (10 μM), respectively, for 30 min. We then thoroughly rinsed all samples with PBS and removed residual surface liquid using filter paper. Last, we performed fluorescence imaging using an IVIS with standardized parameters: excitation at 605 nm, emission at 670 nm, and an exposure time of 1 s.

### Statistical analysis

All chemical structure formulas were drawn using the ChemDraw 19.0 software. All data were plotted using Origin and GraphPad Prism 8, and the results are expressed as the means ± SD. The difference between the two groups was analyzed by the unpaired two-tailed Student’s *t* test. Statistically significant differences between multiple groups were assessed using one-way analysis of variance (ANOVA), followed by either a Tukey’s or a Dunnett’s multiple comparisons test. Alternatively, a two-way ANOVA was performed followed by a Sidak’s multiple comparisons test. The sample size of each experiment, specific statistical tests, and statistical analyses are detailed in the figure caption. The threshold for statistical significance is *P* < 0.05. n.s. represents no statistical difference.

### Ethics statement

This study involves with patients with NMIBC and was approved by the Jiangbei Campus of the First Affiliated Hospital of Army Medical University (ER2024KY023), and informed consent was obtained at the time of collection of human tissue sample. All experimental animals were female BALB/c nude mice aged 6 to 8 weeks and weighing 18 to 24 g. The experimental protocols were approved by the Institution Animal Care and Use of Chongqing Medical University (IACUC-CQMU-2024-04057), and all procedures were performed in accordance with the Animal Ethics Committee guidelines.

## References

[1] A. T. Lenis, P. M. Lec, K. Chamie, M. D. Mshs, Bladder cancer: A review. JAMA 324, 1980–1991 (2020). 33201207 10.1001/jama.2020.17598

[2] E. Compérat, M. B. Amin, R. Cathomas, A. Choudhury, M. De Santis, A. Kamat, A. Stenzl, H. C. Thoeny, J. A. Witjes, Current best practice for bladder cancer: A narrative review of diagnostics and treatments. Lancet 400, 1712–1721 (2022). 36174585 10.1016/S0140-6736(22)01188-6

[3] J. D. Subiela, Ó. Rodríguez Faba, F. Guerrero-Ramos, J. Aumatell, A. Breda, J. Palou, Carcinoma in situ of the bladder: Why is it underdetected? Curr. Opin. Urol. 30, 329–399 (2020). 10.1097/MOU.0000000000000758 32235280

[4] C. L. Chaffer, R. A. Weinberg, A perspective on cancer cell metastasis. Science 331, 1559–1564 (2011). 21436443 10.1126/science.1203543

[5] S. Jiang, J. Wang, C. Yang, R. Tan, J. Hou, Y. Shi, H. Zhang, S. Ma, J. Wang, M. Zhang, G. Philips, Z. Li, J. Ma, W. Yu, G. Wang, Y. Wu, R. Schlegel, H. Wang, S. Cao, J. Guo, X. Liu, Y. Dang, Continuous culture of urine-derived bladder cancer cells for precision medicine. Protein Cell 10, 902–907 (2019). 31347094 10.1007/s13238-019-0649-5 PMC6881267

[6] J. Wang, J. Zhu, J. Hu, Z. Wang, X. Wang, J. Pan, Y. Chu, Z. Li, W. Jiang, C. Liang, J. Hou, J. Guo, Y. Dang, S. Jiang, A novel in vitro prognostic model of bladder cancer based on urine-derived living tumor cells. Genes Dis. 10, 2586–2596 (2023). 37554182 10.1016/j.gendis.2022.10.022 PMC10405094

[7] J. D. Predina, A. D. Newton, C. Connolly, A. Dunbar, M. Baldassari, C. Deshpande, E. Cantu, J. Stadanlick, S. A. Kularatne, P. S. Low, S. Singhal, Identification of a folate receptor-targeted near-infrared molecular contrast agent to localize pulmonary adenocarcinomas. Mol. Ther. 26, 390–403 (2018). 29241970 10.1016/j.ymthe.2017.10.016 PMC5835020

[8] H. Miyamoto, Intraoperative pathology consultation during urological surgery: Impact on final margin status and pitfalls of frozen section diagnosis. Pathol. Int. 71, 567–580 (2021). 34154033 10.1111/pin.13132

[9] S. B. Williams, M. B. Gavaghan, A. Fernandez, S. Daneshmand, A. M. Kamat, Macro and microeconomics of blue light cystoscopy with CYSVIEW® in non-muscle invasive bladder cancer. Urol. Oncol. 40, 10.e7–10.e12 (2022). 10.1016/j.urolonc.2021.05.023 34158205

[10] Y. Lotan, T. J. Bivalacqua, T. Downs, W. Huang, J. Jones, A. M. Kamat, B. Konety, P.-U. Malmström, J. McKiernan, M. O’Donnell, S. Patel, K. Pohar, M. Resnick, A. Sankin, A. Smith, G. Steinberg, E. Trabulsi, M. Woods, S. Daneshmand, Blue light flexible cystoscopy with hexaminolevulinate in non-muscle-invasive bladder cancer: Review of the clinical evidence and consensus statement on optimal use in the USA—Update 2018. Nat. Rev. Urol. 16, 377–386 (2019). 31019310 10.1038/s41585-019-0184-4 PMC7136177

[11] A. Refaat, M. L. Yap, G. Pietersz, A. P. G. Walsh, J. Zeller, B. del Rosal, X. Wang, K. Peter, In vivo fluorescence imaging: Success in preclinical imaging paves the way for clinical applications. J. Nanobiotechnol. 20, 450 (2022). 10.1186/s12951-022-01648-7 PMC9571426 36243718

[12] T. Etrych, O. Janoušková, P. Chytil, Fluorescence imaging as a tool in preclinical evaluation of polymer-based nano-DDS systems intended for cancer treatment. Pharmaceutics 11, 471 (2019). 31547308 10.3390/pharmaceutics11090471 PMC6781319

[13] C. Li, J. Liu, X. Yang, Q. Yang, W. Huang, M. Zhang, D. Zhou, R. Wang, J. Gong, Q. Miao, L. Kang, J. Yang, Theranostic application of ^64^Cu/^177^Lu-labeled anti-Trop2 monoclonal antibody in pancreatic cancer tumor models. Eur. J. Nucl. Med. Mol. Imaging 50, 168–183 (2022). 36063202 10.1007/s00259-022-05954-y

[14] R. Liu, Y. Xu, K. Xu, Z. Dai, Current trends and key considerations in the clinical translation of targeted fluorescent probes for intraoperative navigation. Aggregate 2, e23 (2021).

[15] D. Seah, Z. Cheng, M. Vendrell, Fluorescent probes for imaging in humans: Where are we now? ACS Nano 17, 19478–19490 (2023). 37787658 10.1021/acsnano.3c03564 PMC10604082

[16] Y. Li, S. Shan, R. Zhang, C. Sun, X. Hu, J. Fan, Y. Wang, R. Duan, M. Gao, Imaging and downstaging bladder cancer with the ^177^Lu-labeled bioorthogonal nanoprobe. ACS Nano 18, 17209–17217 (2024). 38904444 10.1021/acsnano.4c04303

[17] H.-W. An, D.-Y. Hou, J. Yang, Z.-Q. Wang, M.-D. Wang, R. Zheng, N.-Y. Zhang, X.-J. Hu, Z.-J. Wang, L. Wang, D. Liu, J.-F. Hao, W. Xu, Y. Zhao, H. Wang, A bispecific glycopeptide spatiotemporally regulates tumor microenvironment for inhibiting bladder cancer recurrence. Sci. Adv. 9, eabq8225 (2023). 36857458 10.1126/sciadv.abq8225 PMC9977173

[18] Y.-X. Lin, Y. Wang, J. Ding, A. Jiang, J. Wang, M. Yu, S. Blake, S. Liu, C. J. Bieberich, O. C. Farokhzad, L. Mei, H. Wang, J. Shi, Reactivation of the tumor suppressor PTEN by mRNA nanoparticles enhances antitumor immunity in preclinical models. Sci. Transl. Med. 13, eaba9772 (2021). 34162754 10.1126/scitranslmed.aba9772 PMC8284983

[19] Y. Dong, Z. Mu, Y. Wu, H. Hu, J. Zuo, Y. Li, S. Wang, Z. Wang, L. Bai, H. Liang, Self-assembly of artificial topological nanostructures for enhanced bioorthogonal activation and drug penetration for cancer immunotherapy. J. Am. Chem. Soc. 147, 39547–39560 (2025). 40977088 10.1021/jacs.5c12835

[20] Q. Li, Z. Mu, Y. Dong, Z. Ouyang, J. Zuo, Y. Wu, Y. Yang, S. Sun, H. Liang, L. Bai, In situ self-assembly of artificial topological nanostructures enhances in vivo efficacy of PCSK9 inhibitory peptides. Angew. Chem. Int. Ed. Engl. 64, e202502559 (2025). 39945488 10.1002/anie.202502559

[21] D.-Y. Hou, D.-B. Cheng, N.-Y. Zhang, Z.-J. Wang, X.-J. Hu, X. Li, M.-Y. Lv, X.-P. Li, L.-R. Jian, J.-P. Ma, T. Sun, Z.-Y. Qiao, W. Xu, H. Wang, In vivo assembly enhanced binding effect augments tumor specific ferroptosis therapy. Nat. Commun. 15, 454 (2024). 38212623 10.1038/s41467-023-44665-2 PMC10784468

[22] D. Zhang, G. B. Qi, Y. X. Zhao, S. L. Qiao, C. Yang, H. Wang, In situ formation of nanofibers from purpurin18-peptide vonjugates and the sssembly induced tetention effect in tumor sites. Adv. Mater. 27, 6125–6130 (2015). 26350172 10.1002/adma.201502598

[23] H.-W. An, L.-L. Li, Y. Wang, Z. Wang, D. Hou, Y.-X. Lin, S.-L. Qiao, M.-D. Wang, C. Yang, Y. Cong, Y. Ma, X.-X. Zhao, Q. Cai, W.-T. Chen, C.-Q. Lu, W. Xu, H. Wang, Y. Zhao, A tumour-selective cascade activatable self-detained system for drug delivery and cancer imaging. Nat. Commun. 10, 4861 (2019). 31649241 10.1038/s41467-019-12848-5 PMC6813295

[24] H.-W. An, D.-Y. Hou, N.-Y. Zhang, X.-J. Hu, L. Yi, J.-X. Liang, Y.-X. Liu, Y.-J. Zhang, Y.-S. Liao, W. Xu, H. Wang, A self-assembled fluorescent contrast agent targeting XIAP for image-guided surgery of bladder cancer. Nano Today 56, 102313 (2024).

[25] A. M. Valencia-Hernandez, W. Y. Ng, N. Ghazanfari, S. Ghilas, M. N. de Menezes, L. E. Holz, C. Huang, K. English, M. Naung, P. S. Tan, K. M. Tullett, T. M. Steiner, M. H. Enders, L. Beattie, Y. C. Chua, C. M. Jones, A. Cozijnsen, V. Mollard, Y. Cai, D. G. Bowen, A. W. Purcell, N. L. La Gruta, J. A. Villadangos, T. de Koning-Ward, A. E. Barry, W. Barchet, I. A. Cockburn, G. I. McFadden, S. Gras, M. H. Lahoud, P. Bertolino, R. B. Schittenhelm, I. Caminschi, W. R. Heath, D. Fernandez-Ruiz, A natural peptide antigen within the plasmodium ribosomal protein RPL6 confers liver TRM cell-mediated immunity against malaria in mice. Cell Host Microbe 27, 950–962.e7 (2020). 32396839 10.1016/j.chom.2020.04.010

[26] A. Winnicka, J. Brzeszczyńska, J. Saluk, P. Wigner-Jeziorska, Nanomedicine in bladder cancer therapy. Int. J. Mol. Sci. 25, 10388 (2024). 39408718 10.3390/ijms251910388 PMC11476791

[27] Z. Wang, C. Zhao, Y. Li, J. Wang, D. Hou, L. Wang, Y. Wang, X. Wang, X. Liu, H. Wang, W. Xu, Photostable cascade-activatable peptide self-assembly on a cancer cell membrane for high-performance identification of human bladder cancer. Adv. Mater. 35, e2210732 (2023). 37172955 10.1002/adma.202210732

[28] M. Xia, Q. Wang, Y. Liu, C. Fang, B. Zhang, S. Yang, F. Zhou, P. Lin, M. Gu, C. Huang, X. Zhang, F. Li, H. Liu, G. Wang, D. Ling, Self-propelled assembly of nanoparticles with self-catalytic regulation for tumour-specific imaging and therapy. Nat. Commun. 15, 460 (2024). 38212655 10.1038/s41467-024-44736-y PMC10784296

[29] B. J. Kim, B. Xu, Enzyme-instructed self-assembly for cancer therapy and imaging. Bioconjug. Chem. 31, 492–500 (2020). 31995365 10.1021/acs.bioconjchem.0c00025 PMC7082204

[30] Y. Yuan, J. Zhang, X. Qi, S. Li, G. Liu, S. Siddhanta, I. Barman, X. Song, M. T. McMahon, J. W. M. Bulte, Furin-mediated intracellular self-assembly of olsalazine nanoparticles for enhanced magnetic resonance imaging and tumour therapy. Nat. Mater. 18, 1376–1383 (2019). 31636420 10.1038/s41563-019-0503-4 PMC6872935

[31] D. Ye, A. J. Shuhendler, L. Cui, L. Tong, S. S. Tee, G. Tikhomirov, D. W. Felsher, J. Rao, Bioorthogonal cyclization-mediated in situ self-assembly of small-molecule probes for imaging caspase activity in vivo. Nat. Chem. 6, 519–526 (2014). 24848238 10.1038/nchem.1920 PMC4031611

[32] Y. Gao, J. Shi, D. Yuan, B. Xu, Imaging enzyme-triggered self-assembly of small molecules inside live cells. Nat. Commun. 3, 1033 (2012). 22929790 10.1038/ncomms2040 PMC3521559

[33] X. Zheng, H. Mao, D. Huo, W. Wu, B. Liu, X. Jiang, Successively activatable ultrasensitive probe for imaging tumour acidity and hypoxia. Nat. Biomed. Eng. 1, 0057 (2017).

[34] Y. Miao, Y. Wang, Y. Chen, Z. Huang, C. Lu, Y. Liu, F. Chen, X. Wen, J. Zhang, S. Zhu, P. Zhao, Y. Chen, T. Tian, Y. Zhang, H. Xie, J. Lin, D. Ye, Pretargeted multimodal tumor imaging by enzymatic self-immobilization labeling and bioorthogonal reaction. J. Am. Chem. Soc. 147, 2809–2821 (2025). 39801138 10.1021/jacs.4c15896

[35] H. Xu, M. Niu, X. Yuan, K. Wu, A. Liu, CD44 as a tumor biomarker and therapeutic target. Exp. Hematol. Oncol. 9, 36 (2020). 33303029 10.1186/s40164-020-00192-0 PMC7727191

[36] P. Rajan, D. J. Elliott, C. N. Robson, H. Y. Leung, Alternative splicing and biological heterogeneity in prostate cancer. Nat. Rev. Urol. 6, 454–460 (2009). 19657379 10.1038/nrurol.2009.125

[37] O. M. Omran, H. S. Ata, CD44s and CD44v6 in diagnosis and prognosis of human bladder cancer. Ultrastruct. Pathol. 36, 145–152 (2012). 22559040 10.3109/01913123.2011.651522

[38] V. Toma, D. Hauri, U. Schmid, D. Ackermann, R. Maurer, G. Alund, H. Knönagel, M. Rist, T. C. Gasser, G. Sauter, J. Roth, Focal loss of CD44 variant protein expression is related to recurrence in superficial bladder carcinoma. Am. J. Pathol. 155, 1427–1432 (1999). 10550296 10.1016/S0002-9440(10)65455-7 PMC1866967

[39] R. Szatanek, M. Baj-Krzyworzeka, CD44 and tumor-derived extracellular vesicles (TEVs). Possible gateway to cancer metastasis. Int. J. Mol. Sci. 22, 1463 (2021). 33540535 10.3390/ijms22031463 PMC7867195

[40] C. Chen, S. Zhao, A. Karnad, J. W. Freeman, The biology and role of CD44 in cancer progression: Therapeutic implications. J. Hematol. Oncol. 11, 64 (2018). 29747682 10.1186/s13045-018-0605-5 PMC5946470

[41] Y. Xu, Z. Bai, T. Lan, C. Fu, P. Cheng, CD44 and its implication in neoplastic diseases. MedComm 5, e554 (2024). 38783892 10.1002/mco2.554 PMC11112461

[42] K. Nam, S. Oh, I. Shin, Ablation of CD44 induces glycolysis-to-oxidative phosphorylation transition via modulation of the c-Src–Akt–LKB1–AMPKα pathway. Biochem. J. 473, 3013–3030 (2016). 27458252 10.1042/BCJ20160613

[43] K. Ayyavoo, P. Velusamy, Pyrene based materials as fluorescent probes in chemical and biological fields. New J. Chem. 45, 10997–11017 (2021).

[44] G. Zandomeneghi, M. R. Krebs, M. G. McCammon, M. Fändrich, FTIR reveals structural differences between native β-sheet proteins and amyloid fibrils. Protein Sci. 13, 3314–3321 (2004). 15537750 10.1110/ps.041024904 PMC2287307

[45] S. A. Hudson, H. Ecroyd, T. W. Kee, J. A. Carver, The thioflavin T fluorescence assay for amyloid fibril detection can be biased by the presence of exogenous compounds. FEBS J. 276, 5960–5972 (2009). 19754881 10.1111/j.1742-4658.2009.07307.x

[46] N. V. Jafari, J. L. Rohn, The urothelium: A multi-faceted barrier against a harsh environment. Mucosal Immunol. 15, 1127–1142 (2022). 36180582 10.1038/s41385-022-00565-0 PMC9705259

[47] P. Zhang, G. Wu, D. Zhang, W.-F. Lai, Mechanisms and strategies to enhance penetration during intravesical drug therapy for bladder cancer. J. Control. Release 354, 69–79 (2023). 36603810 10.1016/j.jconrel.2023.01.001

[48] J. Idiago-López, E. Moreno-Antolín, M. Eceiza, J. M. Aizpurua, V. Grazú, J. M. de la Fuente, R. M. Fratila, From bench to cell: A roadmap for assessing the bioorthogonal “click” reactivity of magnetic nanoparticles for cell surface engineering. Bioconjug. Chem. 33, 1620–1633 (2022). 35857350 10.1021/acs.bioconjchem.2c00230 PMC9501912

[49] E. Milotti, R. Chignola, Emergent properties of tumor microenvironment in a real-life model of multicell tumor spheroids. PLOS ONE 5, e13942 (2010). 21152429 10.1371/journal.pone.0013942 PMC2994713

[50] P. Das, T. Sercu, K. Wadhawan, I. Padhi, S. Gehrmann, F. Cipcigan, V. Chenthamarakshan, H. Strobelt, C. dos Santos, P.-Y. Chen, Y. Y. Yang, J. P. K. Tan, J. Hedrick, J. Crain, A. Mojsilovic, Accelerated antimicrobial discovery via deep generative models and molecular dynamics simulations. Nat. Biomed. Eng. 5, 613–623 (2021). 33707779 10.1038/s41551-021-00689-x

[51] M. Patra, K. Zarschler, H.-J. Pietzsch, H. Stephan, G. Gasser, New insights into the pretargeting approach to image and treat tumours. Chem. Soc. Rev. 45, 6415–6431 (2016). 27722526 10.1039/c5cs00784d

[52] A. L. Efros, D. J. Nesbitt, Origin and control of blinking in quantum dots. Nat. Nanotechnol. 11, 661–671 (2016). 27485584 10.1038/nnano.2016.140

[53] X. Robin, N. Turck, A. Hainard, N. Tiberti, F. Lisacek, J.-C. Sanchez, M. Müller, pROC: An open-source package for R and S+ to analyze and compare ROC curves. BMC Bioinformatics 12, 77 (2011). 21414208 10.1186/1471-2105-12-77 PMC3068975

[54] O. A. Gederaas, A. Johnsson, K. Berg, R. Manandhar, C. Shrestha, D. Skåre, I. K. Ekroll, A. Høgset, A. Hjelde, Photochemical internalization in bladder cancer–development of an orthotopic in vivo model. Photochem. Photobiol. Sci. 16, 1664–1676 (2017). 28972608 10.1039/c7pp00176b

[55] O. Sanli, J. Dobruch, M. A. Knowles, M. Burger, M. Alemozaffar, M. E. Nielsen, Y. Lotan, Bladder cancer. Nat. Rev. Dis. Primers 3, 17022 (2017). 28406148 10.1038/nrdp.2017.22

[56] F. C. de Jong, T. D. Laajala, R. F. Hoedemaeker, K. R. Jordan, A. C. J. van der Made, E. R. Boevé, D. K. E. van der Schoot, B. Nieuwkamer, E. A. M. Janssen, T. Mahmoudi, J. L. Boormans, D. Theodorescu, J. C. Costello, T. C. M. Zuiverloon, Non–muscle-invasive bladder cancer molecular subtypes predict differential response to intravesical Bacillus Calmette-Guérin. Sci. Transl. Med. 15, eabn4118 (2023). 37224225 10.1126/scitranslmed.abn4118 PMC10572776

[57] V. Cuccurullo, G. D. Di Stasio, F. Manti, P. Arcuri, R. Damiano, G. L. Cascini, The role of molecular imaging in a muscle-invasive bladder cancer patient: A narrative review in the era of multimodality treatment. Diagnostics 11, 863 (2021). 34064755 10.3390/diagnostics11050863 PMC8151158

[58] J.-W. Bai, S.-Q. Qiu, G.-J. Zhang, Molecular and functional imaging in cancer-targeted therapy: Current applications and future directions. Signal Transduct. Target. Ther. 8, 89 (2023). 36849435 10.1038/s41392-023-01366-y PMC9971190

