# Supplementary material for: Assembly-formed bioorthogonal chimeric artificial receptors enable high-contrast fluorescence imaging of tumors
Source: Sci Adv. 2026 Aug 5;12(32):eaed9403. doi: 10.1126/sciadv.aed9403 (PMC13440426; doi:10.1126/sciadv.aed9403)
Supplement: Supplementary file 1 [file sciadv.aed9403_sm.pdf]

Science Advances
AAAS

Supplementary Materials for

Assembly-formed bioorthogonal chimeric artificial receptors enable high-contrast fluorescence imaging of tumors

Jianli Zuo *et al.*

Corresponding author: Yongjun Dang, yjdang@cqmu.edu.cn; Lijuan Bai, bailj1018@cqmu.edu.cn; Hongwen Liang, lianghongwen@cqmu.edu.cn

Sci. Adv. **12**, eaed9403 (2026)
DOI: 10.1126/sciadv.aed9403

This PDF file includes:

Supplementary Materials and Methods
Figs. S1 to S59
Tables S1 to S4

Supplementary materials and methods

Materials

The RNAlater™ RNA stabilization solution and 4',6-diamidino-2-phenylindole (DAPI) staining solution were obtained from Shanghai Beyotime Biotechnology Co., Ltd. The anti-human CD44var (v6) (clone VFF-18, BMS125) antibody and MMP2 protein were purchased from eBioscience and MedChemExpress LLC (USA), respectively. Piperidine and phenol were obtained from Chengdu Kelong Chemical Co., Ltd. Trifluoroacetic acid, 4-methylmorpholine, pyrene, hexafluoroisopropanol, dimethyl sulfoxide (DMSO), rhodamine B (RhB) were both purchased from Shanghai Macklin Biochemical Technology Co., Ltd. L-Ascorbic acid, DBCO-acid, Fmoc-NH-PEG$_4$-CH$_2$CH$_2$COOH, Cyanine5 azide chloride (azide-Cy5) and Cy5-SE were obtained from Shanghai Bide Pharmatech Co., Ltd. 3-azido-7-hydroxycoumarin (coumarin) and D-Luciferinpotassium were obtained from Beijing Bailingwei Technology Co., Ltd. and Dalian Meilun Biotechnology Co., Ltd., respectively. Carprofen was supplied by Shanghai Aladdin Biochemical Technology Co., Ltd. The clinical bladder cancer specimens used in this study were primarily obtained from patients undergoing surgical resection at a single center. Inclusion criteria were: a confirmed diagnosis of non–muscle-invasive bladder cancer (NMIBC), no prior chemotherapy or radiotherapy, and intact tumor tissue suitable for imaging analysis. Exclusion criteria included: concurrent other urological malignancies, severe systemic diseases, or incomplete sample preservation.

Methods

Validation of click reaction between DBCO-peptide and azide-Cy5

To validate the click reaction, 3-azido-7-hydroxycoumarin (coumarin) was selected as the model molecule. The time-dependent fluorescence spectra of DBCO-RSP after reaction with coumarin at wavelengths between 400 and 550 nm were monitored using an FS50 fluorescence spectrophotometer. Briefly, DBCO-RSP (5 mM) and coumarin (5 mM) were diluted to a final concentration of 50 μM in H_2O/DMSO (95:5 v/v) solution. Equal volumes of these two solutions were mixed and reacted for different times, after which the resulting fluorescence values were determined using a fluorescence spectrophotometer (λex = 380 nm, λem = 468 nm). The slit width of both monochromators was set to 9 nm. The reaction rate at concentration-dependent levels was evaluated using the same method. In addition, DBCO-RSP was co-incubated sequentially with ThT and azide-Cy5 for 30 minutes to observe fluorescence colocalization and validate the click reaction further.

Cell culture

The RT112, EJ, Human Umbilical Vein Endothelial Cells (HUVECs) and MDA-MB-231 cell were both cultured in Dulbecco’s Modified Eagle Media (DMEM) supplemented with FBS (10%), streptomycin and penicillin (1%). The T24 cells and mesenchymal stem cells (MSCs) were cultured in Roswell Park Memorial Institute (RPMI) 1640 and α-MEM supplemented with FBS (10%), streptomycin and penicillin (1%), respectively.

Bioinformatics analysis of CD44

A gene expression matrix of tumor cell lines was obtained from the CCLE dataset (https://depmap.org/portal/data_page/?tab=allData) to analyze the expression profile of CD44 in bladder cancer. Statistical analyses were performed using R software (version 4.0.3), and the results were considered statistically significant when the p-value was less than 0.05.

Synthesis of peptides

The peptide was be synthesized using standard solid-phase peptide synthesis (SPPS) techniques based on Fmoc-coupling chemistry. Firstly, the Fmoc-Asp(otbu)-wang resin (0.1 mmol) was dissolved in N, N-dimethylformamide (DMF) to swell the resin. The amino group was revealed by treatment with a deprotection agent, and the success of the deprotection was confirmed using Kaiser's reagent, which consists of ninhydrin, phenol and L-ascorbic acid dissolved in anhydrous ethanol. Next, the Fmoc-protected amino acid was dissolved in a peptide coupling solution containing HATU (1 mmol) and HOBT (1 mmol), which was added to the deprotected solution above to initiate an amide coupling reaction. The success of the amino acid coupling reaction was confirmed using Kaiser's reagent. This process was then repeated with sequentially coupled to PEG_4 (0.5 mmol) and DBCO (0.5 mmol), after which the product was assayed with Kaiser reagent. Finally, the peptide undergoes methanol shrinkage, after which the Wang resin was removed using a cleavage solution consisting of ultrapure water (2.5%), triisopropylsilane (2.5%), 1,2-ethanedithiol (2.5%) and trifluoroacetic acid (92.5%). Following centrifugation with cold ethyl ether and subsequent drying, the crude peptide was purified using high performance liquid chromatography (HPLC), yielding the pure peptide. The molecular weights of the pure peptides were verified using electrospray Ionization mass spectrometry (ESI-MS).

HPLC analysis

DBCO-TPP (800 μM), DBCO-RSP (800 μM), as well as DBCO-TPP (800 μM) co-incubated with an MMP2 (10 μg mL^{-1}) solution were each taken in 350 μL aliquots. Subsequently, 150 μL of acetonitrile was added to each sample during sonication, causing the solution to become clear immediately. The mixture was centrifuged at 10,000 rpm for 10 min, then filtered through a 0.22 μm membrane filter into a brown liquid-phase vial. Finally, the reversed-phase HPLC system with a Waters Symmetry C18 column (5 μm, 100Å) was used to collect the absorption peaks at the wavelength of 220 nm.

Fourier transform infrared spectroscopy (FTIR) analysis

Infrared (IR) spectroscopy was employed to characterize the formation of β-sheet structure. Peptide solutions at defined concentrations were deposited onto FTIR pellets, dried at 40 °C, and analyzed by scanning in the range of 1800-1400 cm^{-1} using an infrared spectrometer (Nicolet iS10, Thermo Scientific). Blank KBr was used as the control.

Scanning electron microscope (SEM) and Transmission electron microscope (TEM) characterization

SEM and TEM were used to characterize the morphology of peptides and the morphological changes that occurred after the co-incubation of DBCO-TPP and the MMP2 enzyme. Briefly, DBCO-TPP (100 μM), DBCO-RSP (30 μM), and DBCO-TPP (100 μM) pretreated with MMP2 enzyme (15 μg mL^{-1}) for 2 hours were dropped onto glass slides and air-dried. The dried samples were then sputter-coated with gold and characterized using SEM (ProX, Phenom). Subsequently, DBCO-TPP (50 μM) pretreated with MMP2 (2 μg mL^{-1}) solution (15 μL) was applied to copper grids and allowed to adsorb for 5 minutes and stained with uranyl acetate (15 μL) at room temperature for 5 minutes, followed by gentle rinsing with deionized water and drying under a nitrogen stream. TEM (Ht-7700, HITACHI) was employed to observe the morphological changes of the peptides.

Critical aggregation concentration (CAC) characterization

The pyrene dye was used as a fluorescent probe to determine CAC and explore the self-assembly ability of the peptides. The DBCO-TPP and DBCO-RSP were added to a pyrene solution (2.4 mg mL^{-1}), which was then diluted to different concentrations. After being incubated in the dark for 1 hour, the samples were analyzed using a fluorescence spectrophotometer (FS50, Edinburgh Instruments). The fluorescence values at 384 and 373 nm were recorded and plotted on graphs, with the horizontal axis representing the peptide concentration and the vertical axis representing the fluorescence ratio of the two wavelengths (I_{384}/I_{373}). The fluorescence spectrophotometer was operated with the following parameters: an excitation wavelength of 330 nm and an emission wavelength ranging from 360 to 450 nm. The excitation and emission slit widths were set to 10 and 2 nm, respectively.

Thioflavin T (ThT) assay

ThT, a benzothiazole-based fluorescent dye, was used to verify the secondary structure of the nanofibers formed through MMP2-triggered self-assembly and to analyze the kinetics of this process. Firstly, DBCO-RSP (50 μM) was allowed to self-assemble in ultrapure water for varying durations, followed by incubation with ThT (30 μM) for 1 hour. The self-assembly behavior of DBCO-RSP was evaluated using a fluorescence spectrophotometer and confocal laser scanning microscope (CLSM). Similarly, DBCO-TPP pretreated with MMP2 was incubated with ThT (30 μM), after which the resulting self-assembly behavior following enzymatic cleavage was evaluated using a fluorescence spectrophotometer (λ_{ex} = 450 nm), CLSM and a microplate reader. In addition, a high-content imaging system (HCS) was used to monitor the fluorescence intensity at various time points (0.25, 1, 2, 3, 4, 5, 6, 7, 8, 9, 10 and 11 hours) to assess the kinetics of the self-assembly reaction.

Photobleaching assay in vitro

To evaluate the photostability of the samples, photobleaching experiments were performed in solution. The three groups of samples were: G1 (azide-Cy5, 10 μM), G2 (DBCO-TPP (500 μM) + azide-Cy5 (10 μM)), and G3 (DBCO-TPP (500 μM) + MMP2 (10 g mL^{-1}) + azide-Cy5 (10 μM)). Each sample (200 μL) was placed in a 96-well plate and irradiated with a light source (excitation wavelength 660 nm, power 0.0920 W, exposure time 1800 s, illuminated area 0.32 cm^2). The total light dose was calculated using the formula:

Light dose (J cm^{-2}) = Power (W) × Exposure time (s) / Illuminated area (cm^2).

After irradiation, fluorescence intensity was recorded, with each group measured in at least three independent replicates. Fluorescence intensities were background-corrected and normalized to the total light dose. Finally, light dose–dependent photobleaching curves were plotted to quantitatively compare the fluorescence decay of each group under the same light dose. The photobleaching curves and fluorescence decay as a function of light dose were fitted using the [Agonist] vs. Response – Variable slope (four parameters) model to accurately describe the photobleaching kinetics of each sample.

Cell Viability Assays

HUVECs, MSCs, EJ cells, RT112 cells, DC 2.4 cells and RAW 264.7 cells were seeded at a density of 1×10^3 cells per well in 96-well plates. The plates were then incubated in a CO_2 incubator for 24 hours to allow the cells to attach and grow, while cell confluency reached approximately 80%, the experiment was initiated. DBCO-TPP (20 mM) and DBCO-RSP (20

mM) were serially diluted in DMEM medium to obtain a range of concentrations (0, 0.39, 0.78, 1.56, 3.125, 6.25, 12.5, 25, 50 and 100 μM). The cells were then treated with the respective dilutions and incubated for a further 48 hours in a CO_2 incubator. After incubation, the culture medium was discarded and the cells were washed three times with PBS. Then, 100 μL of CCK-8 reagent (culture medium: CCK-8 = 9:1, v/v) was added to each well. The optical density (OD) was measured at 450 nm using a multifunctional microplate reader and cell viability was calculated accordingly.

Validation of click reaction between DBCO-peptide and azide-Cy5

To validate the click reaction, 3-azido-7-hydroxycoumarin (coumarin) was selected as the model molecule. The time-dependent fluorescence spectra of DBCO-RSP after reaction with coumarin at wavelengths between 400 and 550 nm were monitored using an FS50 fluorescence spectrophotometer. Briefly, DBCO-RSP (5 mM) and coumarin (5 mM) were diluted to a final concentration of 50 μM in H_2O/DMSO (95:5 v/v) solution. Equal volumes of these two solutions were mixed and reacted for different times, after which the resulting fluorescence values were determined using a fluorescence spectrophotometer (λex = 380 nm, λem = 468 nm). The slit width of both monochromators was set to 9 nm. The reaction rate at concentration-dependent levels was evaluated using the same method. In addition, DBCO-RSP was co-incubated sequentially with ThT and azide-Cy5 for 30 minutes to observe fluorescence colocalization and validate the click reaction further.

Investigation of the assembly behavior of DBCO-TPP on cell surface

SEM was used to observe the assembly behavior of DBCO-TPP on CD44v6-high and -low expressing cells. Briefly, RT112, EJ, HUVECs, MSC and MDA-MB-231 cells were seeded at a density of 5×10^4 onto sterile coverslips in 24-well plates. Then, DBCO-TPP (50 μM) was added and incubated for 12 hours, then discard the supernatant, wash three times with PBS and fix with a 2.5% glutaraldehyde solution for another 12 hours. The glutaraldehyde solution was then discarded and the cells were eluted sequentially using a gradient of anhydrous ethanol of different concentrations (25%, 50%, 75%, 95% and 100%) for 5 minutes. This process was repeated three times and the cells were sprayed with gold to observe the morphological changes on the cell surface.

Synthesis of fluorescent peptides

Firstly, the TPP and RSP peptides were synthesized using the SPPS method. Following PEG_4 conjugation, the Fmoc-protected groups were removed by treating with a deprotection reagent for 15 minutes to expose the amine groups, and pure TPP and RSP peptides were obtained according to the standard synthetic procedure. Subsequently, 30 mg of rhodamine B (RhB) was dissolved in an activation buffer solution of 0.1 M MES and 0.5 M NaCl to form a solution of 10 mg mL^{-1}. Then, EDC (120 mg) and NHS (180 mg) were added to the above solution for 15 minutes. Next, the TPP and RSP peptides (both at a concentration of 10 mg mL^{-1}) were added to the solution in equal volumes and mixed for 2 hours. The resulting reaction mixture was dialyzed for 24 hours, followed by lyophilization to yield rhodamine-labeled TPP (RhB-TPP) and rhodamine-labeled RSP (RhB-RSP) fluorescent peptides.

Targeting validation of TPP

EJ cells were seeded in 24-well plates and cultured for 24 hours, followed by incubation with RhB-TPP (50 μM) for 2 hours. The medium was then removed, and the cells were rinsed three times with PBS, fixed with paraformaldehyde for 10 minutes, and stained with DAPI for 5 minutes. Fluorescence colocalization was then observed using CLSM. In addition, the targeting ability of RhB-TPP was investigated in other cell lines (RT112, MSCs and HUVECs) using the same procedure.

Assessment of the penetrating ability

Firstly, EJ cells were seeded at a density of 1×10^4 cells/mL in an ultralow adsorption plate and centrifuged at 1000 ×g for 5 minutes. The culture medium was half-replaced every other day, and the plates were incubated at 37 ℃ for 6 days to form EJ multicellular tumor spheroids (MCTs) with diameters of approximately 300-350 μm. Subsequently, RhB-TPP and RhB-RSP (both at a concentration of 50 μM) were added and incubated with the MCTs for 2 hours. After incubation, the MCTs were washed three times with PBS and transferred to confocal dishes. Finally, CLSM was then used to acquire z-stack images of the MCTs, scanning from the top to the center of the spheroids at 20 μm intervals.

In vitro validation of the AEPA effect

Firstly, DBCO-RSP (4 mM) was diluted with sterile PBS to a final concentration of 400 μM and added to azide-Cy5 (400 μM) for 80 minutes in the dark, and the Cy5-labelled RSP (Cy5-RSP) fluorescent peptide was obtained by filtration through a sterile membrane. Secondly, the G-TP peptide was synthesized using the SPPS method. Following the deprotection reaction, the Fmoc group was removed to expose the amino group. The purified G-TP peptide was obtained following standard protocols. Then, FITC-NHS ester (1 mg) and G-TP (10 mg) were separately dissolved in DMSO (400 μL). FITC-NHS ester and triethylamine (15 μL) were sequentially added to the G-TP solution and stirred in an ice bath for 8 hours in the dark. The reaction mixture was then dialyzed for 48 hours, lyophilized, and purified to obtain the FITC-labeled G-TP peptide (FITC-TP). Finally, EJ cells were seeded in 24-well plates at a density of 5×10^4 cells per well and incubated for 12 hours. Then, the medium was discarded, and the cells were incubated with FITC-TP (2 μM) and PBS for 30 minutes, and then incubated with Cy5-RSP for another 20 minutes. The cells were washed three times with PBS, fixed with paraformaldehyde, and stained with DAPI for 10 minutes. The co-localization of fluorescence on the cell membrane surface was observed using CLSM.

Molecular dynamics simulation

In this study, a pure phospholipid bilayer system was constructed using the Membrane Builder module of the CHARMM-GUI platform. The CD44v6 protein structure was obtained from the AlphaFold database and the MMP2 protein structure was retrieved from the RCSB PDB crystal structure database (PDB ID: 8H78). Firstly, the most stable conformation of the CD44v6 protein in complex with DBCO-TPP was selected. The simulation procedure involved using centroid-based models for extensive conformational sampling, followed by local refinement using all-atom models. The resulting conformations were then ranked based on orientation, position and binding energy. ZDOCK software was then used to select the top 50 complexes with the highest clustering density and binding energy for binding site prediction. Protein pairs that docked at the same site were considered reliable enough for phase analysis. Ultimately, the structure with the

largest cluster size and the lowest binding energy of -23.70 kcal/mol was selected for MD simulation.

Subsequently, the recognition and binding process between the peptide and the phospholipid bilayer was simulated. The peptide-membrane complex structure was modelled using the AMBER software suite, employing the AMBER ff99SB force field, GAFF parameters, and the TIP3P water model. The complex was then energy-optimized using a combination of Langevin dynamics and Monte Carlo methods. The simulation process involved the following steps: first, energy-minimized of the entire system was carried out using the steepest descent algorithm; then, the system was equilibrated using Langevin thermostat control for 100 ps, followed by pressure equilibration for another 100 ps. Next, a 45 ns molecular dynamics simulation was performed under the NPT ensemble with a time step of 2 fs, and long-range electrostatic interactions were computed using the Ewald method. Finally, the simulation trajectory was monitored using the sander module in AMBER, and the resulting structures were visualized using PyMOL.

Bio-TEM characterization of DBCO-TPP co-incubated with MCTs

The EJ MCTs was co-incubated with DBCO-TPP (50 μM) and culture medium for 24 hours, respectively. After incubation, the MCTs were washed three times with PBS and centrifuged at 1200 rpm for 10 minutes to form larger clumps of cells. These clumps were then fixed with glutaraldehyde (4%) at 4 ℃ for 12 hours, after which they were observed for the formation of nanofibers using standard TEM operating procedures.

Determination of Detection Limit (LOD) in EJ Cells Using DBCO-TPP and Azide-Cy5

EJ cells were plated at different densities in 96-well plates. After cell adhesion, DBCO-TPP (100 μM) was added and incubated for 2 h, followed by a single PBS wash. Azide-Cy5 (0.25 μM) was then added and incubated for 30 min. Fluorescence intensity in EJ cells was measured using a microplate reader (λex = 640 nm, λem = 670 nm) to determine the detection limit (LOD).

Investigation of photostability in the APBC model

To validate the photostability of the PRE-targeted platform, this work simulated a clinical cystoscopy operating environment using 660 nm continuous laser irradiation for between 0 and 6 hours. The BALB/c nude were randomly divided into three groups (G1, G2 and G3). The G1 and G2 groups injected azide-Cy5 solution (10 μM) and TP-Cy5 solution (10 μM) into air-pouch for 30 min, respectively. The G3 group pre-injected DBCO-TPP solution (50 μM) into air-pouch for 2 hours, followed by a PBS perfusion and then an injection of azide-Cy5 solution (10 μM) for 30 minutes. After incubation, all mice were washed with PBS and euthanized, whose tumor tissue samples from the APBC model were irradiated with a 660 nm laser (560 W cm^{-2}) at various time intervals to deliver specific energy densities of 0.6, 34, 67, 101, 134, 168 and 202 kJ cm^{-2}. The samples were then placed on a black background board and subjected to fluorescence imaging using an IVIS system (λ_{ex} = 605 nm, λ_{em} = 670 nm).

Establishment of xenograft model and fluorescence imaging

The establishment of the xenograft model was performed using EJ cells in the logarithmic growth phase. First, a suspension containing 5×10^6 EJ cells in 100 μL medium was subcutaneously injected into the right hind leg of the female BALB/c nude mice (6-8 weeks, 18-20 g). The tumor volume was measured at two-day intervals for two weeks to monitor growth progression, after which in vivo imaging analysis could begin. The experimental group received

an intratumoral injection of 50 μL of DBCO-TPP (100 μM), followed by an injection of 50 μL of an azide-Cy5 solution (10 μM) after 2 hours interval. The control group received equivalent doses of the azide-Cy5 solution alone. Thirty minutes after injection, whole-body fluorescence imaging analysis was performed using an IVIS system to observe fluorescence retention in vivo.

Bio-TEM characterization of xenograft model

When the tumor volume reached approximately 50 mm^3 [V = (length × width2)/2], 50 μL of DBCO-TPP and PBS were intratumoral injection the tumor tissues to observe structural and morphological changes. Two hours after injection, fresh tumor tissues were collected, washed with PBS, and immersed in pre-cooled 2.5% glutaraldehyde fixative at 4 ℃ for 12 hours. Then, the tumor tissues were rinsed three times with PBS and fixed with 1% osmium dichromate for 2 hours. Gradient dehydration was carried out using ethanol (30%, 50%, 70% and 90%) and acetone (100%) for 20 minutes each time. The dehydrated samples were infiltrated with a mixture of acetone and embedding resin and polymerized at 70 °C for 12 hours. Ultrathin sections of the tumor tissue were prepared and stained with uranyl acetate for 8-15 minutes, followed by counterstaining with lead citrate for 5-10 minutes. Morphological alterations in the tumor tissue were subsequently examined using TEM.

Establishment of APBC model and fluorescence imaging

The APBC model vividly simulates the anatomical structure of the bladder. Female BALB/c nude mice were anaesthetized by inhaling isoflurane and injected subcutaneously with 3 mL of sterile, filtered air to create an air pouch (2.5 × 2 cm). To maintain the pouch's size, an additional sterile air (1 mL) was injected every other day. After seven days, 5×10^6 EJ cells suspended in matrigel (50 μL) were inoculated onto the inner wall of the air pouch (100 μL per mice). Tumor volume was measured every two days, and air was released from the pouch prior to measurement. Imaging analysis commenced once the tumor volume reached approximately 100 mm^3; the mice were randomly divided into three groups (G1, G2 and G3). The G3 group injected DBCO-TPP solution (50 μM) into air-pouch, followed by a PBS perfusion after 2 hours and then an injection of azide-Cy5 solution (10 μM) for 30 minutes. The G2 and G1 groups injected TP-Cy5 solution (40 μM) and azide-Cy5 solution (10 μM) into air-pouch, respectively, for 30 minutes. Following PBS perfusion, all mice were euthanized and tumor tissues from the APBC model were obtained and placed on a black background for fluorescence imaging using an IVIS system (imaging parameters: excitation wavelength 605 nm, emission wavelength 670 nm, exposure time 1 second).

Verification of the detection sensitivity of the APBC Model

To evaluate the recognition capability of the PRE-targeted platform for tumors of varying sizes, APBC models with tumor volumes ranging from 30 ± 1.5 mm^3 to 110 ± 2.3 mm^3 were established. DBCO-TPP solution (50 μM) was injected into the air pouch and incubated for 2 hours. Following PBS perfusion, azide-Cy5 solution (10 μM) was administered and allowed to react for 30 minutes. After PBS wash, the mice were placed on a black background, and fluorescence signals were acquired using an IVIS imaging system.

Preparation of luciferase-transfected T24 (Luc-T24) cells

T24 cells were seeded at a density of 3×10^4 cells per well in 24-well plates and cultured for 24 hours. Subsequently, 20 μL of HitransG P lentiviral transduction enhancement solution and 2 μL

of GV260 lentiviral solution (Shanghai GeneChem Co., LTD.) were added to the culture medium sequentially. After screening the positive cells with puromycin (2 μg mL^{-1}), the concentration was reduced to 0.5 μg mL^{-1} after culturing for 96 h. Seven days later, transduction efficiency was assessed using an IVIS imaging system, and a stable luciferase-expressing T24 cell line (T24-Luc) was successfully established.

Validation of BCAR on the surface of tumor cell membranes

EJ cells were seeded at a density of 2×10^3 cells per well in an ultralow adsorption plate and centrifuged at 1000 ×g for 5 minutes. The cells were cultured in a half-volume medium every other day for 6 days, after which EJ multicellular tumor spheroids (MCTs) with diameters of approximately 300-350 μm were obtained. Then, the spheroids were then incubated with DBCO-TPP (50 μM) and PBS for 2 hours, followed by three washes with PBS. Next, various concentrations of azide-Cy5 (0.25-10 μM) and TP-Cy5 (0.25-100 μM) were added and incubated for 1 hour. Following three further PBS washes, the spheroids were transferred to confocal dishes and examined using confocal laser scanning microscopy (CLSM).

Establishment of orthotopic bladder cancer and fluorescence imaging

An orthotopic bladder cancer model was created using a surgical grafting technique. The pre-emptive analgesic carprofen (0.5 mg mL^{-1}) was injected into BALB/c nude mice 1 hour before surgery. Firstly, BALB/c nude mice were anaesthetized and placed in a supine position. The abdominal area was disinfected, after which a 1 cm midline incision was made to expose the abdominopelvic region by carefully separating the skin and muscle layers. The bladder, located in the hypogastric region, was gently exteriorized using pointed forceps and the intravesical urine was aspirated with an insulin syringe to facilitate cell implantation. Then, a 100 μL suspension of T24-Luc cells was slowly injected into the bladder lumen. The bladder was repositioned and the abdominal wall closed using standard surgical suturing techniques. The mice were observed postoperatively and were not given water for 2 hours to reduce urination and promote maximal interaction between the cancer cells and the bladder mucosa. Approximately 10 days after implantation, 200 μL of D-luciferin potassium salt solution (15 mg mL^{-1}) was administered intraperitoneally and bioluminescent imaging of the bladder cancer was performed on IVIS imaging system to assess tumor establishment and progression. Finally, the mice with orthotopic bladder cancer were randomly divided into three groups (G1, G2 and G3), azide-Cy5 (G1), TP-Cy5 (G2) and DBCO-TPP + azide-Cy5 (G3) were injected respectively to observe the fluorescence imaging.

Biosafety evaluation

Healthy female BALB/c nude mice (6-8 weeks old) were randomly divided into DBCO-TPP and PBS groups ($n = 4$). Three days after injection of DBCO-TPP (50 μM) and PBS, whole blood and serum samples were collected for analysis of blood routine, liver and kidney function, and protein biomarkers.

Supplementary Figures

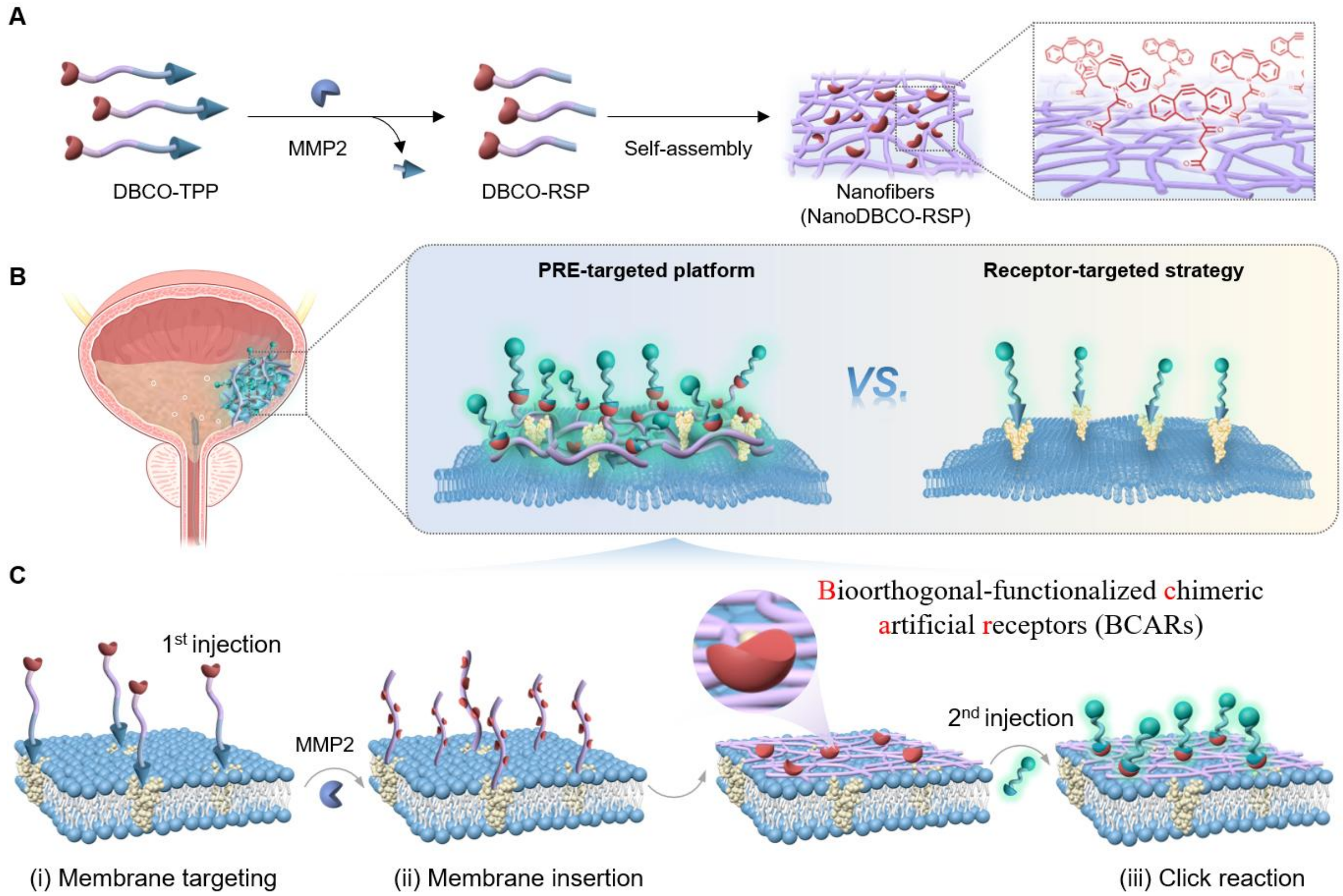

Fig. S1. Schematic illustrates the general idea of this study. (A) DBCO-TPP is cleaved by MMP2 to generate DBCO-RSP, which subsequently undergoes rapid self-assembly into nanofibers (nanoDBCO-RSP) enriched with bioorthogonal DBCO moieties. (**B)** Compared with conventional receptor-targeted strategies, the PRE-targeted strategy constructs artificial receptors on the surface of tumor cells, significantly increasing the number of targeting sites and thereby effectively enhancing imaging performance. (**C)** Mechanism of action for BCAR construction on the surface of tumor cells.

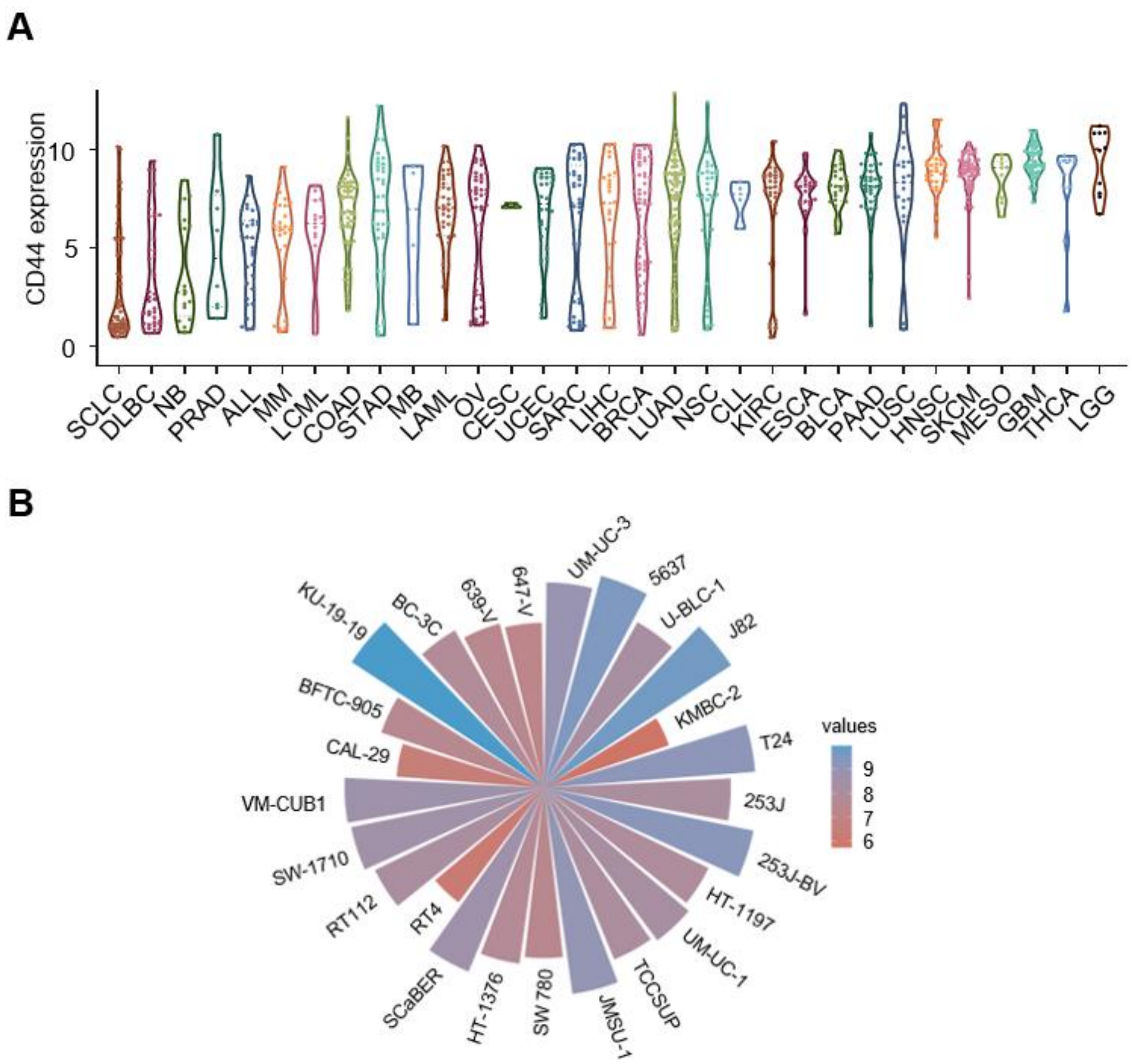

Fig. S2. CD44 expression across various tumor types and bladder cancer cell lines. (**A)** CD44 mRNA expression levels across cancer cell lines from the Cancer Cell Line Encyclopedia (CCLE) (https://www.broadinstitute.org/ccle), with the horizontal axis representing different tumor cell lines and the vertical axis indicating the expression distribution of the gene. (**B)** Expression levels of CD44 in bladder cancer cell lines from the CCLE database.

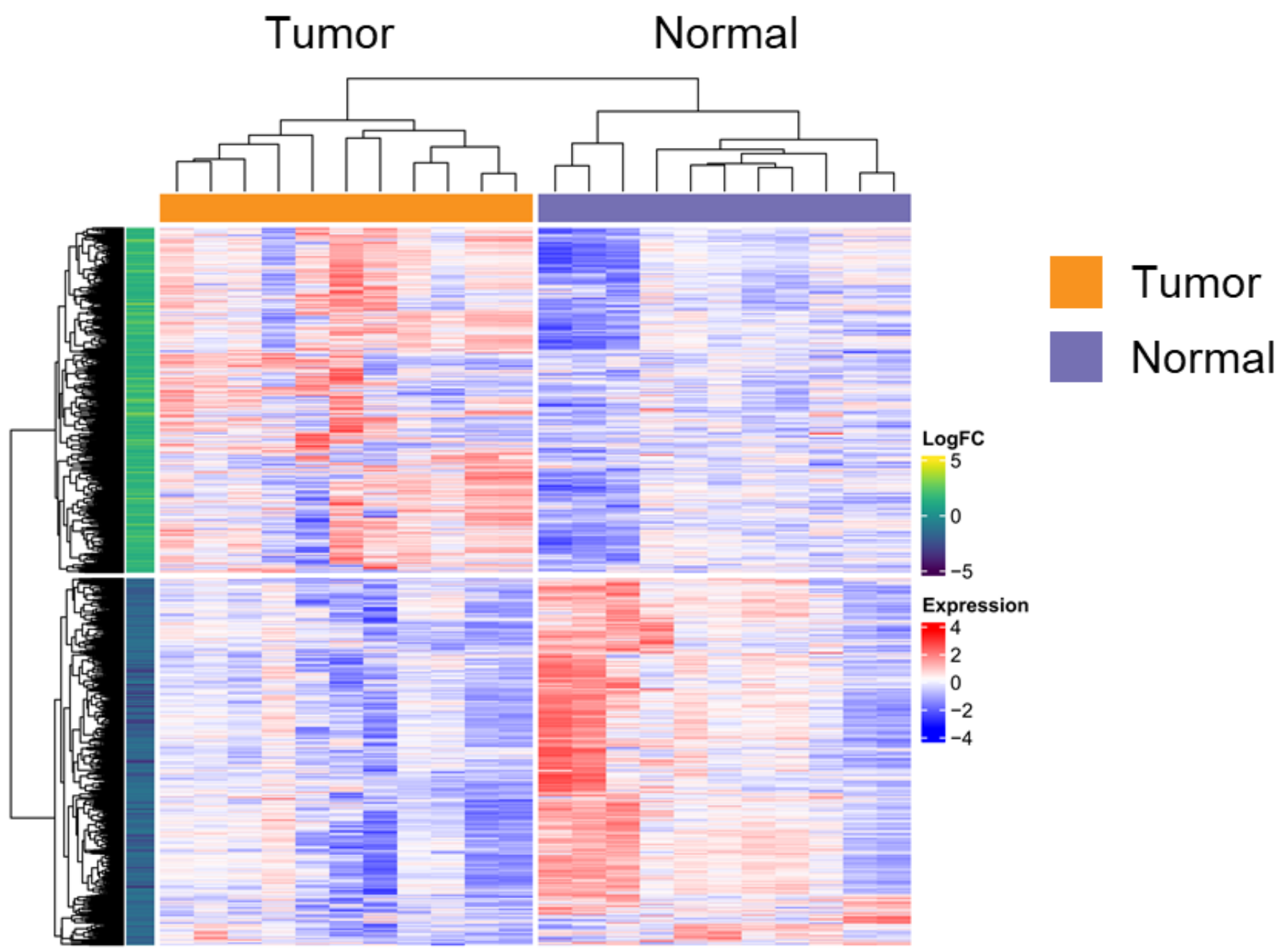

Fig. S3. Heatmap of differentially expressed genes (DEGs) between tumor and normal tissues. The expression matrix was normalized using z-score transformation of TMM values and subjected to hierarchical clustering. The horizontal axis represents samples and the vertical axis represents DEGs.

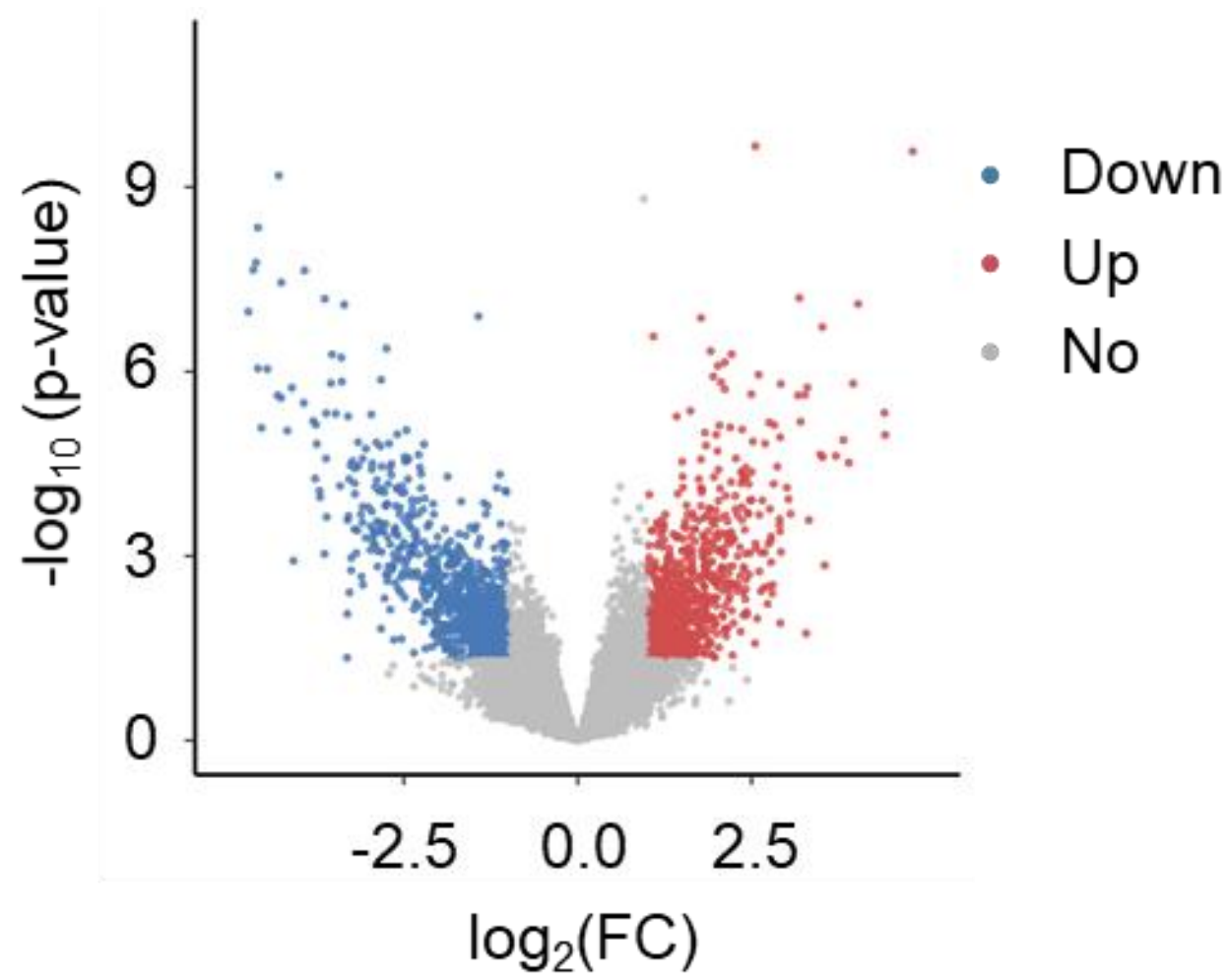

Fig. S4. Volcano plots of DEGs between tumor and normal tissues. The horizontal axis represents the log_2 fold change (log_2FC) and the vertical axis represents the significance level of the difference in expression. Red circle: upregulated genes; Blue circles: downregulated genes; Gray circles: not differentially expressed genes.

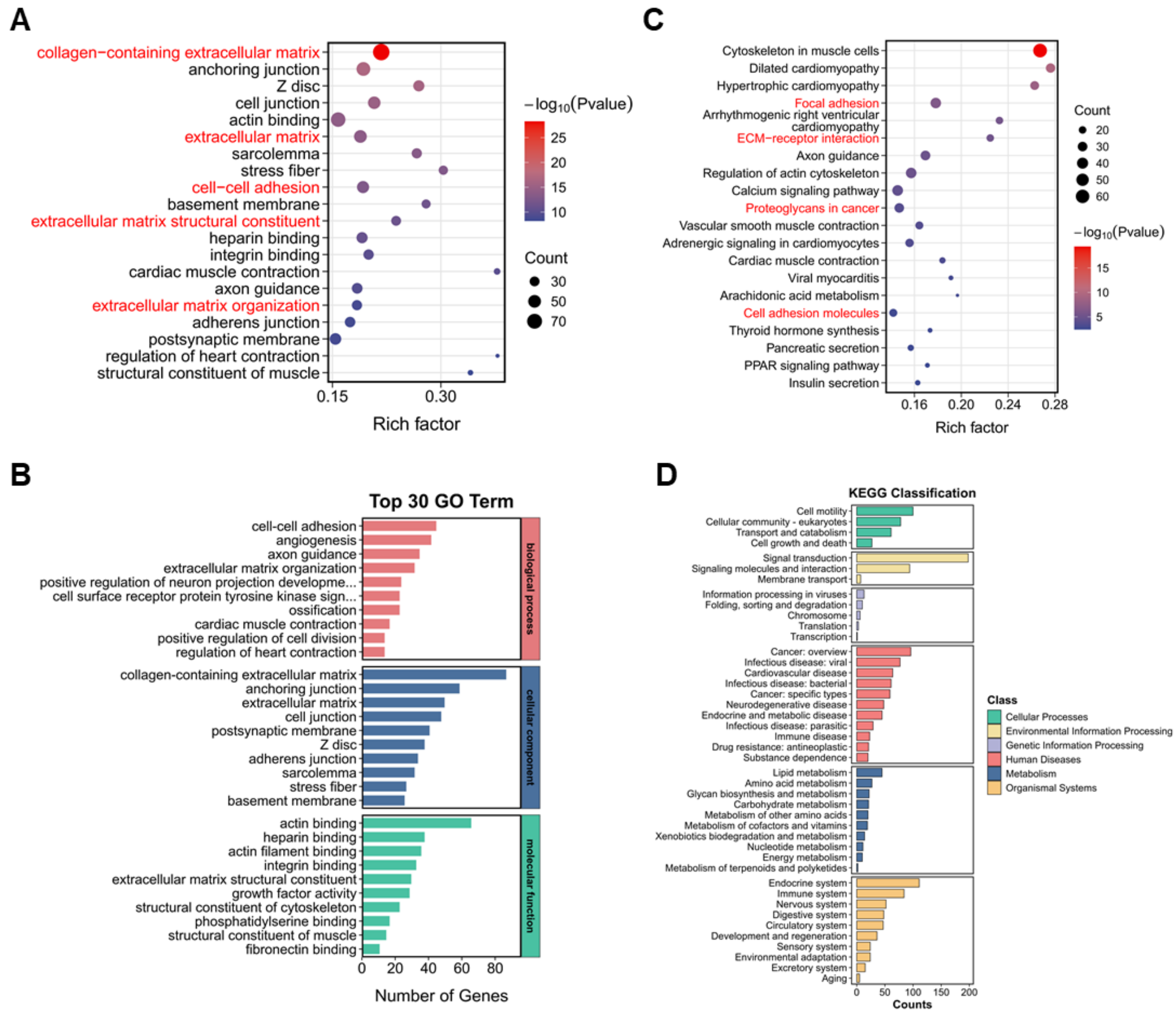

Fig. S5. GO enrichment and KEGG analysis. (A) GO enrichment analysis displaying the top 20 enriched pathways. **(B)** GO enrichment histogram of DEGs. **(C)** KEGG bubble chart showing the top 20 enriched pathways. **(D)** KEGG Level 2 pathway classification of DEGs. The horizontal axis represents the number of DEGs annotated to each KEGG term, while the vertical axis shows KEGG terms at the subcategory level under the major KEGG classifications. Different colors indicate distinct KEGG functional classes.

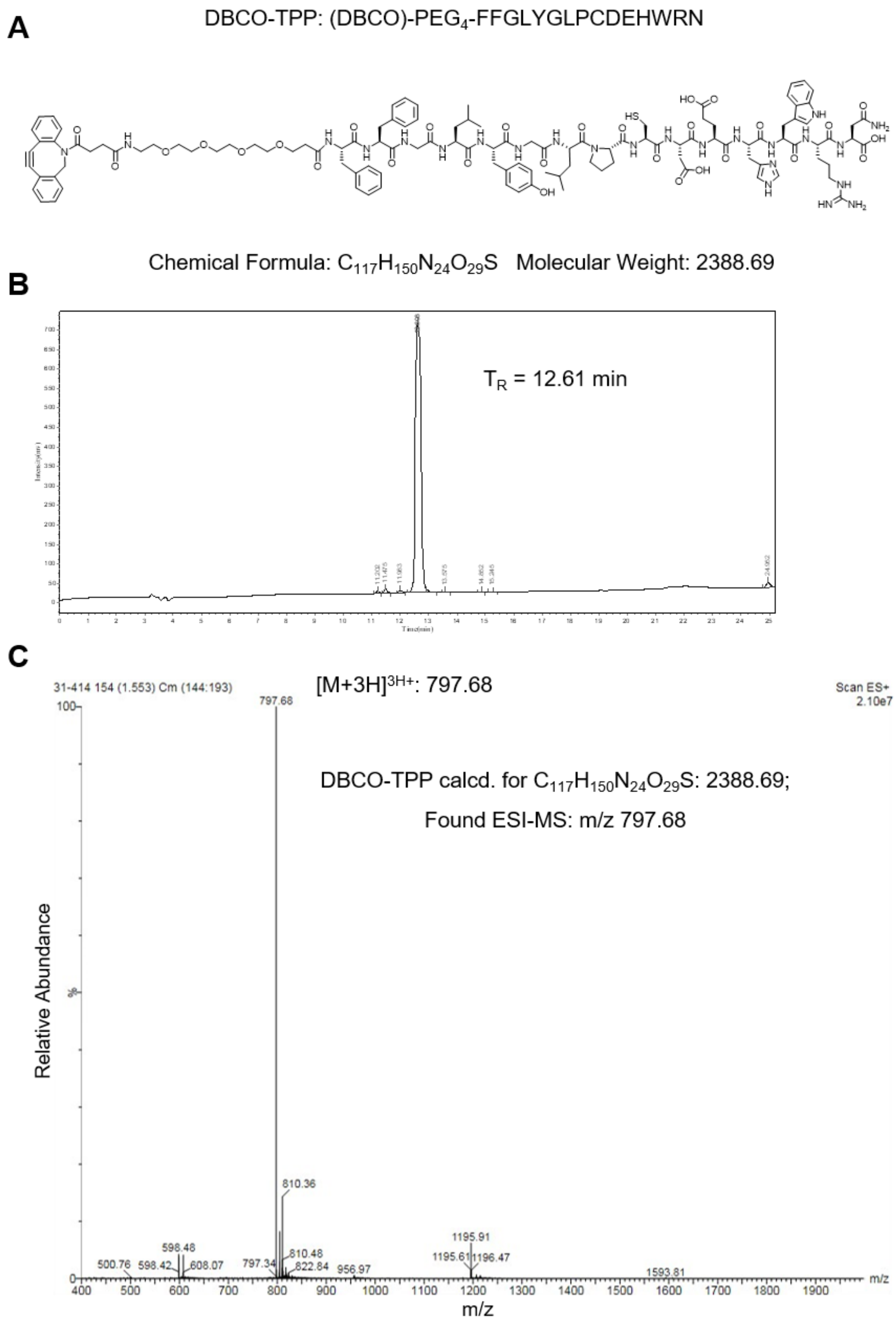

Fig. S6. The chemical structure and characterization of DBCO-TPP. (A) The chemical structure, **(B)** HPLC spectra and **(C)** ESI-MS spectra of DBCO-TPP. The method of HPLC was as follow: the mobile phases consisted of solvent A (0.1% trifluoroacetic acid in 100% acetonitrile) and solvent B (0.1% trifluoroacetic acid in 100% water), with an injection volume of 10 μL.

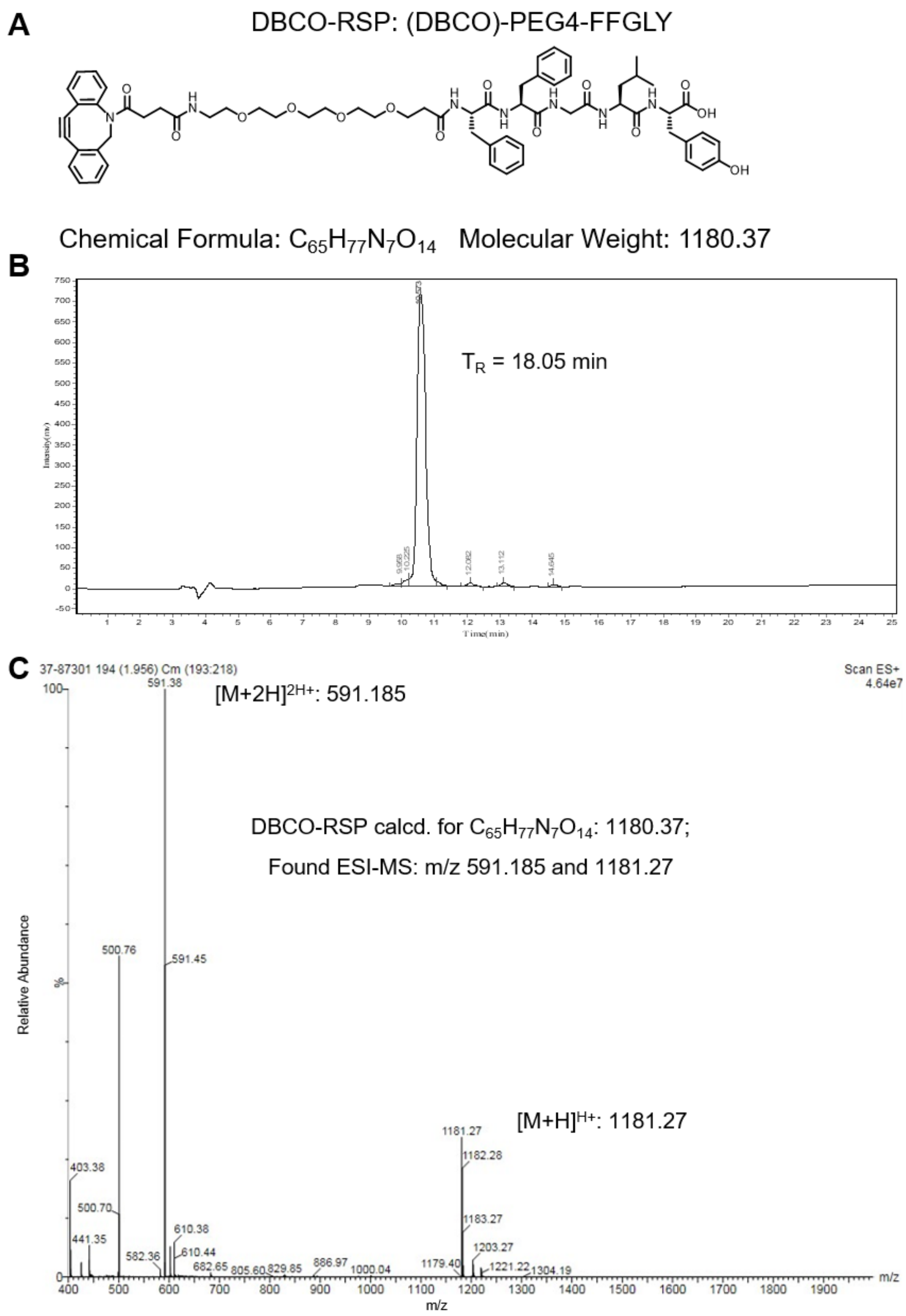

Fig. S7. The chemical structure and characterization of DBCO-RSP. (A) The chemical structure, **(B)** HPLC spectra and **(C)** ESI-MS spectra of DBCO-RSP. The method of HPLC was as follow: the mobile phases consisted of solvent A (0.1% trifluoroacetic acid in 100% acetonitrile) and solvent B (0.1% trifluoroacetic acid in 100% water), with an injection volume of 10 μL.

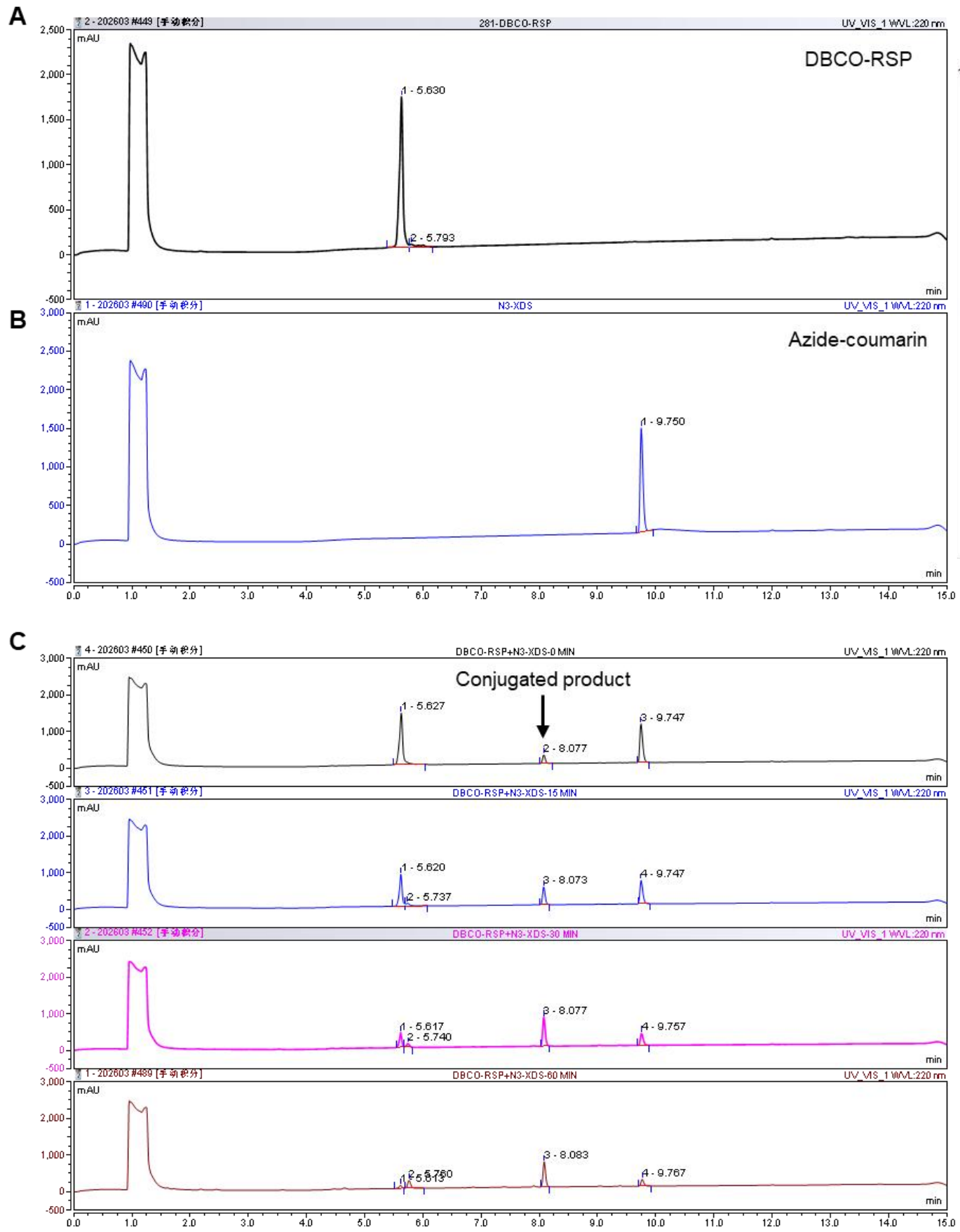

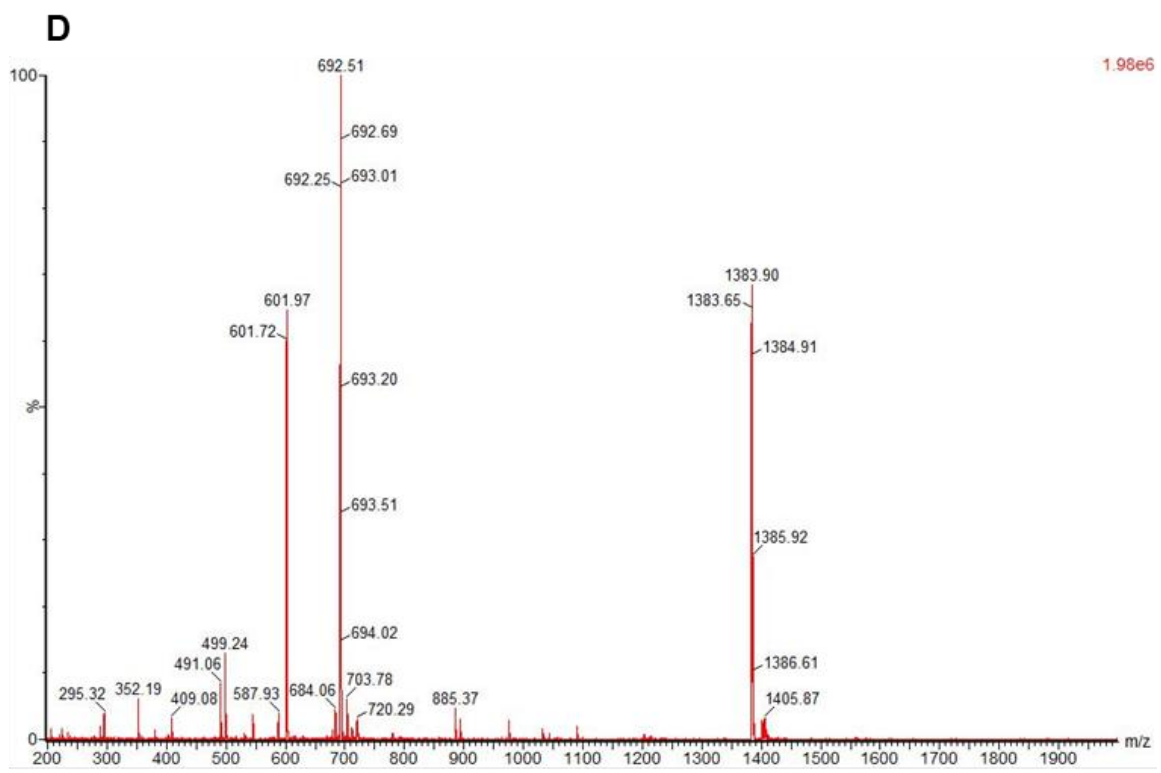

E

Time	DBCO-RSP	Azide–coumarin	Conjugated product
0 min	99.2372	12.6567	61.7765
15 min	47.1048	25.5419	36.8464
30 min	20.7524	40.7732	19.1896
60 min	4.9563	35.5946	10.7925

Fig. S8. HPLC and MS characterization of the reaction between DBCO-RSP and azide-coumarin. (**A**) HPLC chromatogram of DBCO-RSP. (**B**) HPLC chromatogram of azide-coumarin. (**C**) Time-dependent HPLC profiles of the reaction between DBCO-RSP and azide-coumarin. (**D**) Mass spectrum (MS) of the conjugated product. (**E**) Quantification of peak areas corresponding to the species shown in (**C**).

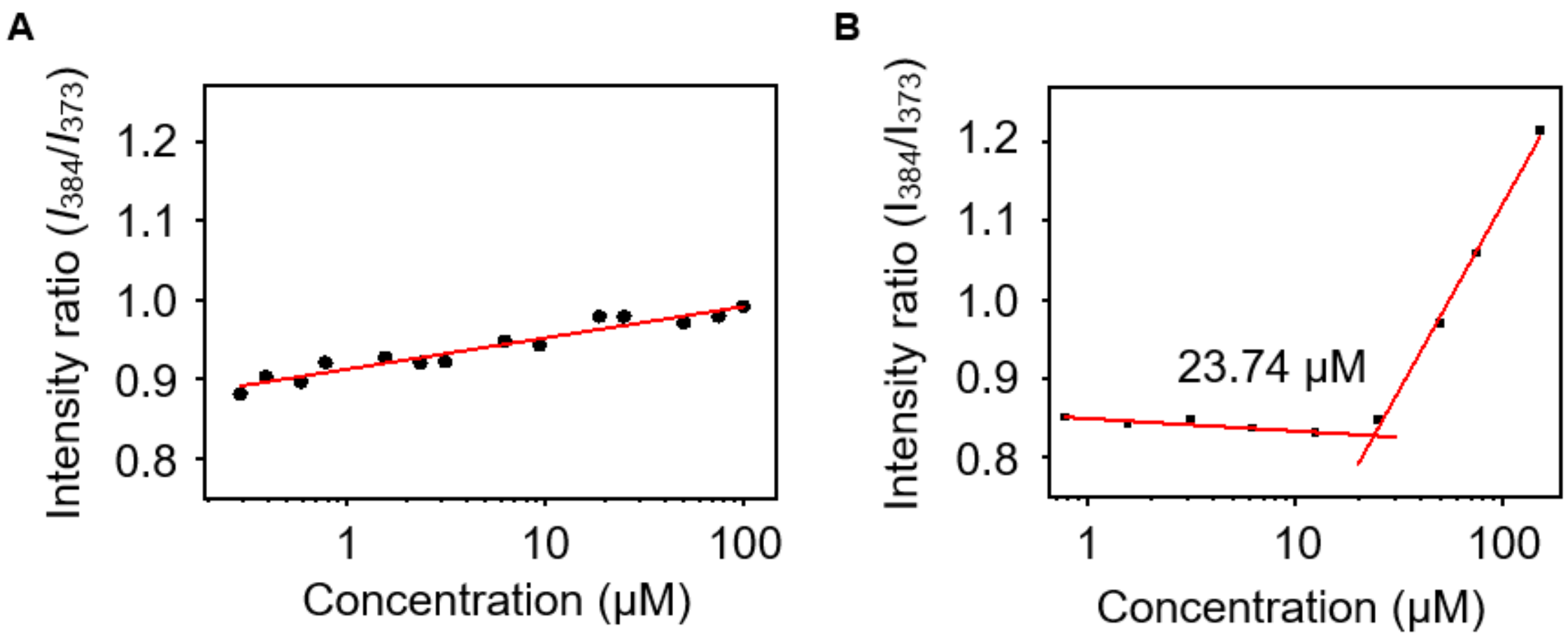

Fig. S9. CAC characterization. CAC of **(A)** DBCO-TPP and **(B)** DBCO-TPP incubated with MMP2. Pyrene as a fluorescence probe.

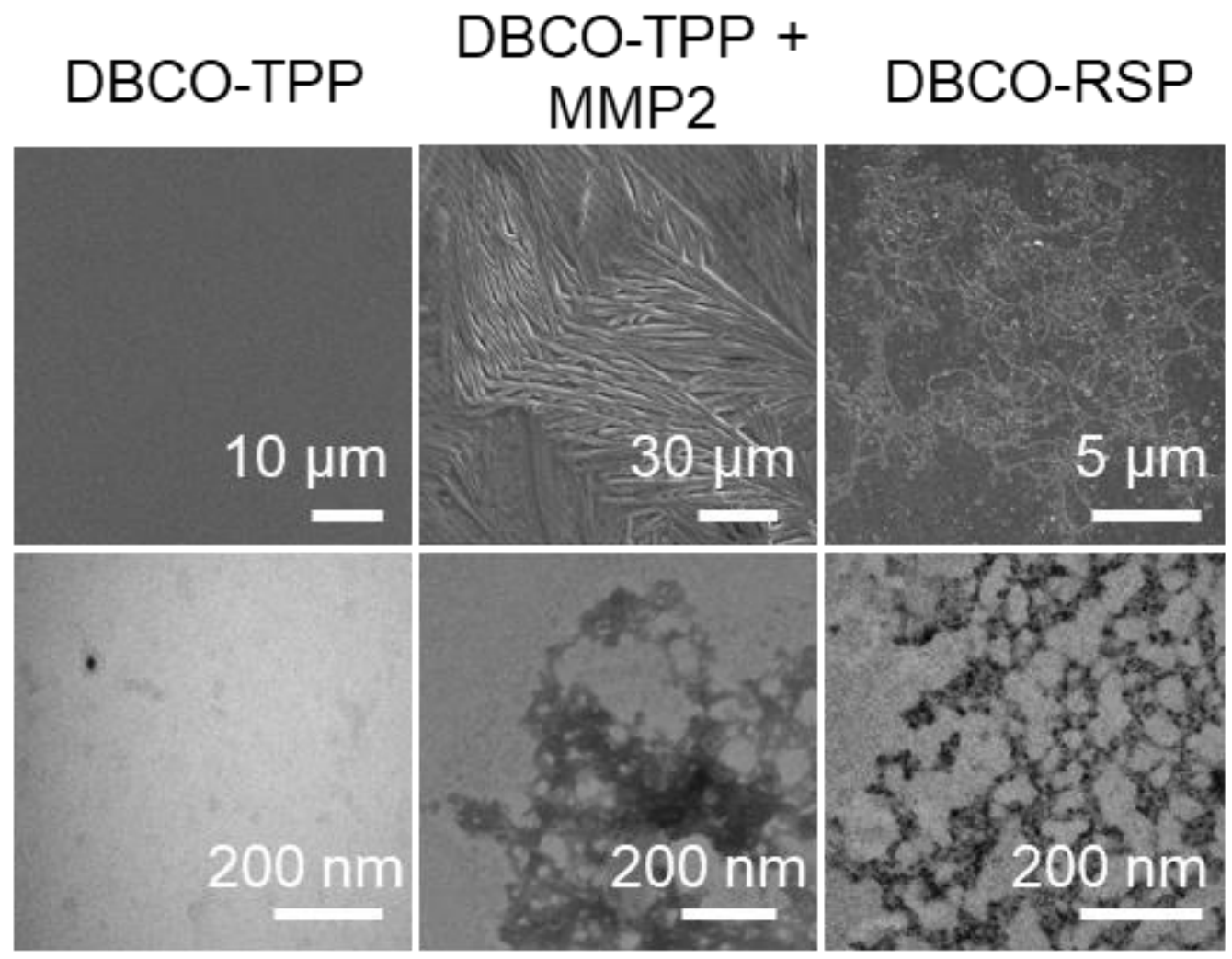

Fig. S10. SEM and TEM characterization. SEM and TEM images of DBCO-TPP (100 μM), DBCO-TPP (100 μM) incubated with MMP2 (15 μg mL^{-1}), DBCO-RSP (30 μM).

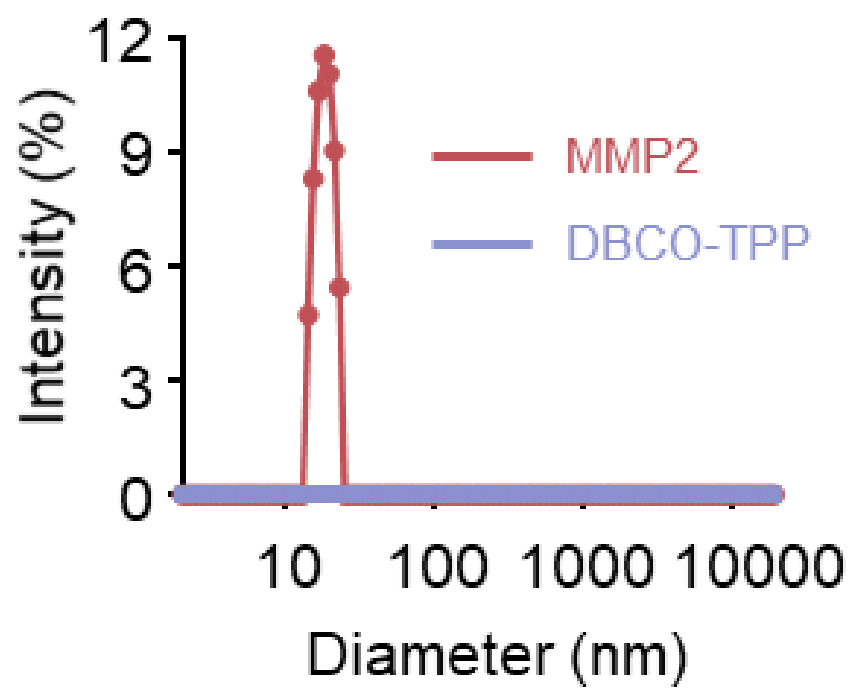

Fig. S11. Dynamic light scattering (DLS) analysis of DBCO-TPP (50 μM) and MMP2 (1 μg mL^{-1}).

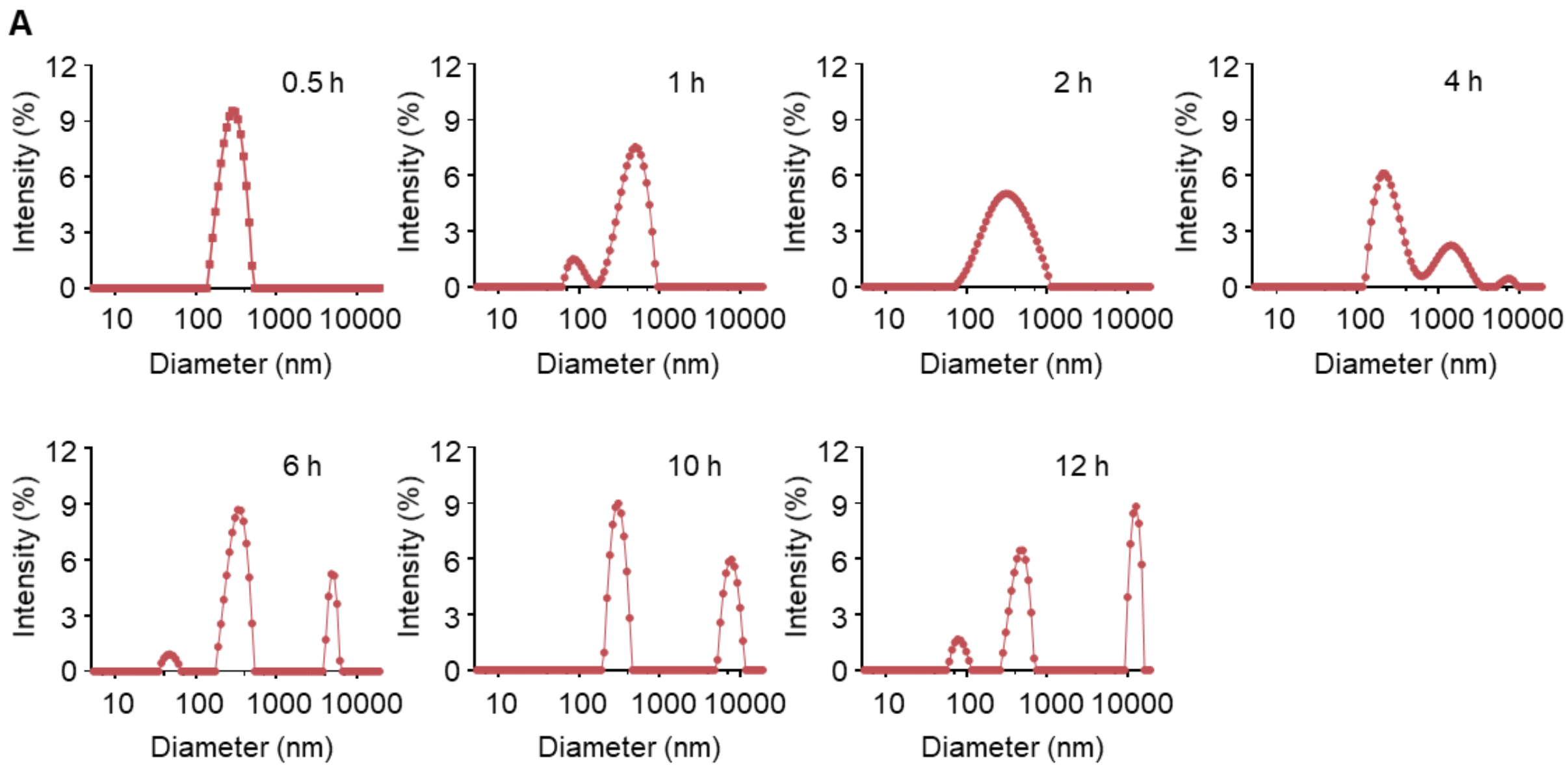

B

Time (h)	Average hydrodynamic diameter (nm)	Polydispersity (PDI)
0.5	346	0.1801
1	388	0.2769
2	396	0.2898
4	426	0.2991
6	465	0.3115
10	602	0.4311
12	705	0.3178

Fig. S12. Time-dependent dynamic light scattering (DLS) analysis of DBCO-TPP (50 μM) incubated with MMP2 (1 μg mL^{-1}), showing the evolution of average hydrodynamic diameter and polydispersity index (PDI) over different incubation times.

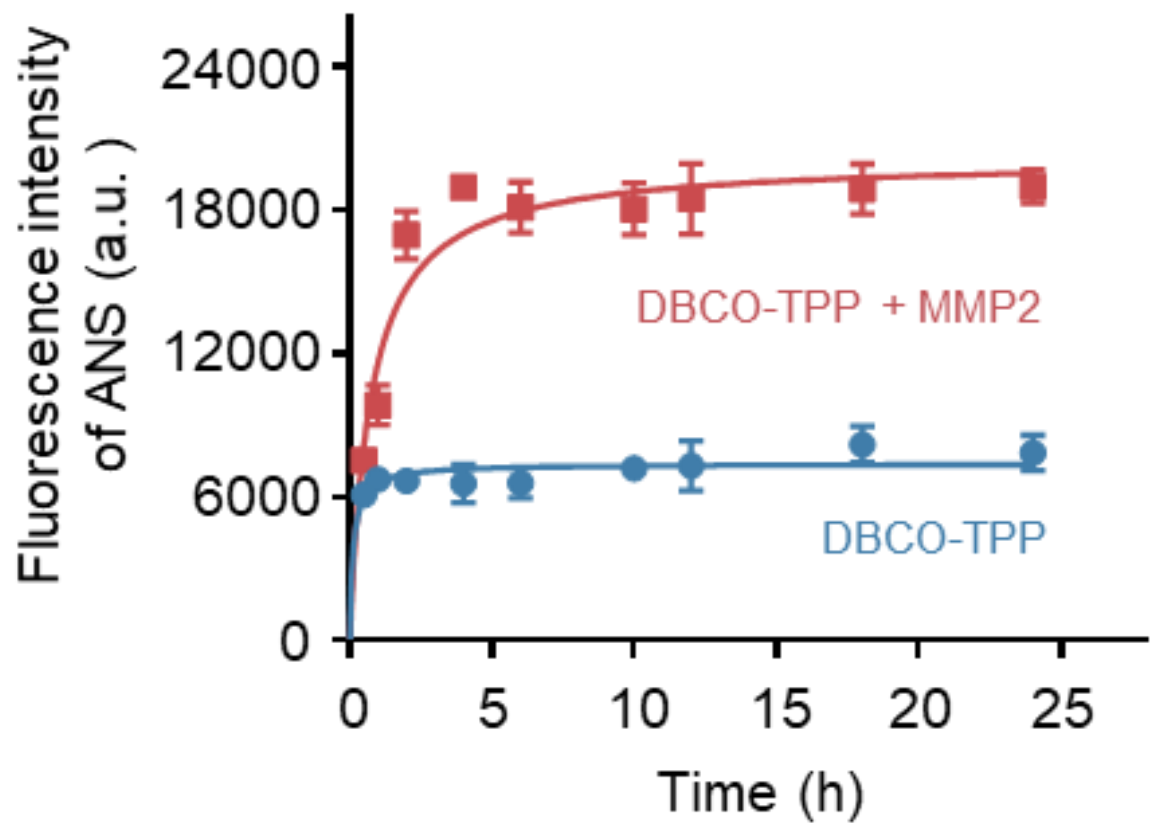

Fig. S13. Kinetic characterization of MMP-2 triggered self-assembly of DBCO-TPP. The ANS (8-Anilino-1-naphthalenesulfonic acid) fluorescence intensity increases gradually over time, reflecting progressive exposure and accumulation of hydrophobic β-sheet domains, with an estimated half-life of approximately 1 h.

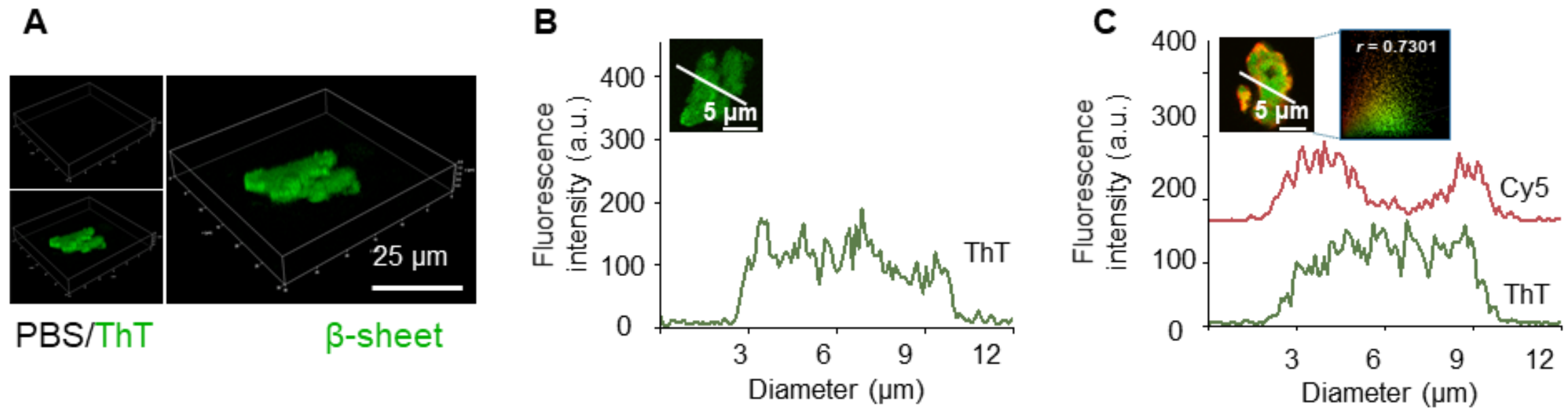

Fig. S14. Validation of biological orthogonal reactions between nanofibers and azide-Cy5. CLSM images (**A**) and corresponding fluorescence spectra (**B**) of nanoDBCO-RSP (50 μM) incubated with ThT (30 μM) and PBS. **(C)** The fluorescence spectra of nanoDBCO-RSP (50 μM) incubated with ThT (30 μM) and azide-Cy5 (60 μM). Pearson correlation coefficient = 0.73.

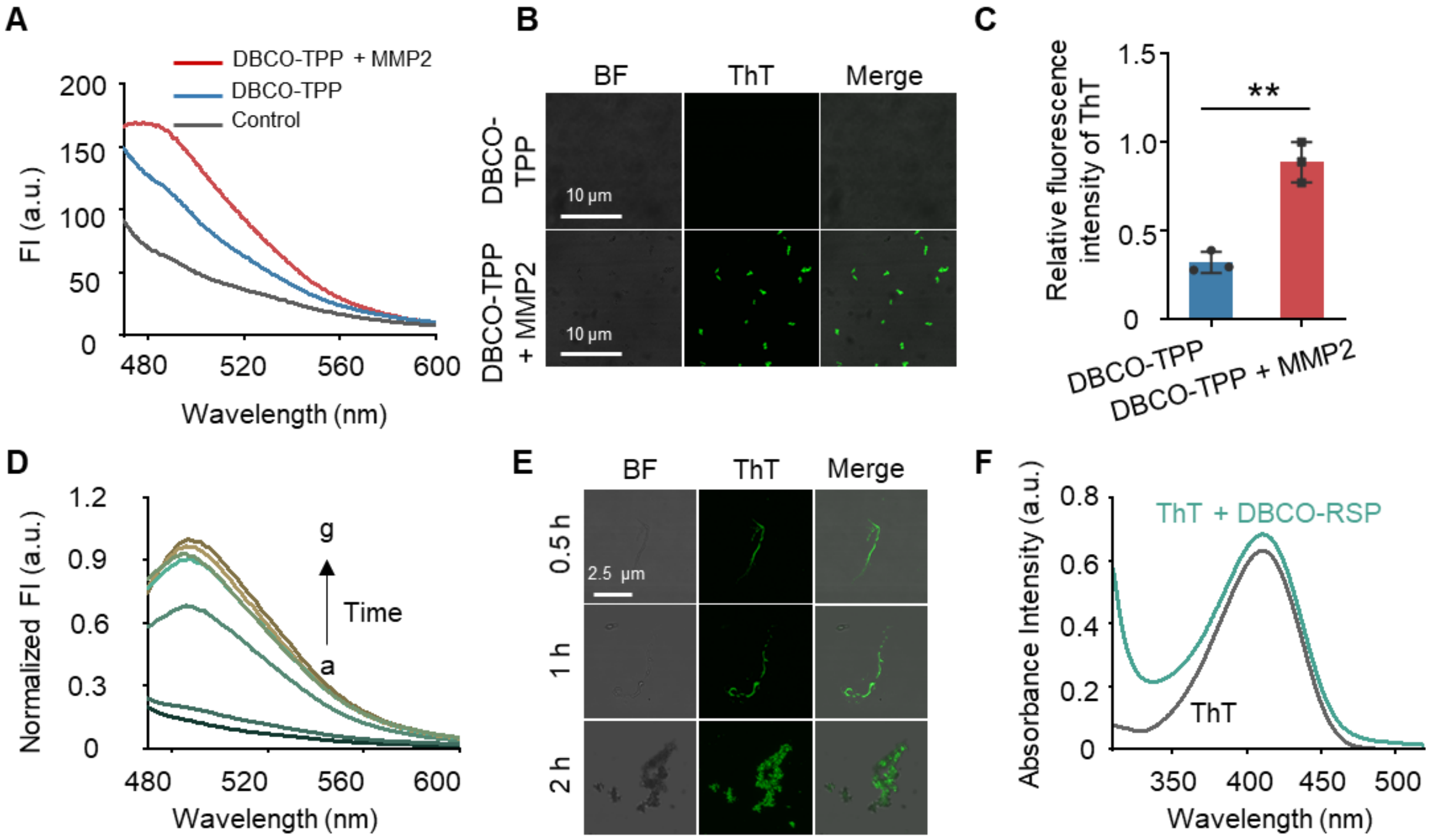

Fig. S15. ThT detection. (A-C) The CLSM images and relative FI of DBCO-TPP and DBCO-TPP + MMP2. **(D)** The fluorescence spectra of ThT reacted with DBCO-RSP (50 μM) assembled in ultrapure water for different times (a → g: ThT, 0, 0.5, 2, 4, 10, and 12 hours). **(E)** CLSM images of DBCO-RSP co-incubated with ThT for different times. **(F)** The UV-vis spectra of ThT and ThT co-incubated with DBCO-RSP. As shown in Fig. S15D and 15F, the fluorescence intensity gradually increased and stabilized over time. Furthermore, the UV spectra remained unchanged, providing additional evidence demonstrated that the combination of ThT with DBCO-RSP does not alter its structure.

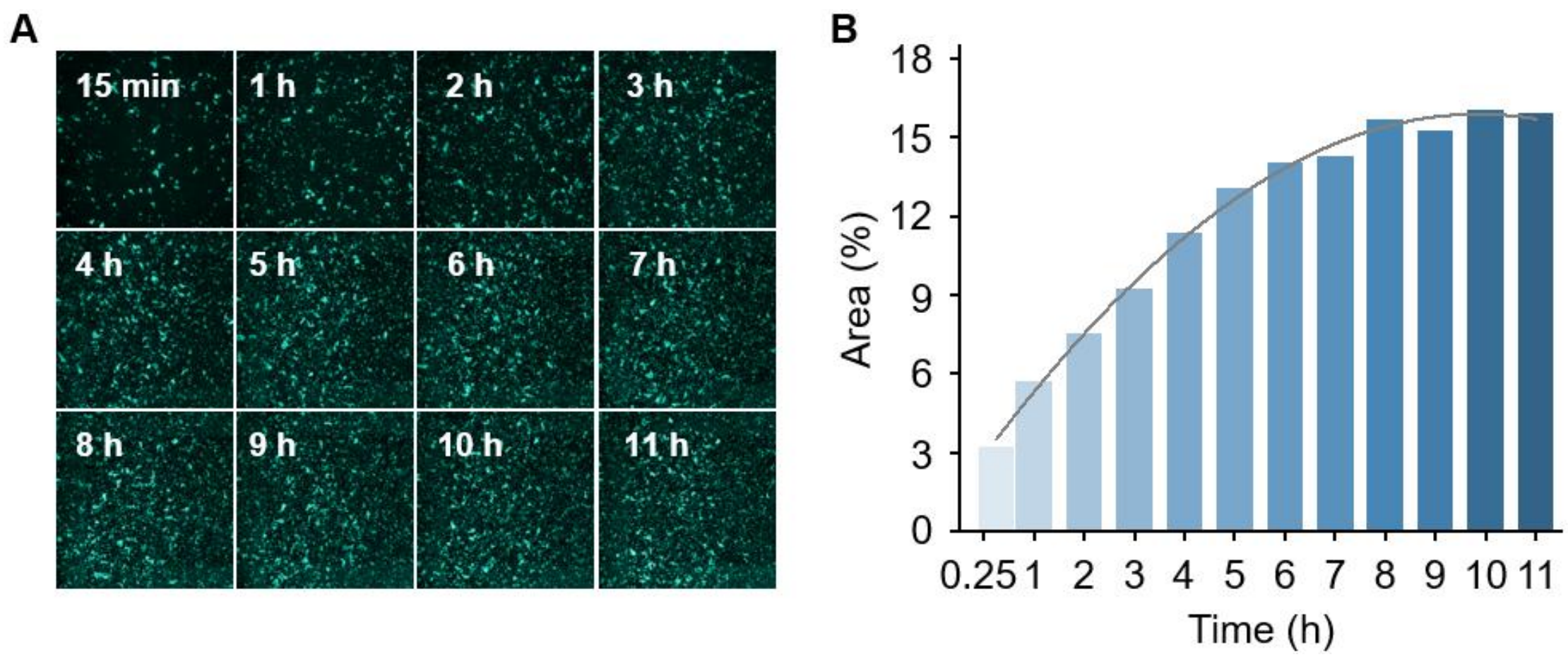

Fig. S16. Validation of β-sheet structure. (A) HCS images of ThT (30 μM) and DBCO-TPP (50 μM) self-assembly in the presence of MMP 2 (5 μg mL^{-1}). **(B)** The quantitative fluorescence area. As shown in Fig. S17B, the click reaction between DBCO-RSP and coumarin produces intense fluorescence, which was attributed to the fact that the electron donor properties of the 1,2,3-triazole ring generated by the click reaction being enhanced by the significant impact of the coumarin substitutions at positions 3 and 7 on the fluorescence properties.

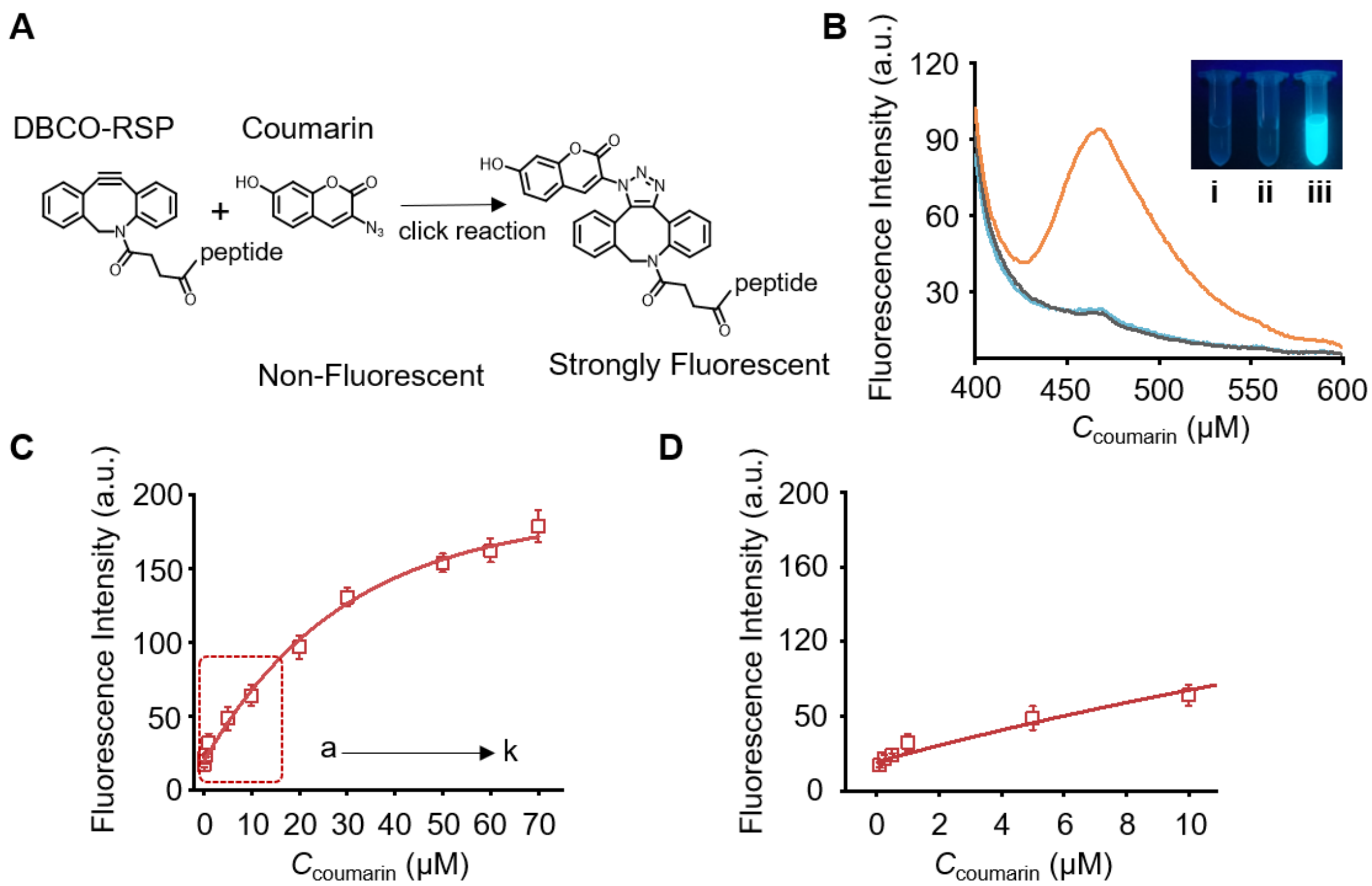

Fig. S17. Validation of biological orthogonal reactions. (A) The reaction mechanism diagram of SPAAC between DBCO-RSP and coumarin. **(B)** Fluorescence spectra of DBCO-RSP (i), coumarin (ii), DBCO-RSP and coumarin (iii). Inset: Photograph of i, ii and iii under a 365 nm UV lamp. **(C)** Fluorescence spectra of DBCO-RSP (50 μM) with different concentrations of coumarin (a → k: $C_{DBCO\text{-}RSP}$: $C_{coumarin}$ = 50:0.1, 50:0.25, 50:1, 50:5, 50:10, 50:20, 50:30, 50:40, 50:50, 50:60 and 50:70). **(D)** A magnified view of the lower concentration is shown within the red box.

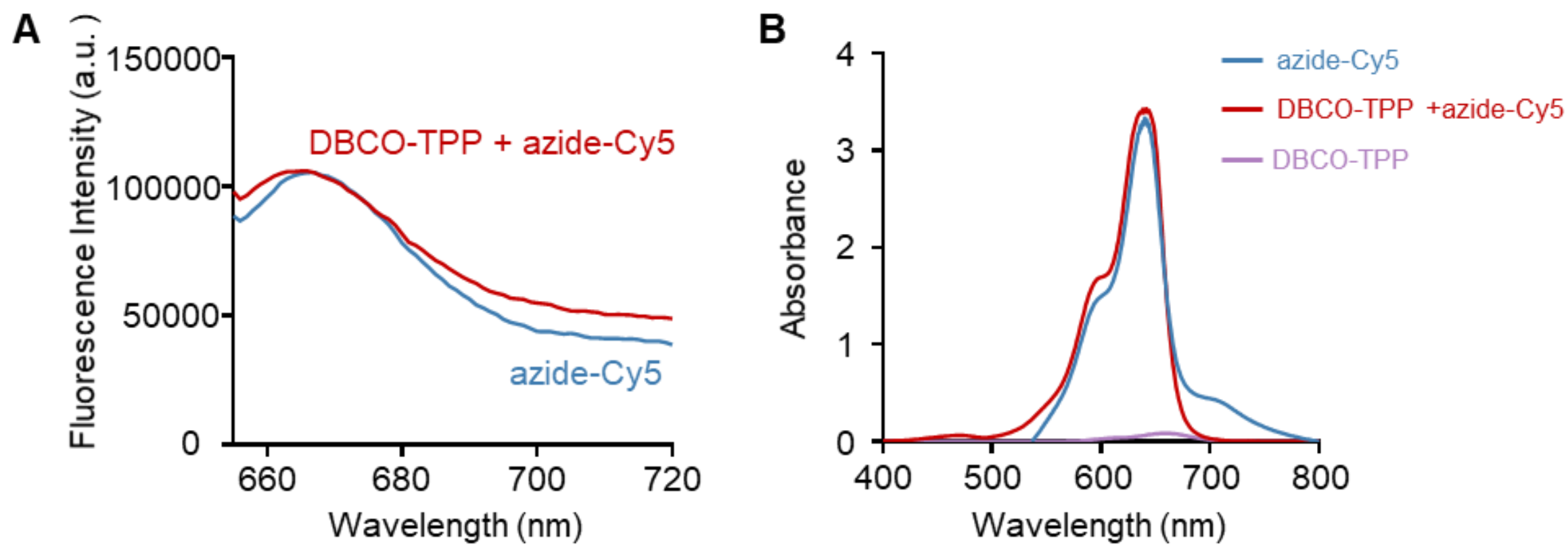

Fig. S18. Evaluation of potential background signals and fluorescence quenching from excess azide-Cy5 (DBCO-TPP : azide-Cy5 = 1:1.5). Blank controls containing only azide-Cy5 and dynamic monitoring of fluorescence intensity over time show negligible changes, indicating minimal interference from unreacted probe. Measurements were performed using both UV-Vis and fluorescence spectroscopy.

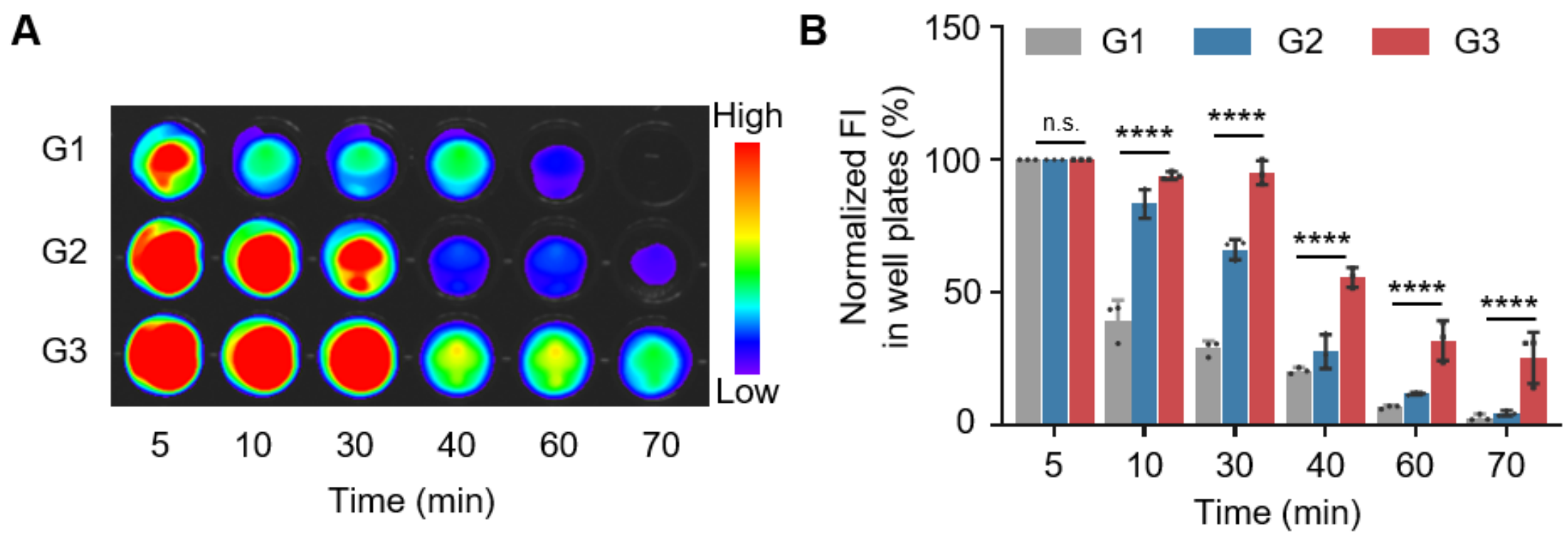

Fig. S19. Validation of photostability. (A) The photostability investigation of three groups under 660 nm laser irradiation with different time (5, 10, 30, 40, 60 and 70 min). **(B)** Normalized time-dependent fluorescence intensity in G1 (PBS), G2 (DBCO-TPP, 500 μM), G3 (DBCO-TPP, 500 μM + MMP 2, 10 μg mL^{-1}). The data (n = 3) were expressed as mean ± SD. The image corresponding to the PBS and DBCO-TPP group in this figure is reused from Fig. 2K.

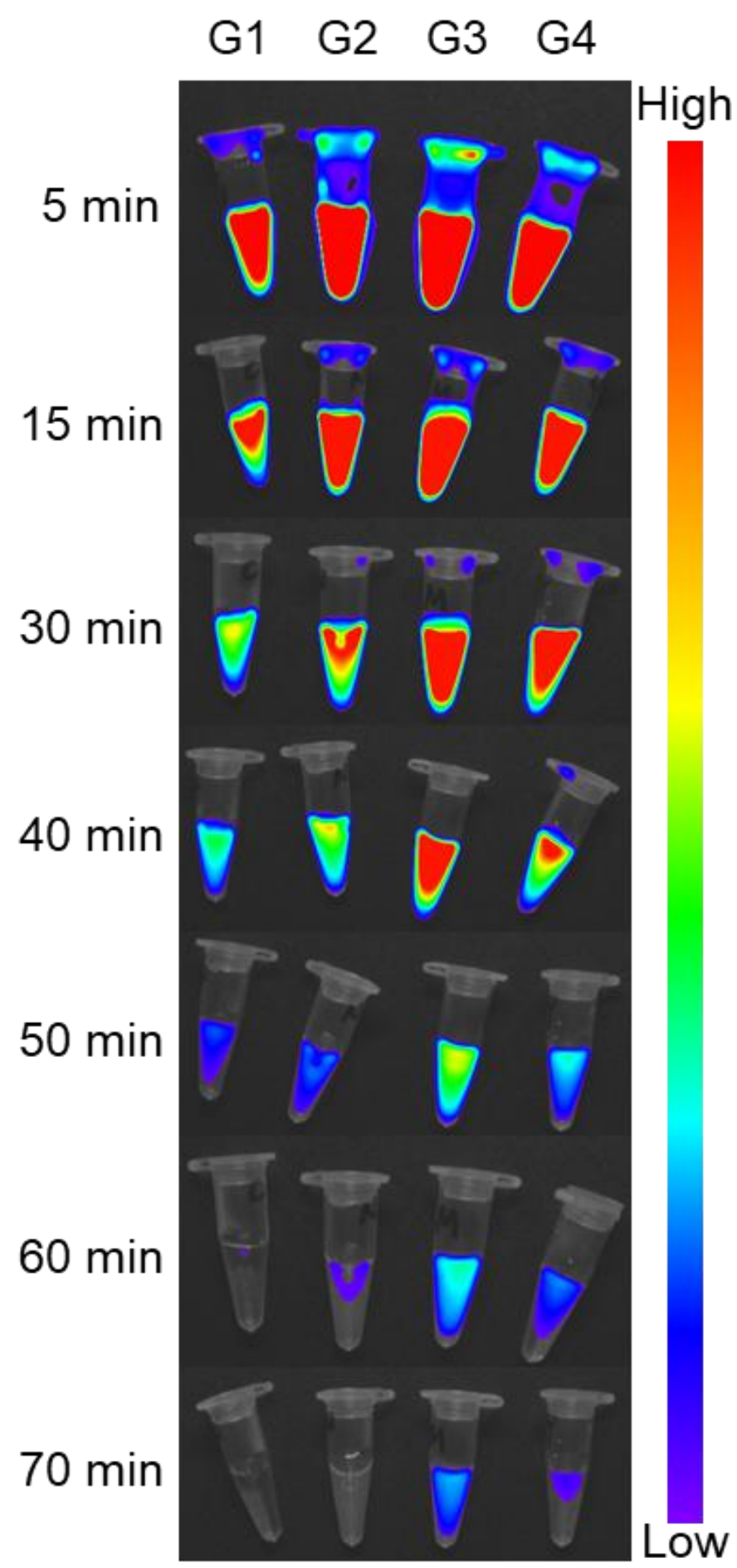

Fig. S20. Characterization of photostability. The photophysical performance of G1 (PBS), G2 (DBCO-TPP, 500 μM), G3 (DBCO-TPP, 500 μM + MMP2, 10 μg mL^{-1}), and G4 (DBCO-RSP, 500 μM) co-incubated with azide-Cy5 (10 μM) for different times.

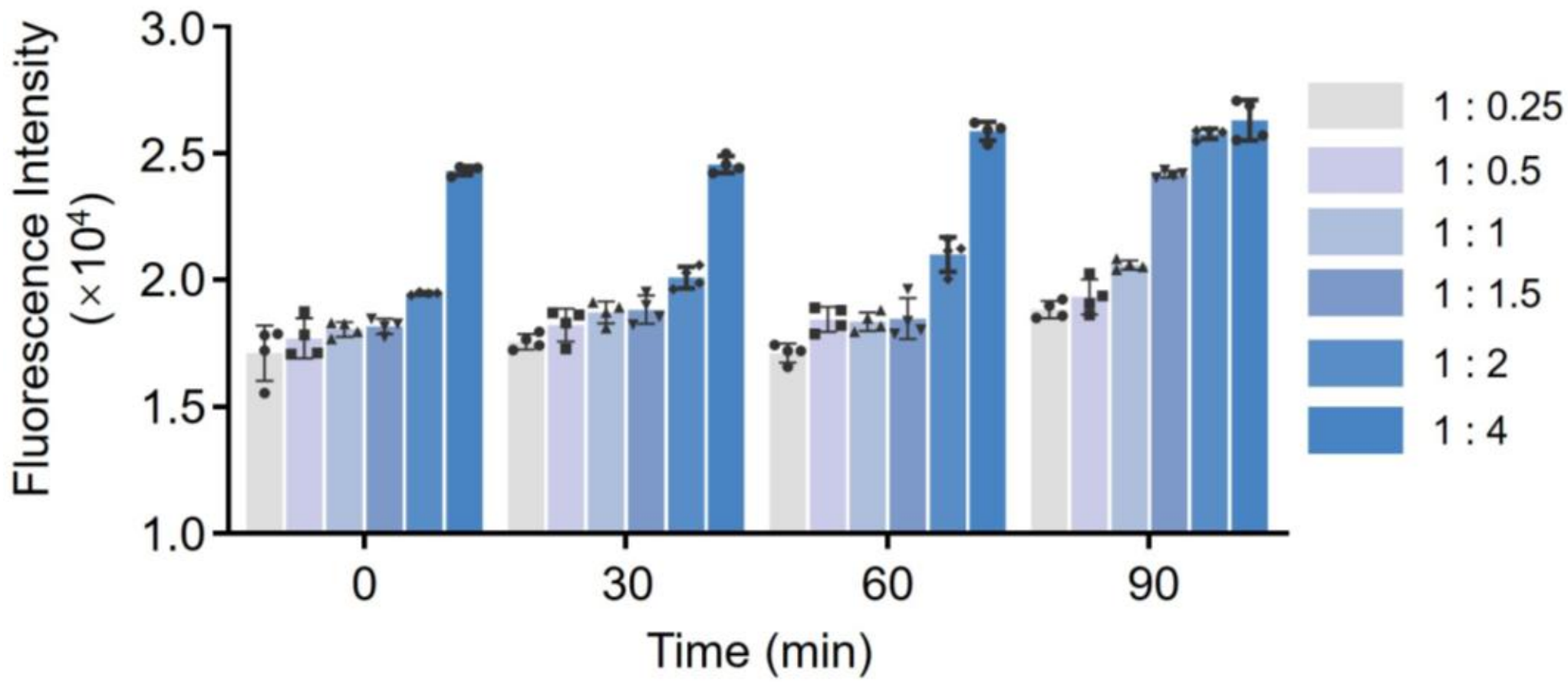

Fig. S21. Optimization of the molar ratio of DBCO-TPP (20 μM) to azide-Cy5. Multiple ratios (1:0.25, 1:0.5, 1:1, 1:1.5, 1:2, and 1:4) at different incubation time were tested, showing that fluorescence intensity remained largely constant between 1:0.5 and 1:2. Although a slight increase was observed at 1:4, 1:1 was selected as the optimal ratio based on reaction kinetics, efficiency, and minimal background interference.

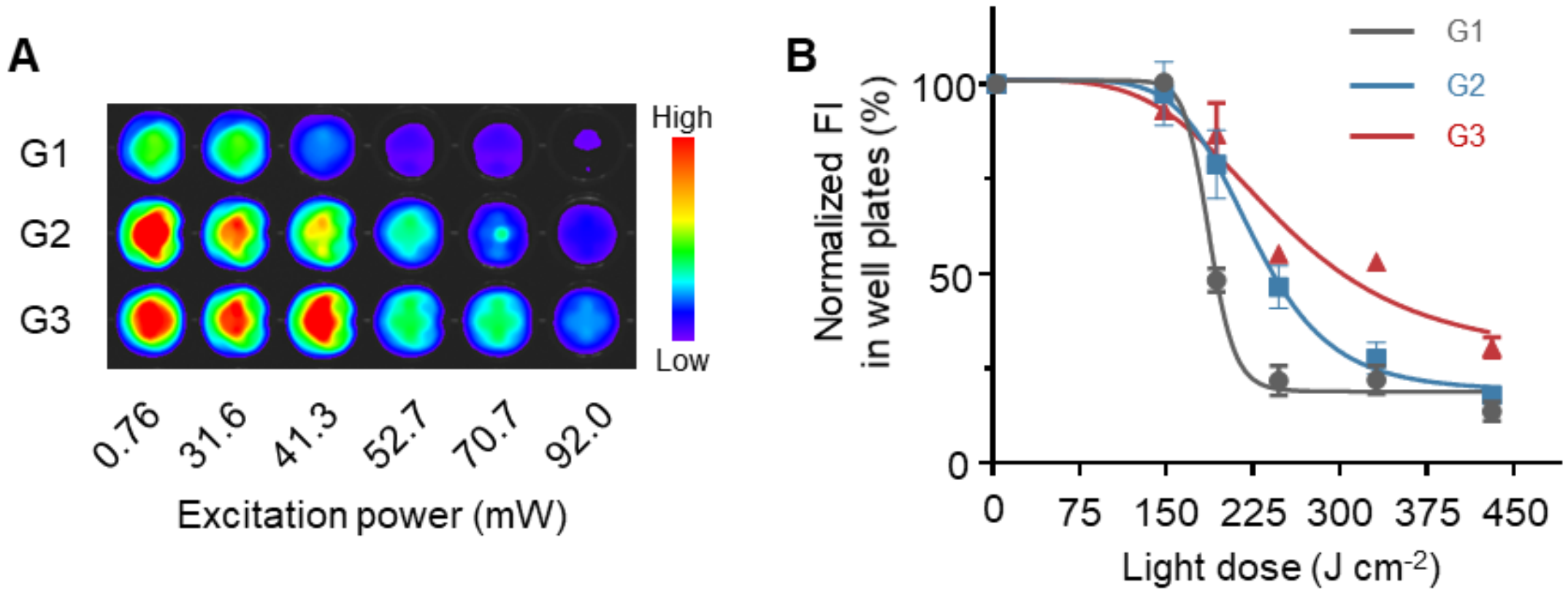

Fig. S22. Light dose-dependent photobleaching analysis. Multiple light dose levels were applied to the G1 (azide-Cy5), G2 (azide-Cy5 + DBCO-TPP), and G3 (azide-Cy5 + MMP2 + DBCO-TPP) groups using different excitation powers (0.76, 31.6, 41.3, 52.7, 70.7, and 92.0 mW) for 30 min, and fluorescence decay was recorded at each light dose.

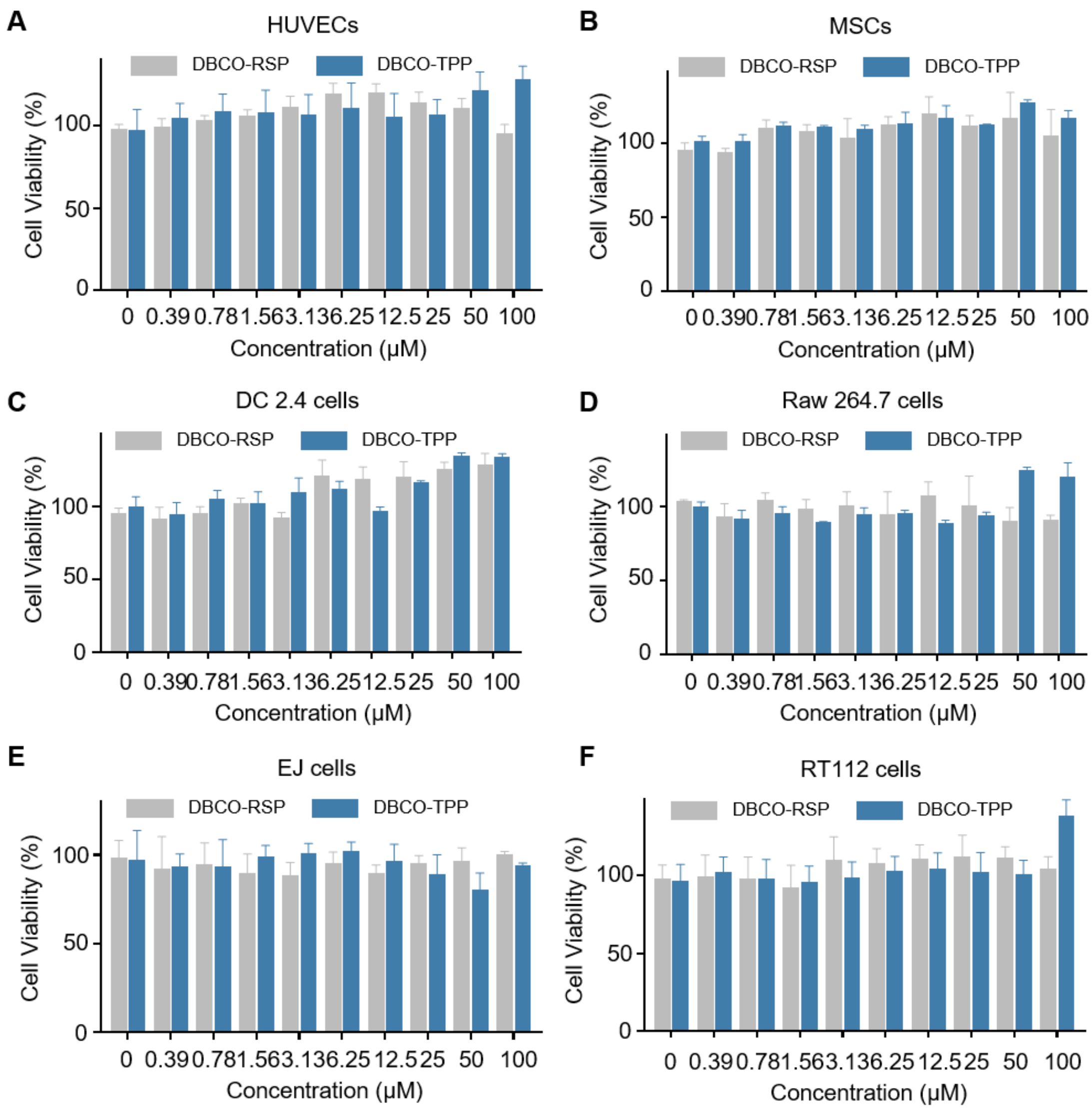

Fig. S23. Cell viability assay. Cell viability of **(A)** HUVECs, **(B)** MSCs, **(C)** DC 2.4 cell, **(D)** Raw 264.7 cell, **(E)** EJ cell and **(F)** RT112 cell treated with different concentrations of DBCO-RSP and DBCO-TPP for 24 h. Results are presented as mean ± SD (*n* = 5).

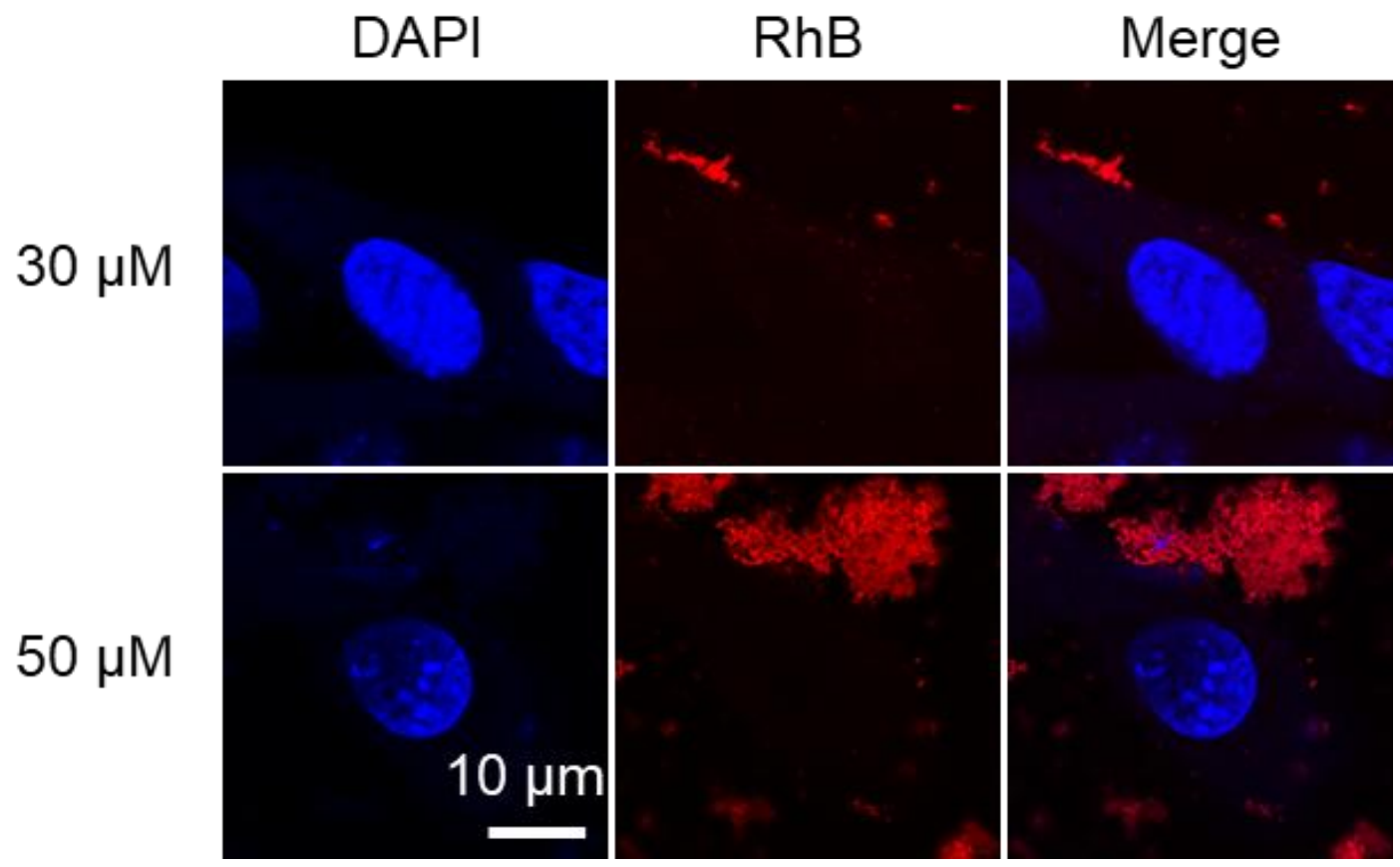

Fig. S24. Optimization of the concentration of the RhB-TPP peptide. CLSM images of RhB-TPP (30 μM and 50 μM) incubated in EJ cells for 2 h. The image corresponding to the group (50 μM) in this figure is reused from fig. S25 (2 h).

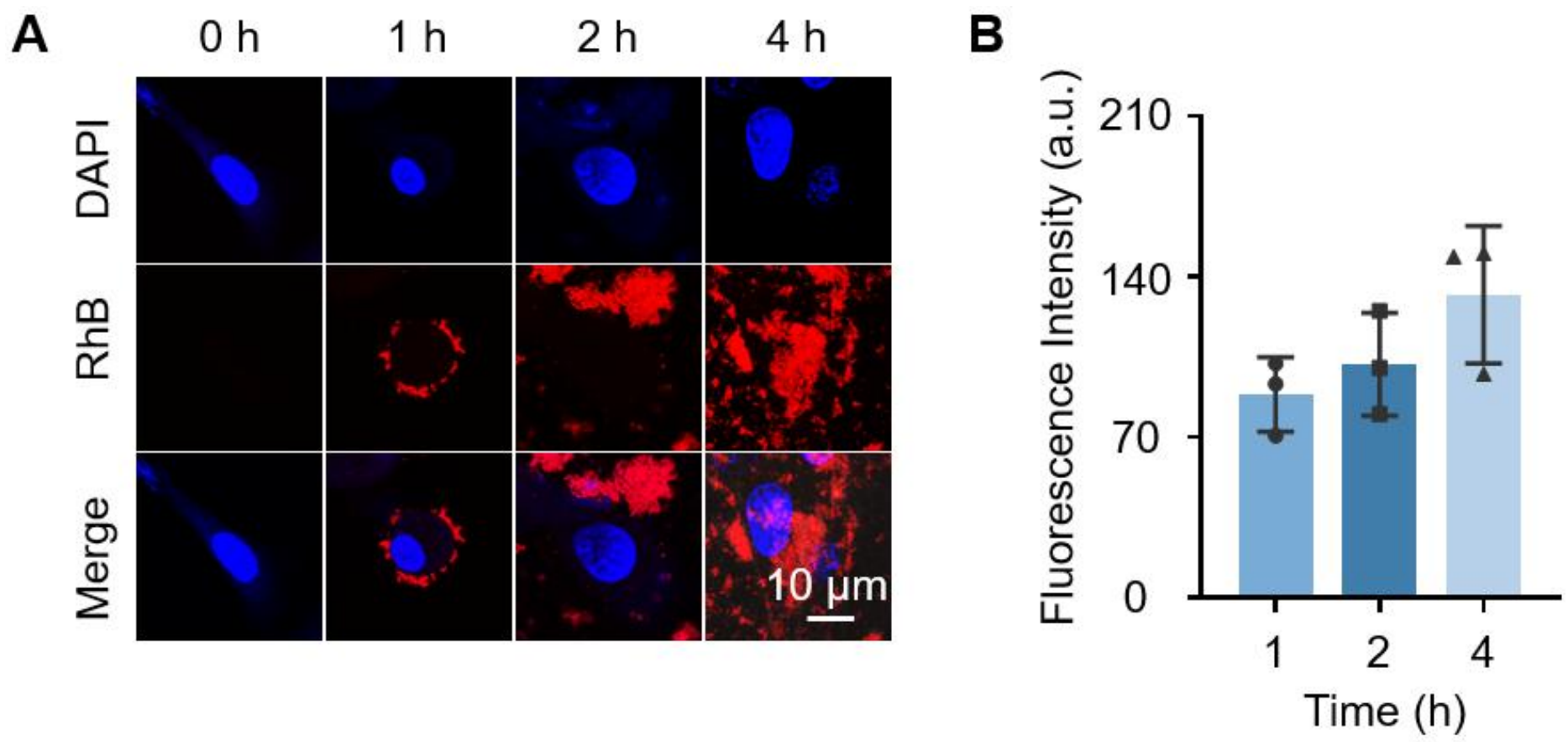

Fig. S25. Optimization of the targeted time of the RhB-TPP peptide. (A) CLSM images and **(B)** quantitative fluorescence intensity of RhB-TPP (50 μM) incubated in EJ cells for 0, 1 h, 2 h, and 4 h. The image corresponding to the group (2h, RhB) in this figure is reused from fig. S24 (RhB, 50 μM).

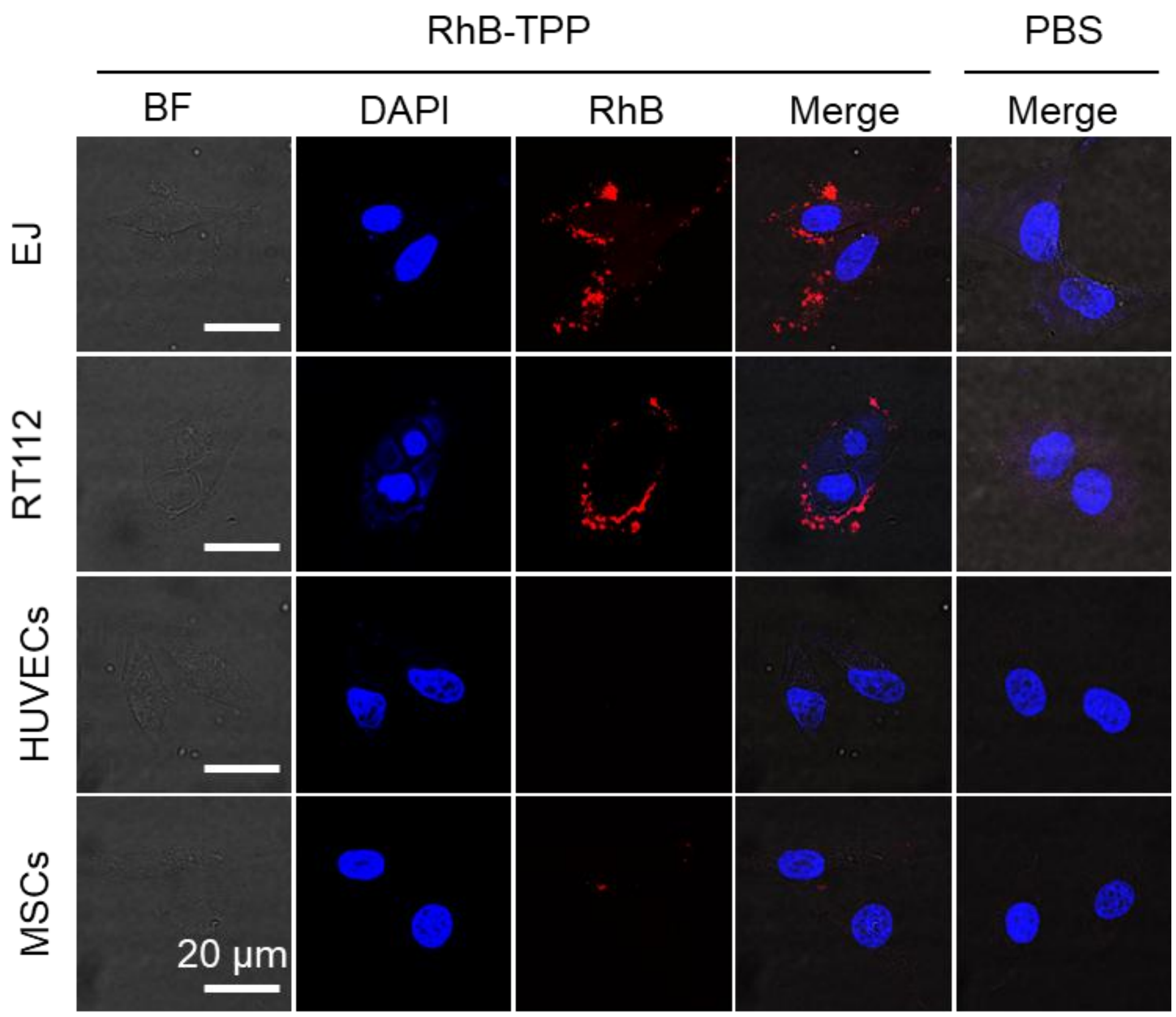

Fig. S26. Targeting validation of TPP. CLSM images of EJ, RT112, HUVECs and MSCs incubated with RhB-TPP (50 μM) and PBS for 2 hours. The image corresponding to the EJ and HUVECs group in this figure is reused from Fig. 3A.

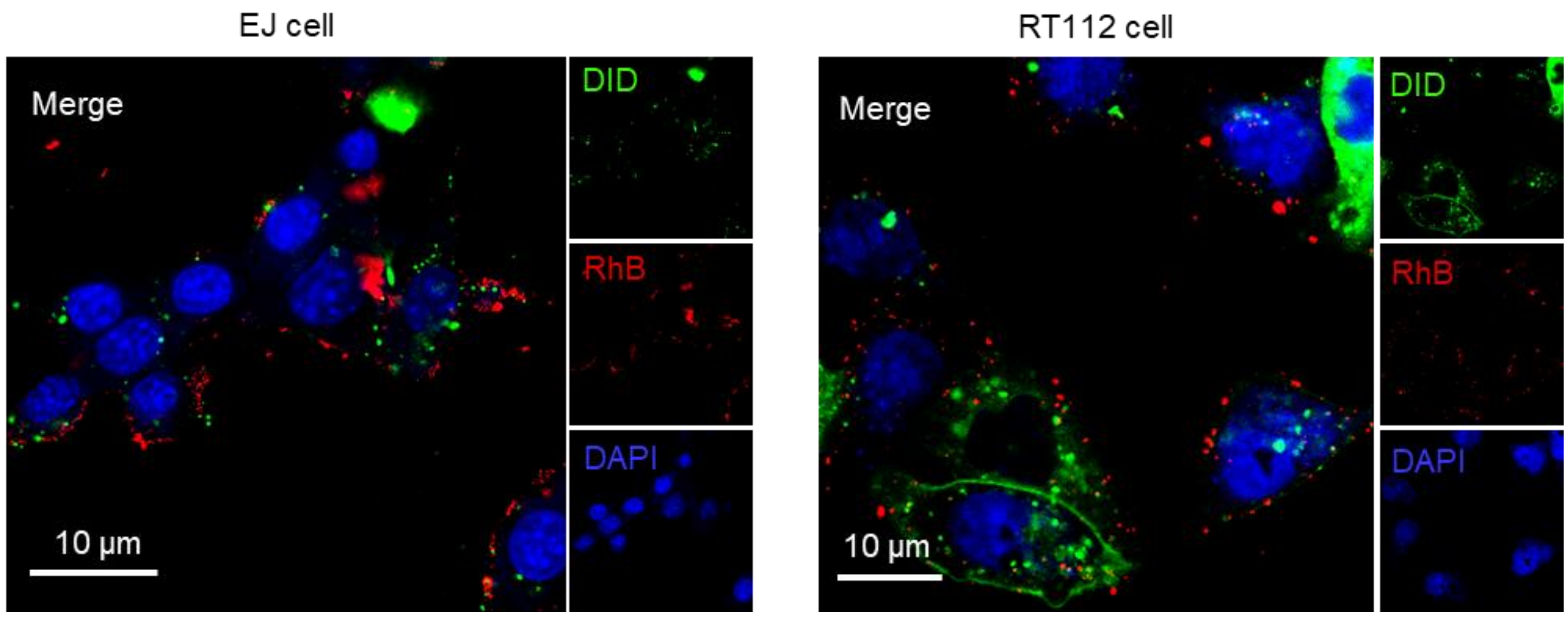

Fig. S27. Confocal laser scanning microscopy (CLSM) images of EJ and RT112 cells after 2 h incubation with RhB-TPP (50 μM), with DiD used as a plasma membrane dye.

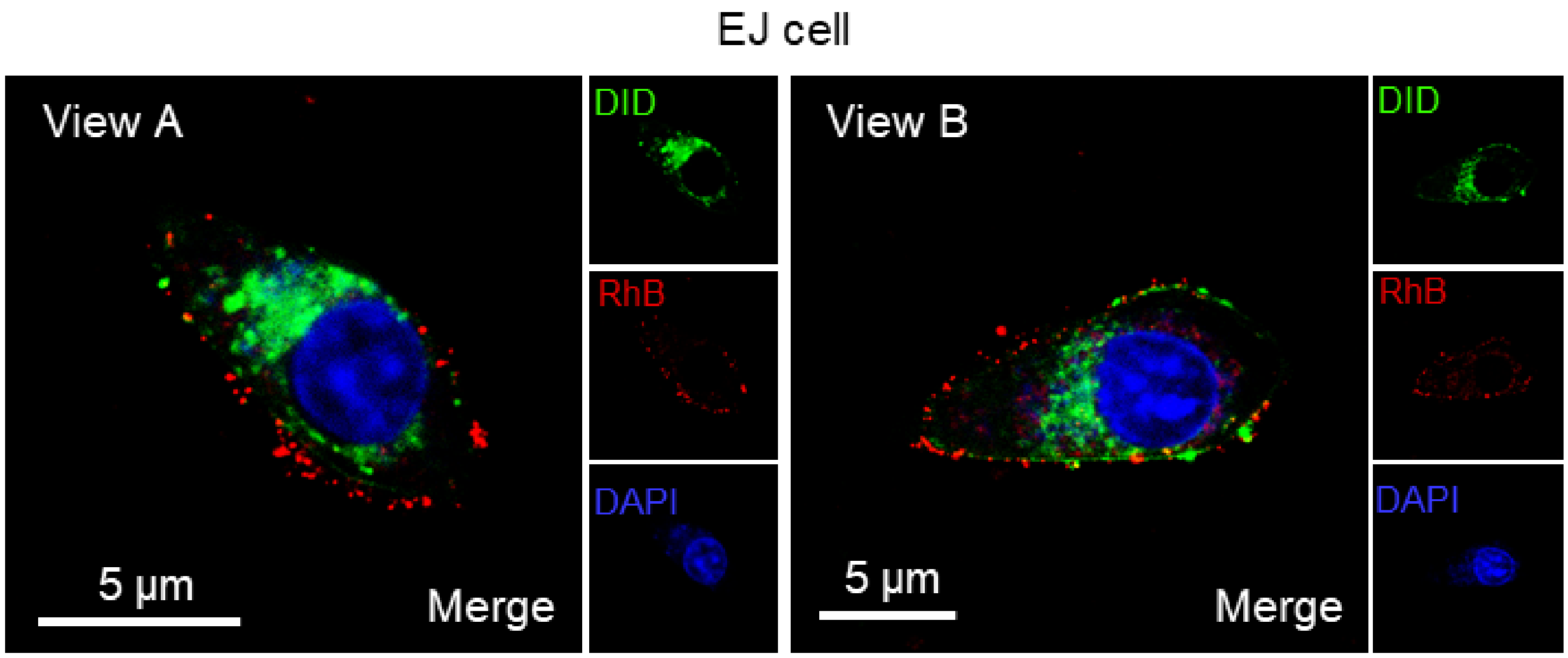

Fig. S28. Confocal laser scanning microscopy (CLSM) images of EJ cells after 2 h incubation with RhB-TPP (50 μM), with DiD used as a plasma membrane dye.

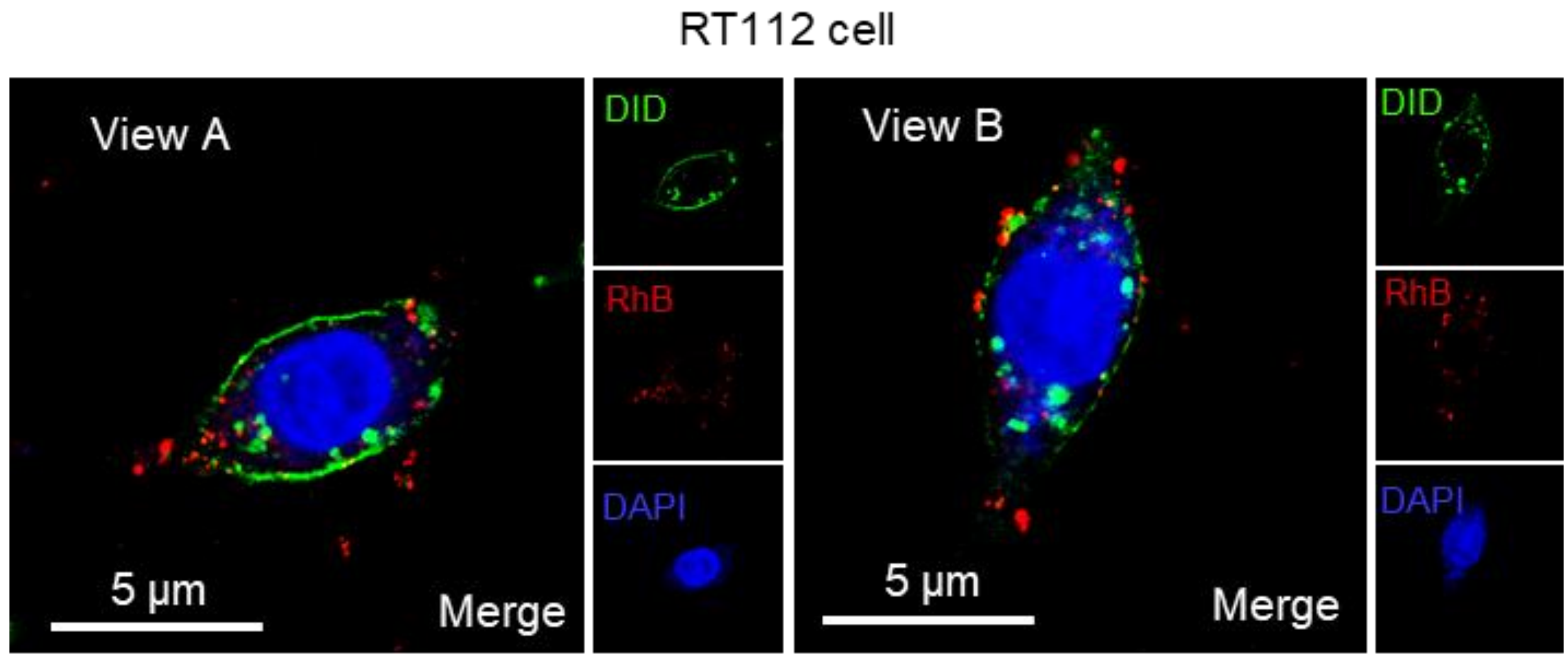

Fig. S29. Confocal laser scanning microscopy (CLSM) images of RT112 cells after 2 h incubation with RhB-TPP (50 μM), with DiD used as a plasma membrane dye.

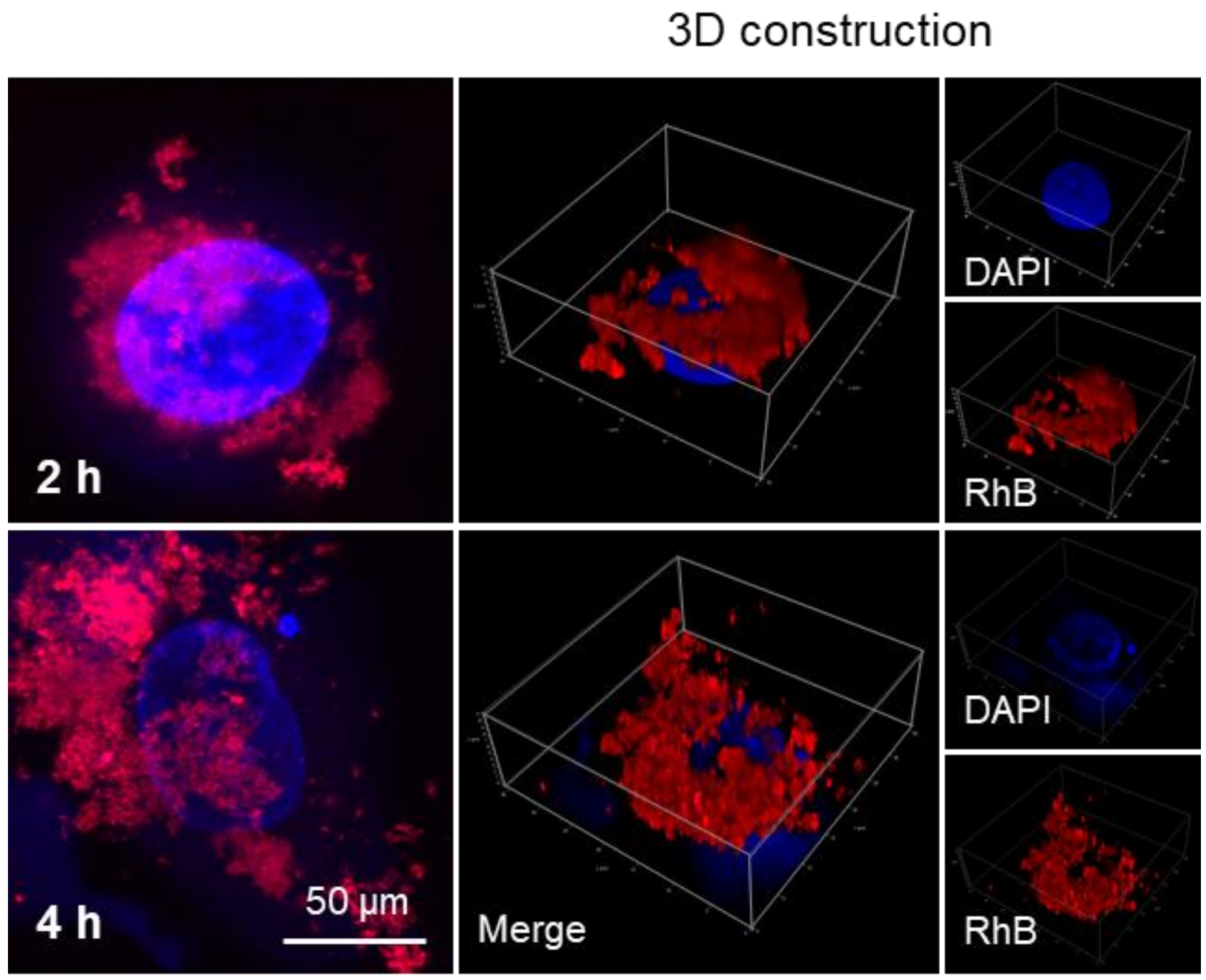

Fig. S30. The 3D construction diagram of RhB-TPP. The 3D construction diagram of the incubation with RhB-TPP (50 μM) in the EJ cells for 2 h.

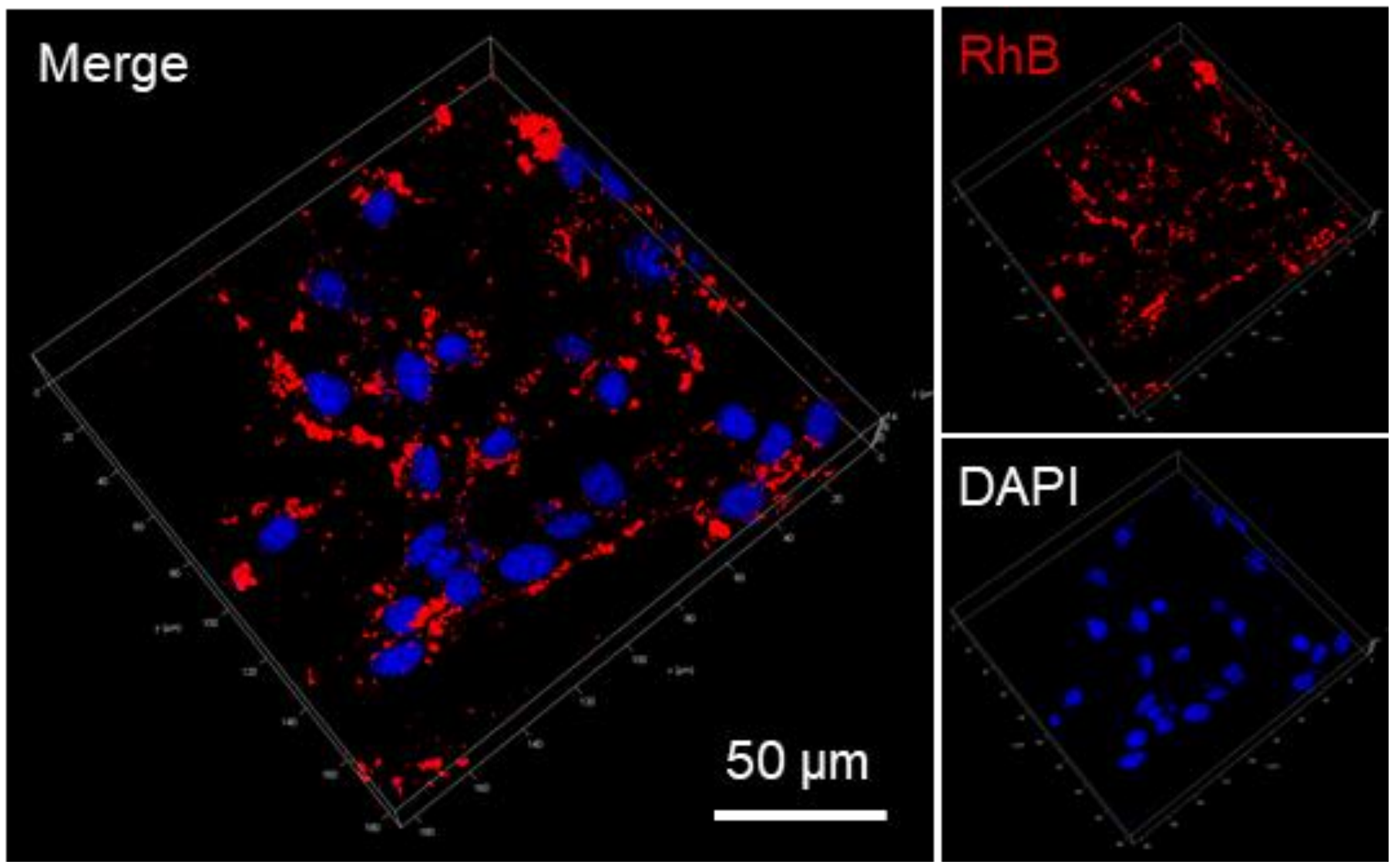

Fig. S31. 3D reconstruction of RhB-TPP in EJ cells after 2 h incubation with RhB-TPP (50 μM), shown across multiple multicellular fields of view.

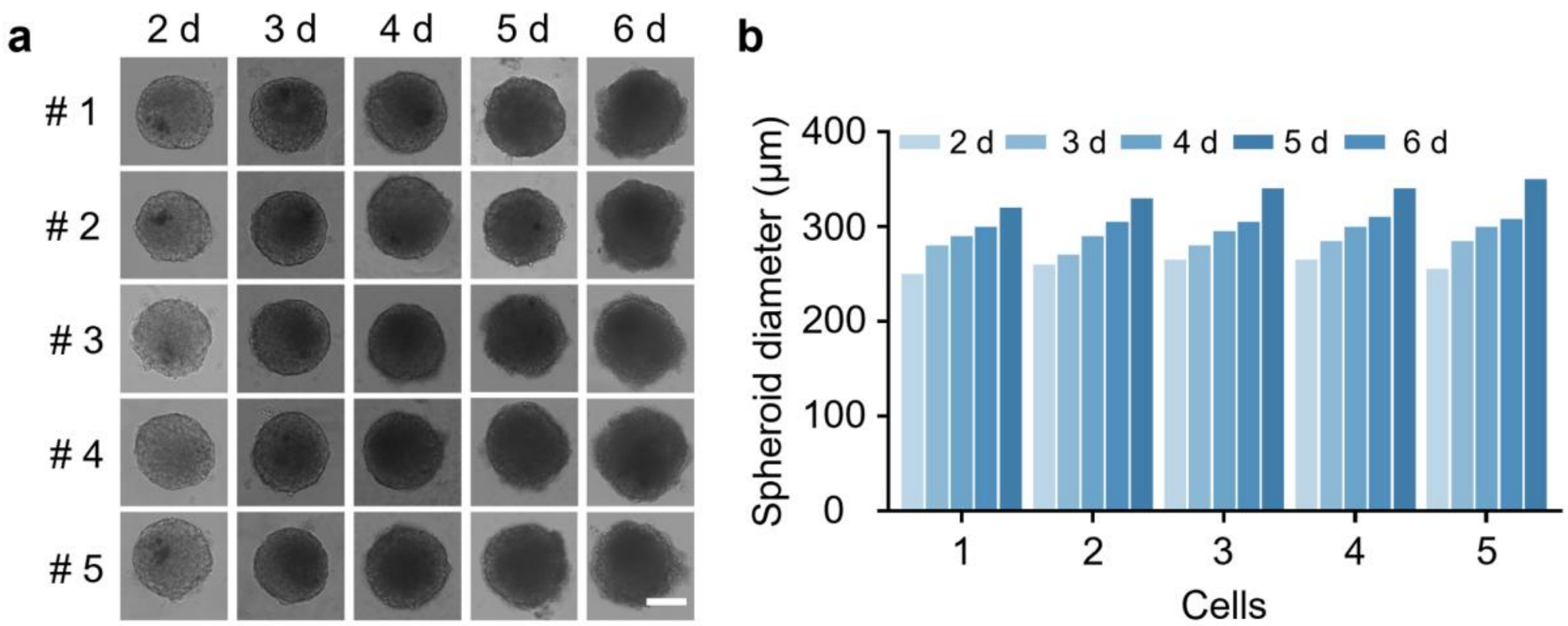

Fig. S32. Culturing of EJ MCTs. (A) Microscope images and **(B)** spheroid diameter of the EJ MCTs cultivation change process. Scale bar: 100 μm.

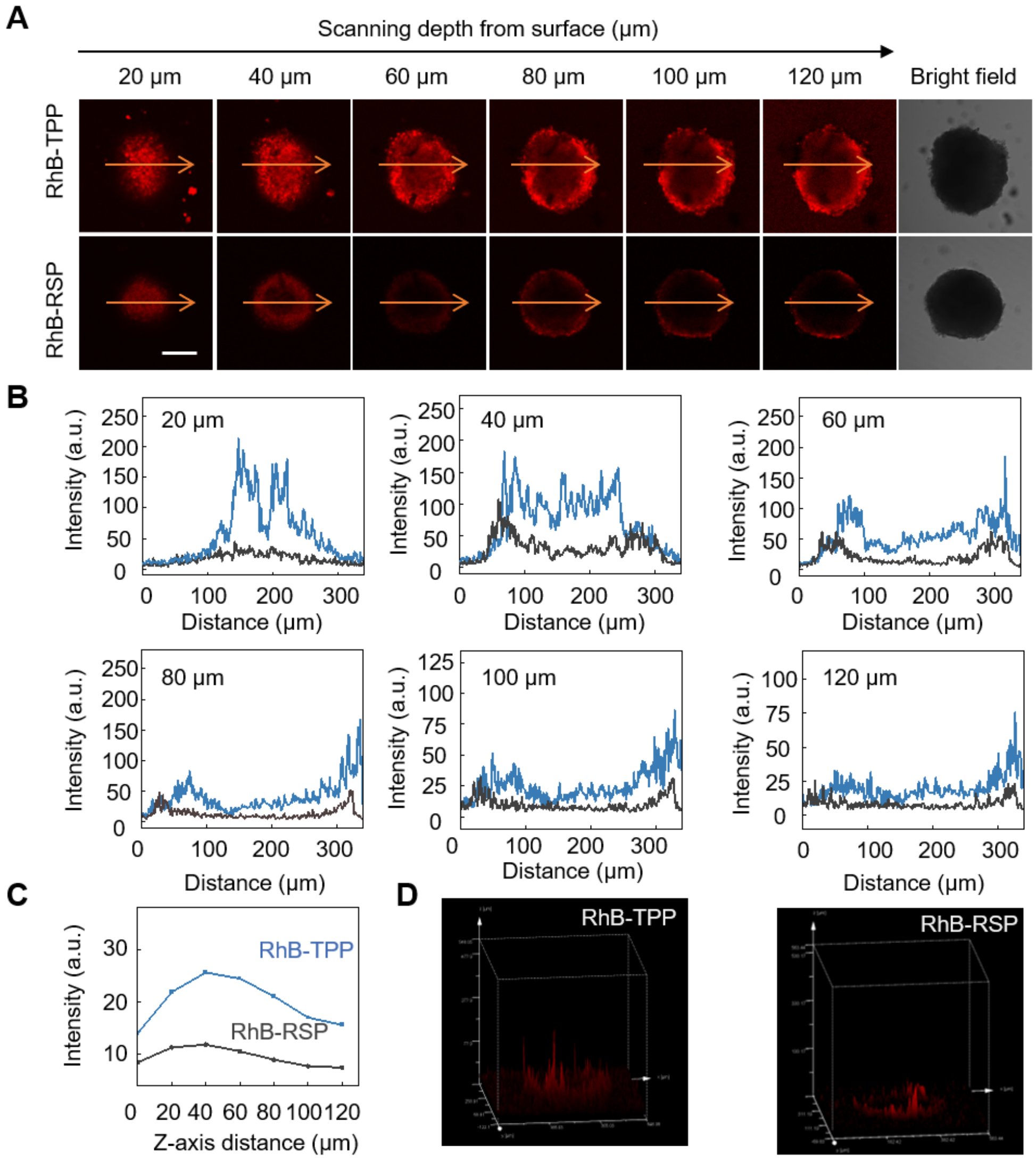

Fig. S33. Evaluation of the penetration performance of peptide in vitro. (A) Z-stack images and **(B-C)** quantitative fluorescence intensity of the permeability of EJ MCTs were cultured with RhB-TPP (50 μM) and RhB-RSP (50 μM) for 0.5 hour. Scale bar: 100 μm. Blue line: RhB-TPP; Black line: RhB-RSP. **(D)** Fluorescent 3D structures of RhB-TPP and RhB-RSP at 120 μm.

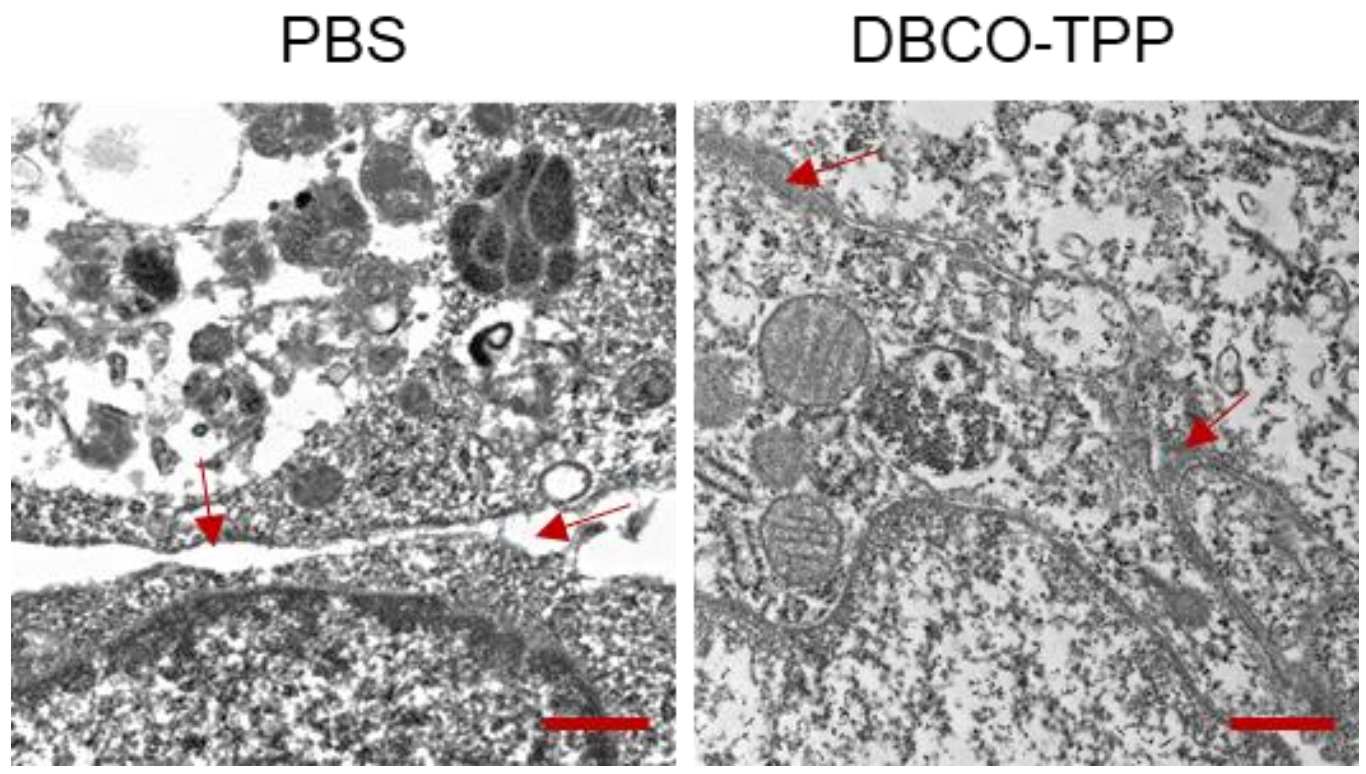

Fig. S34. Bio-TEM characterization. Bio-TEM images of EJ MCTs pretreated with PBS and DBCO-TPP (50 μM). Red arrows represent nanofibers. Scale bar: 500 nm.

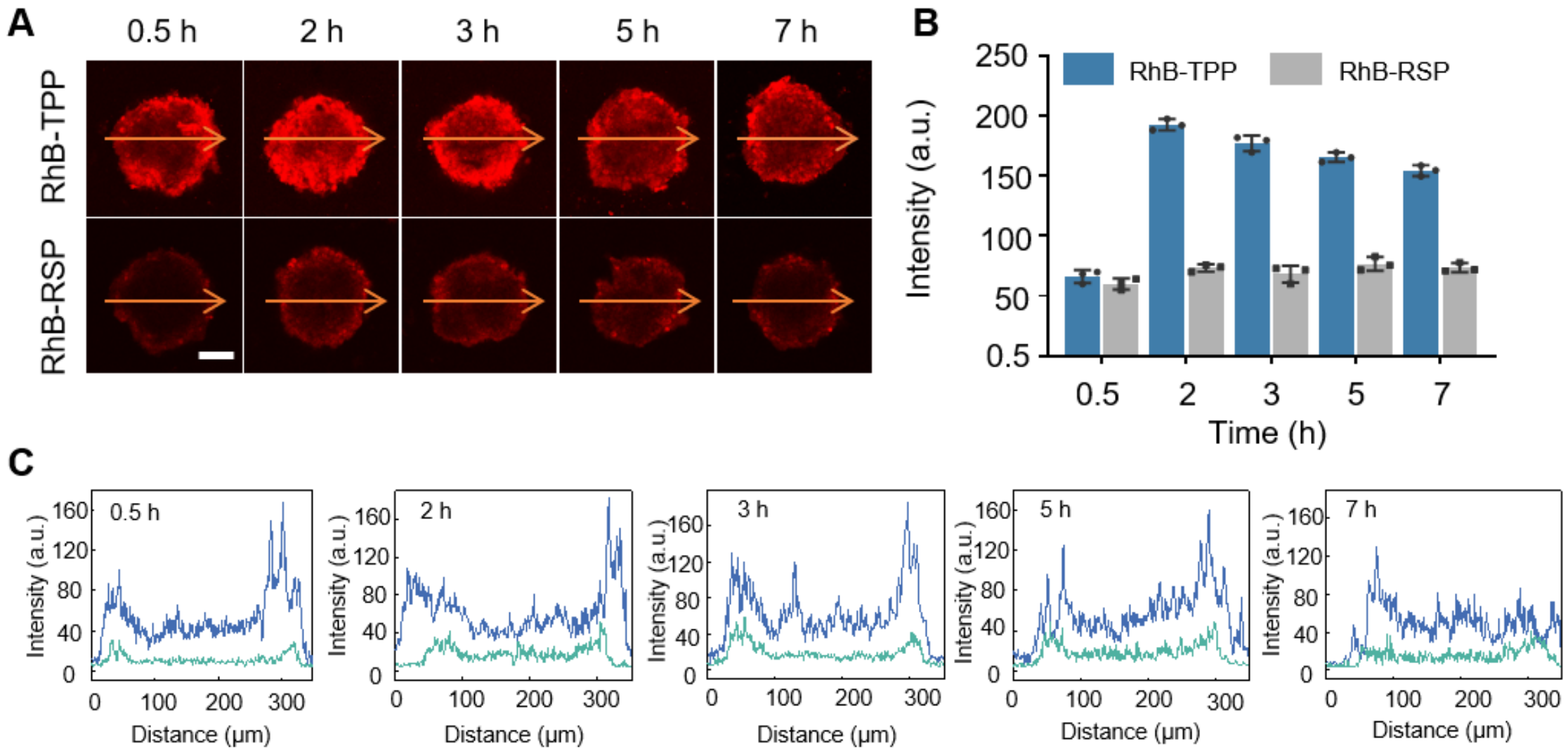

Fig. S35. Evaluation of the retention performance of peptide in vitro. (A) Z-stack images of EJ MCTs were cultured with RhB-TPP (50 μM) and RhB-RSP (50 μM) for different incubation times. Scale bar: 100 μm. **(B)** The quantitative fluorescence intensity and **(C)** of RhB-RSP and RhB-TPP in the z-axis distance (60 μm). Blue: RhB-TPP, Green: RhB-RSP.

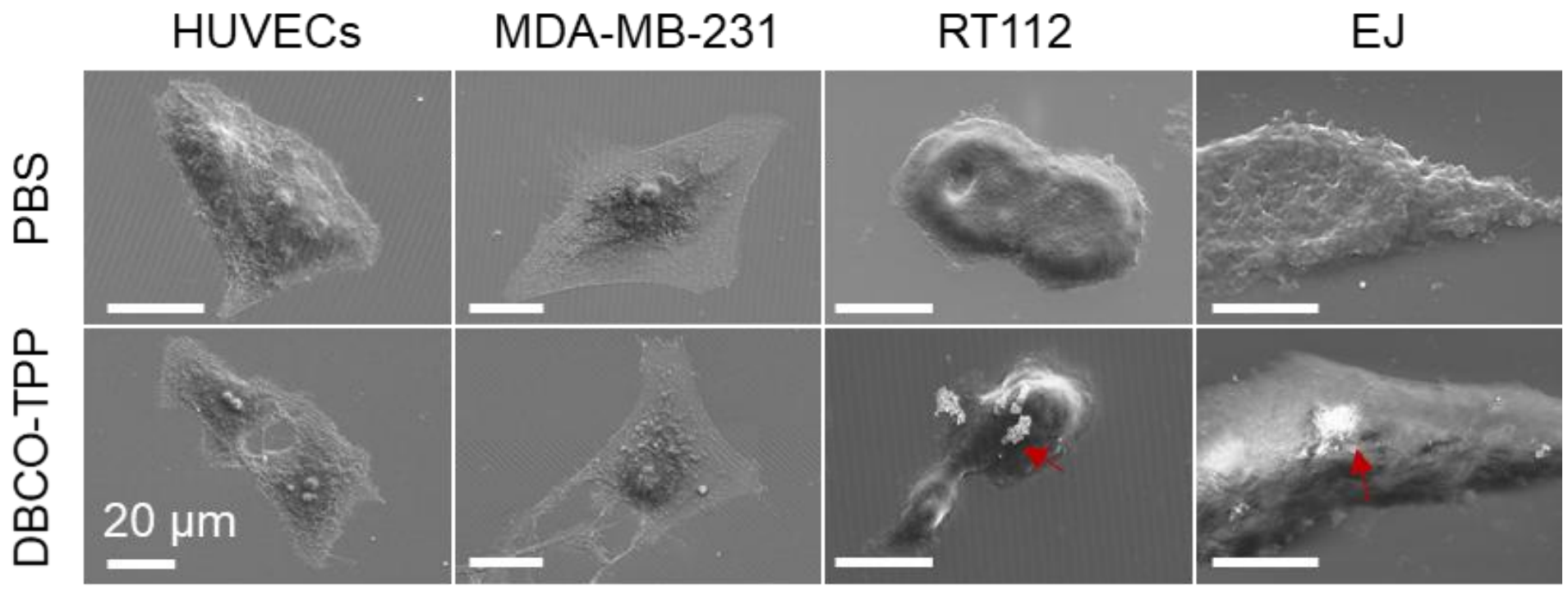

Fig. S36. SEM characterization. SEM images of HUVECs, MDA-MB-231, RT112, and EJ cells treated with PBS and DBCO-TPP (50 μM) for 12 hours. The image corresponding to the EJ group in this figure is reused from Fig. 3H.

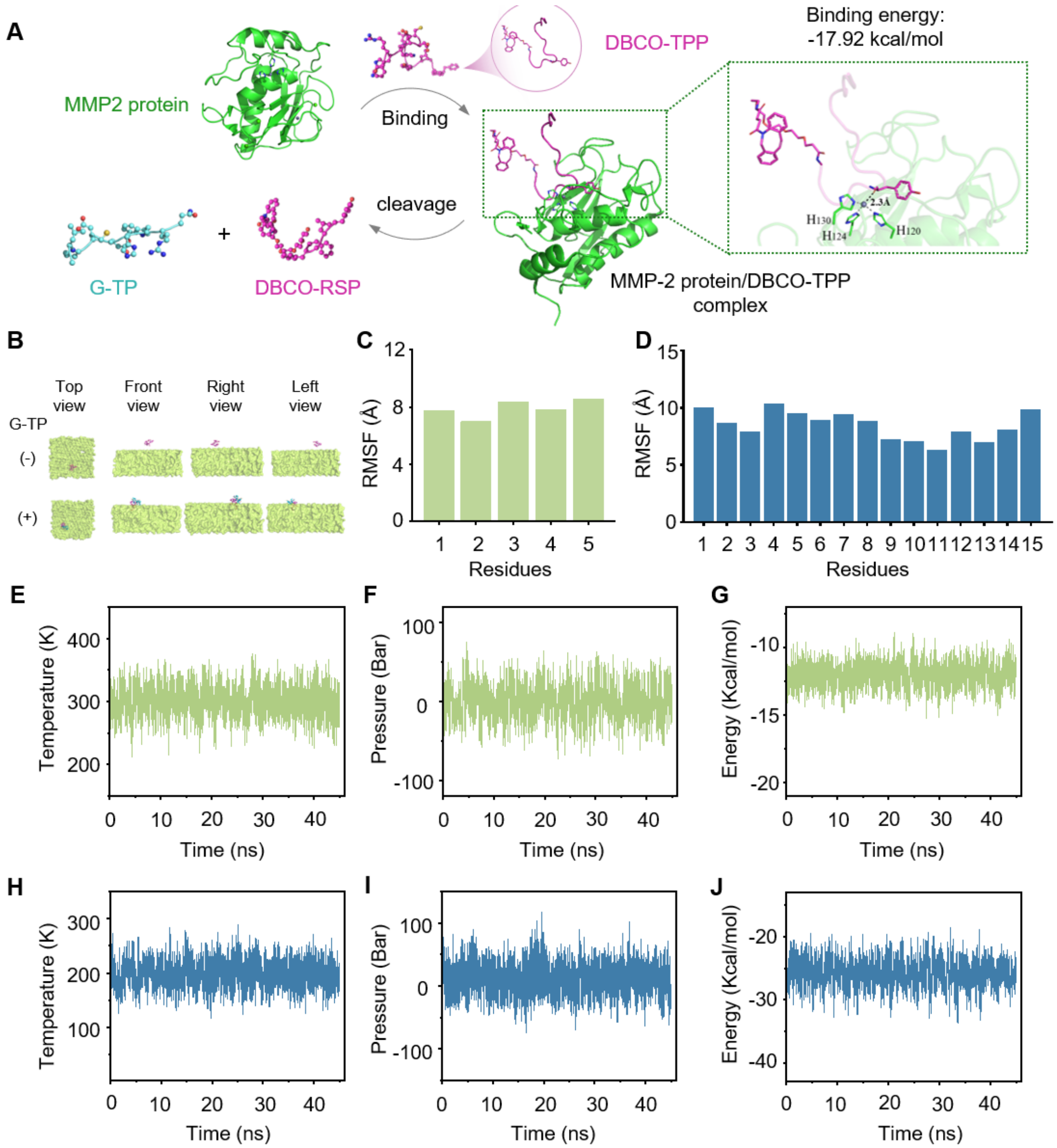

Fig. S37. Interaction of peptides with protein-phospholipid membranes and establishment of ultrasonic force fields in MD simulations. (A) The free DBCO-TPP interacted with the MMP2 enzyme to form the MMP2 protein/DBCO-TPP complex conformation, which is mainly attributed to the ability of -C=O on the amino acid backbone of DBCO-RSP can chelate with the metal ions of the enzyme catalytic center to form a non-covalent and better metal chelating, leading to the tight binding of the free peptide to perform the subsequent catalytic cleavage reaction. Green: MMP2 protein; magentas: DBCO-TPP. **(B)** Representative conformational structures of DBCO-RSP and G-TP within the phospholipid membrane from different perspectives at 45 ns. **(C)** RMSF analysis of DBCO-RSP self-assembled in solution and **(D)** DBCO-RSP pretreated with G-TP for membrane adhesion. Changes in **(E)** temperature, **(F)** pressure and **(G)** total energy of the DBCO-RSP self-assembled system in water. Changes in **(H)** temperature, **(I)** pressure, and **(J)** total energy of the system of DBCO-RSP pretreated with G-TP for adhesion to cell membranes. Results represent three independently performed parallel simulations.

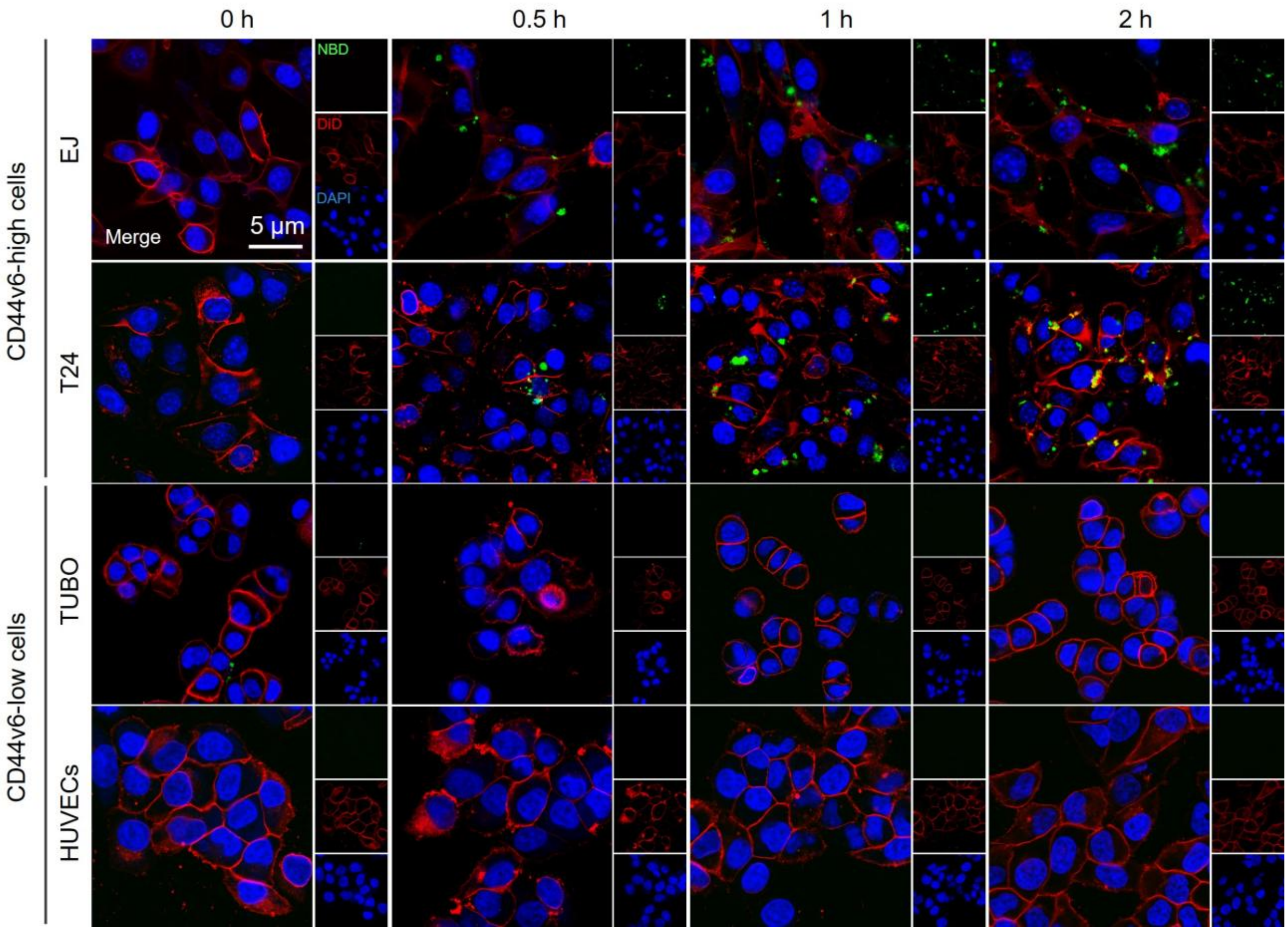

Fig. S38. Visualization of membrane-associated assembly of DBCO-TPP using an environment-sensitive fluorescent probe. Cells (EJ, T24, TUBO, and HUVECs) were incubated with NBD-labeled peptide (NBD-TPP, 75 μM) for 0.5, 1, and 2 h, followed by PBS washing to remove unbound molecules. Confocal laser scanning microscopy (CLSM) images show pronounced punctate NBD fluorescence on the cell membrane in EJ and T24 cells, while negligible fluorescence was observed in TUBO and HUVECs cells.

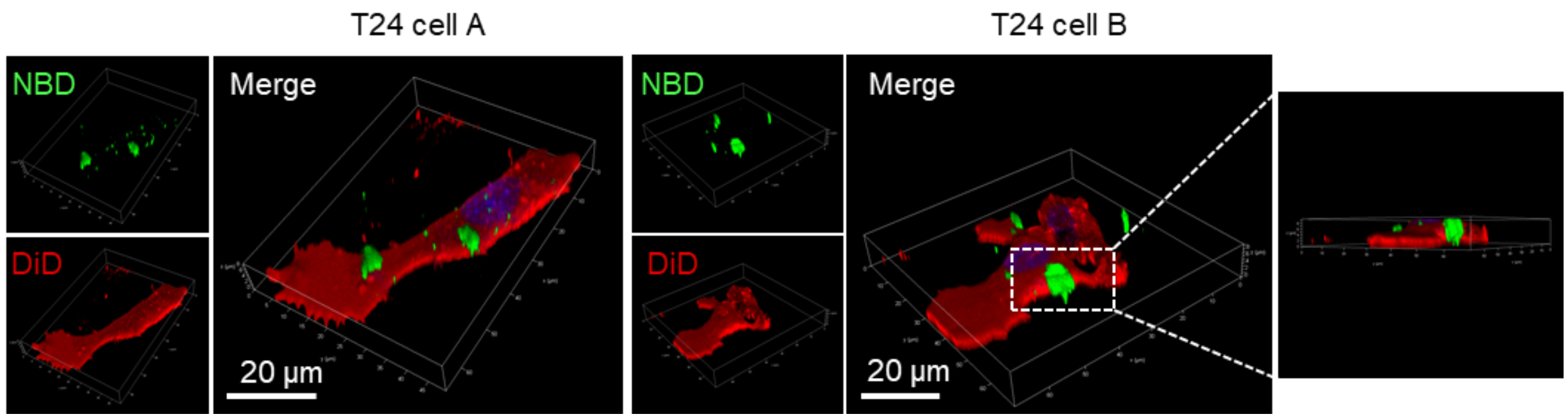

Fig. S39. 3D reconstruction of RhB-TPP distribution in T24 cells. Representative single cell 3D images after incubation with RhB-TPP (50 μM) for 2 h, showing co-localization with DiD (red) and NBD (green) signals across two representative single cell fields of view.

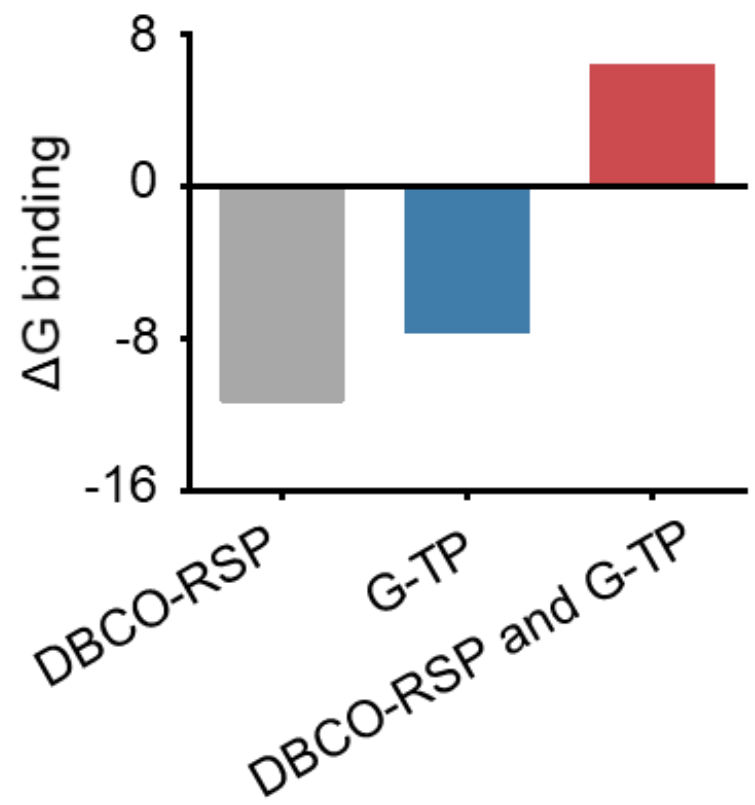

Fig. S40. The calculated ΔG binding energy. The ΔG binding energy between phospholipid membranes and different peptides.

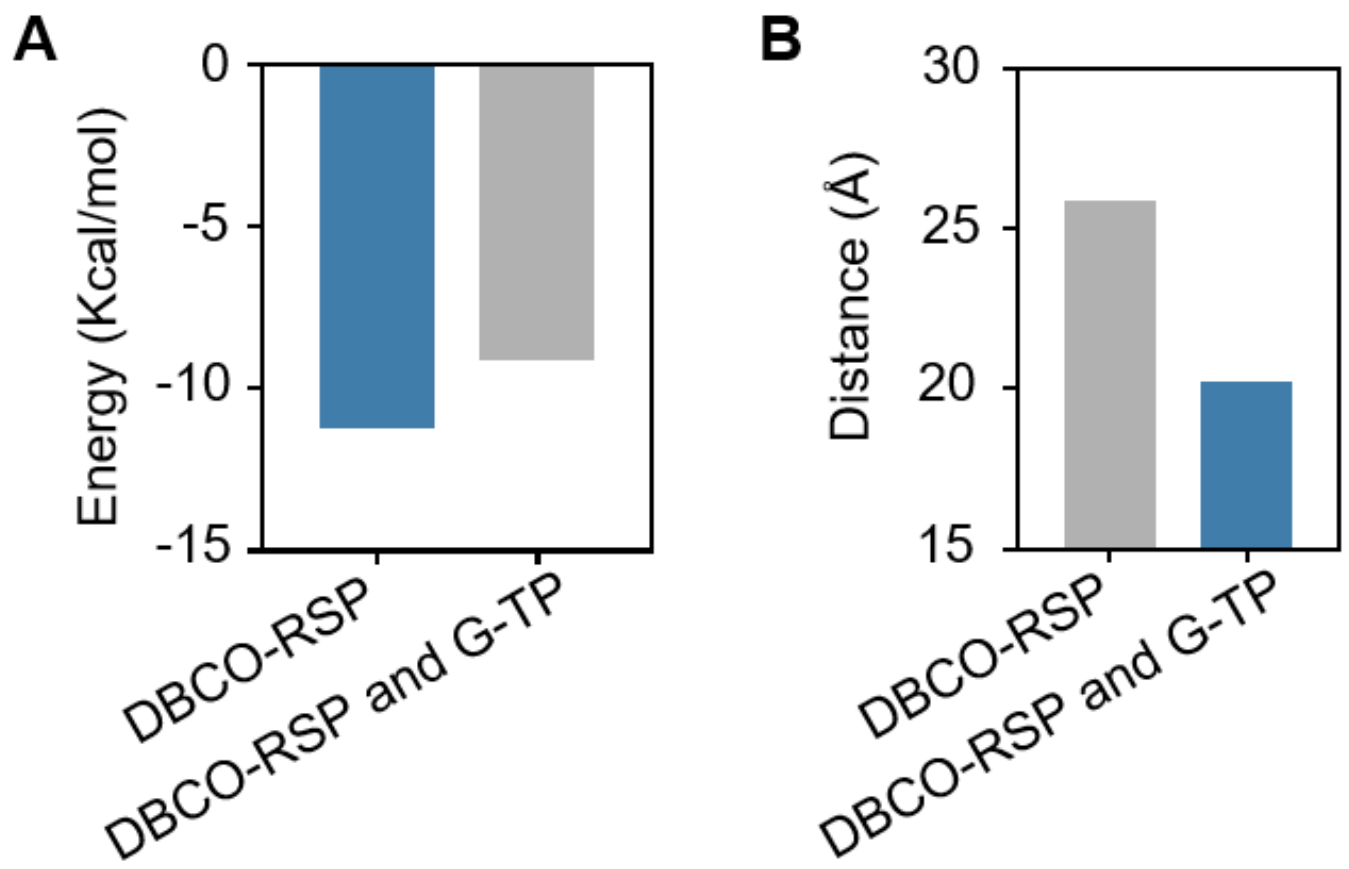

Fig. S41. The binding energy and the distance characterization. (A) Calculating the binding energy and **(B)** the distance between phospholipid bilayer membranes and different peptide molecules.

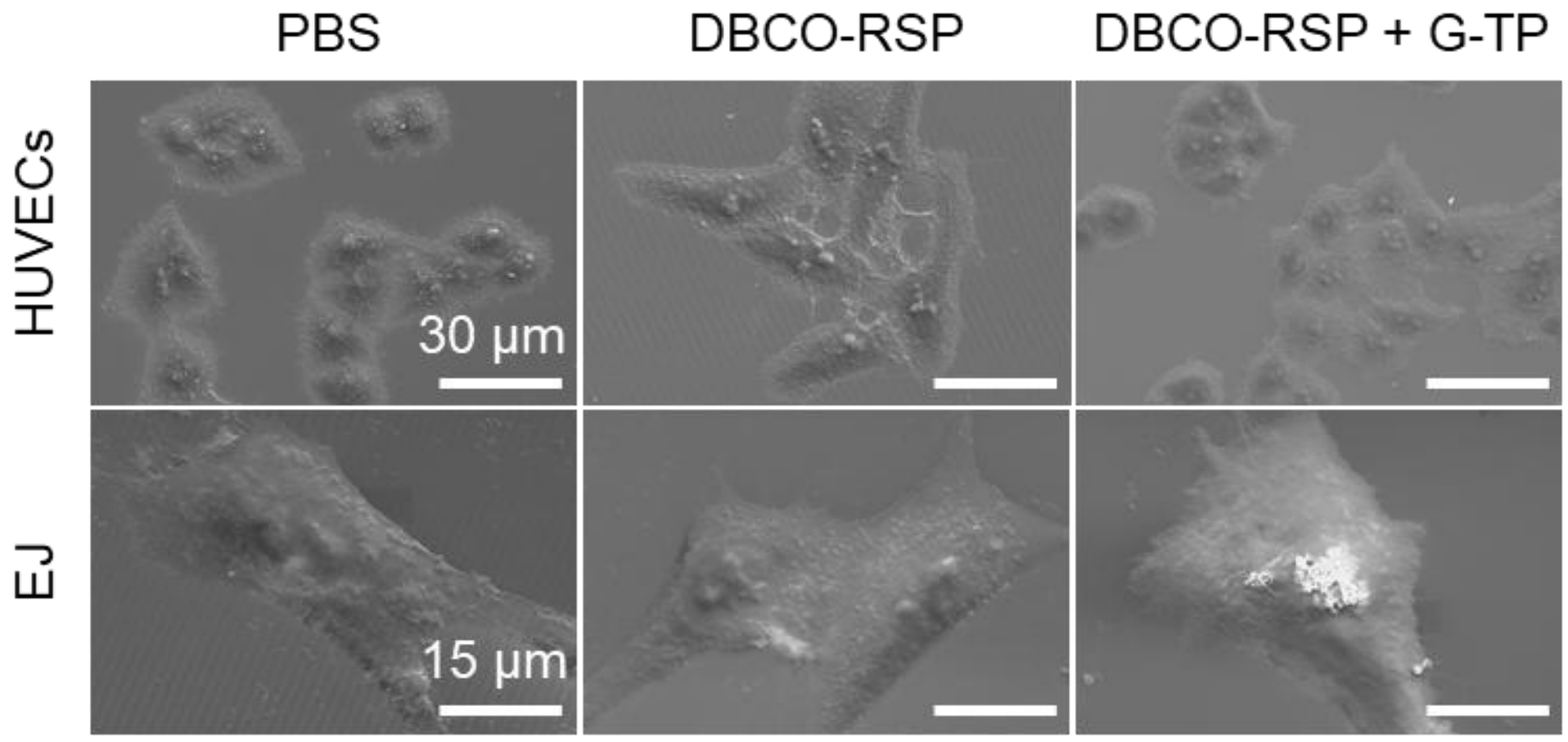

Fig. S42. SEM characterization. SEM images of DBCO-RSP, DBCO-RSP pretreated with G-TP incubated in HUVECs and EJ cells, respectively. The image corresponding to the EJ group in this figure is reused from Fig. 4P.

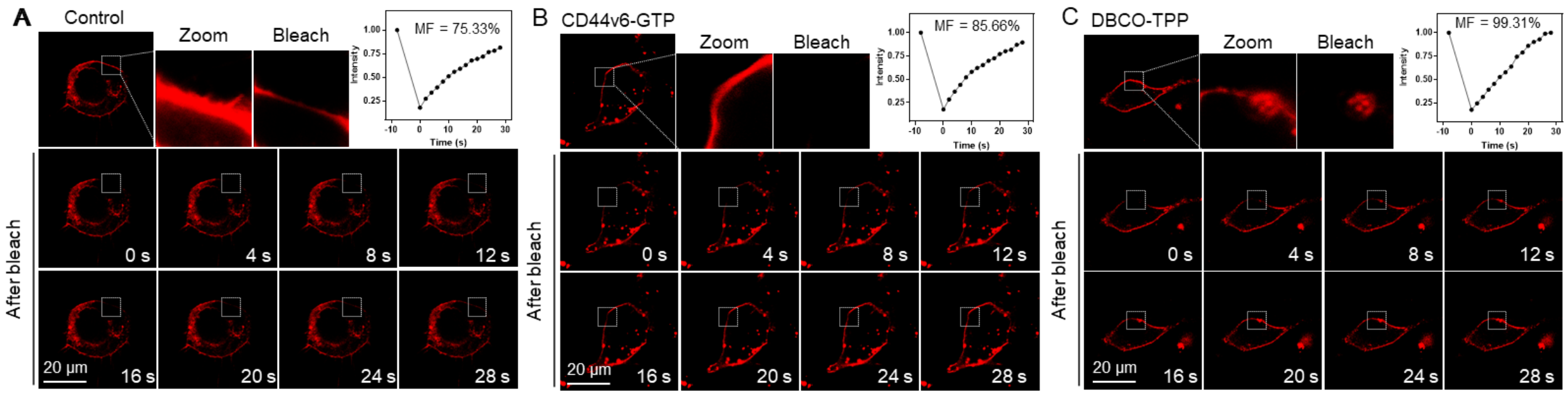

Fig. S43. Evaluating membrane fluidity using fluorescence recovery after photobleaching (FRAP). The results showed that, compared with the control group, cells treated with CD44v6-GTP exhibited an increased fluorescence recovery (85.66%), while an even higher recovery was observed in the DBCO-TPP group (99.31%).

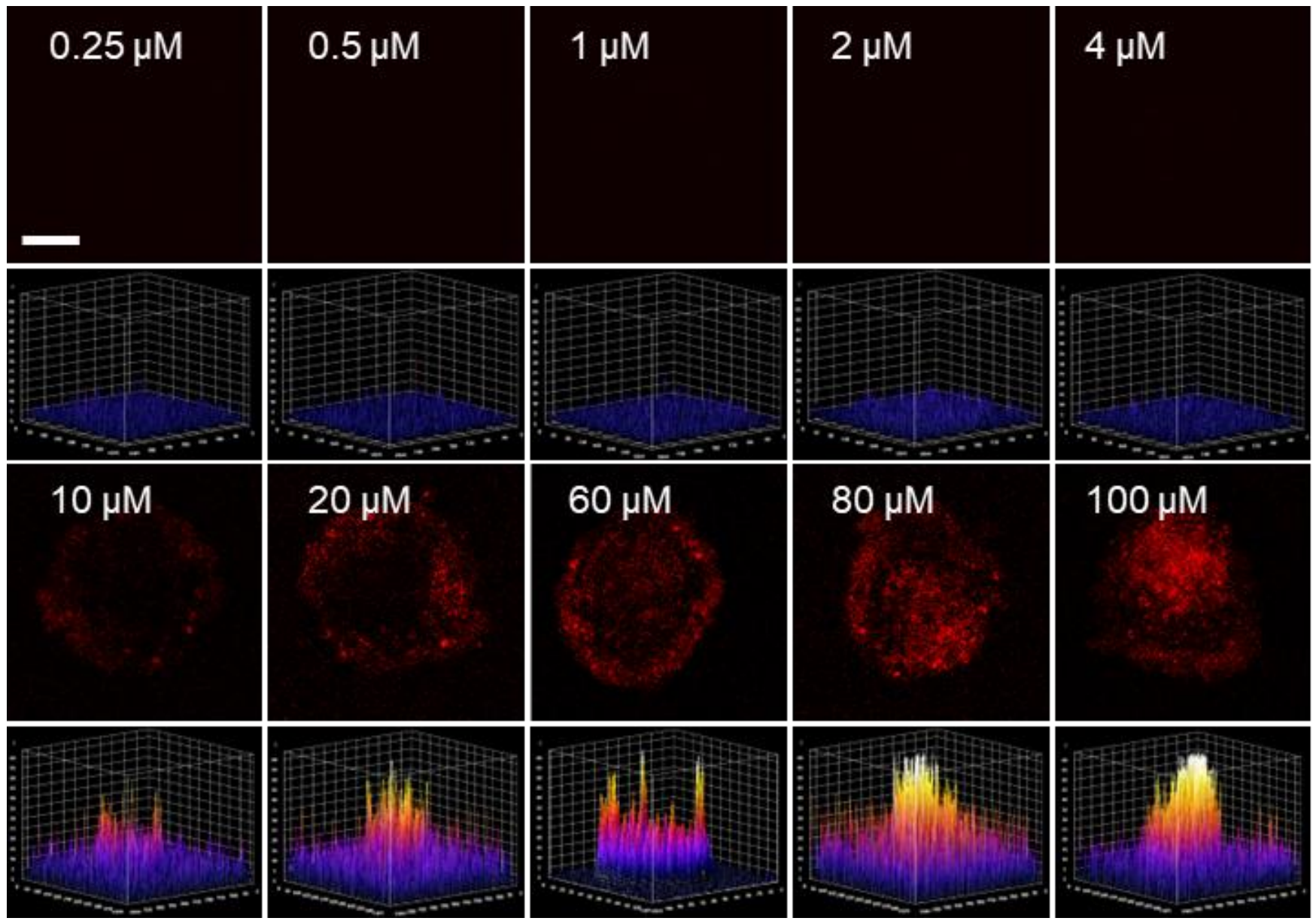

Fig. S44. Validation of BCAR on the EJ MCTs. CLSM images of EJ MCTs incubated with TP-Cy5 at a range of concentrations (0.25 - 100 µM) in the z-axis distance (60 µm). Scale bar: 100 µm.

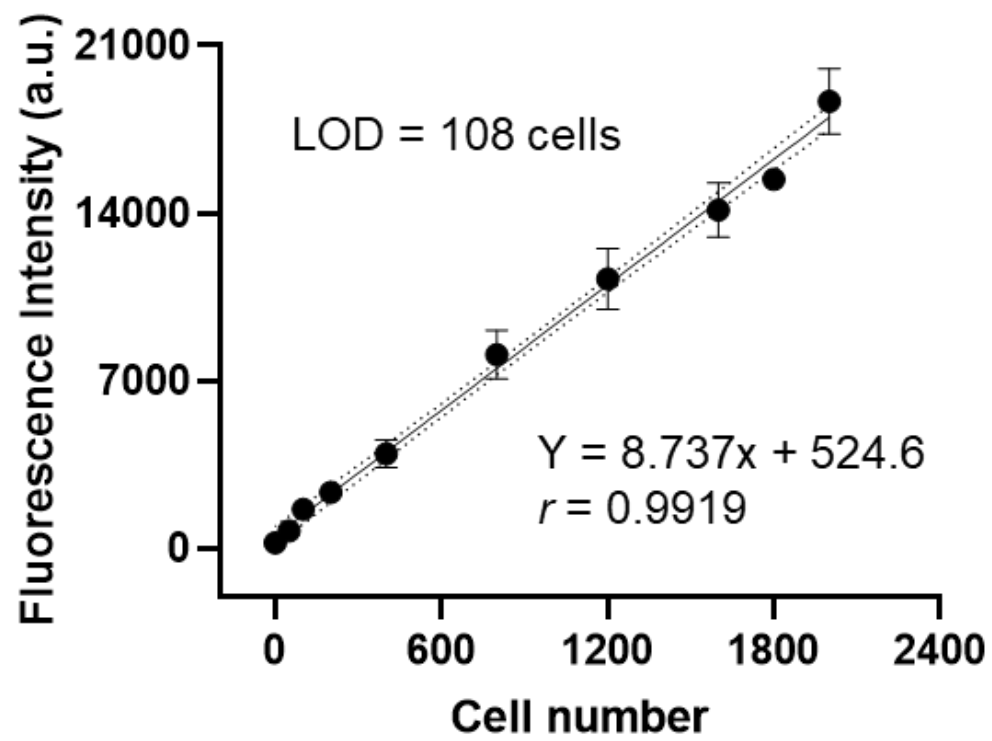

Fig. S45. Determination of detection limit (LOD) in EJ cells using DBCO-TPP and azide-Cy5.

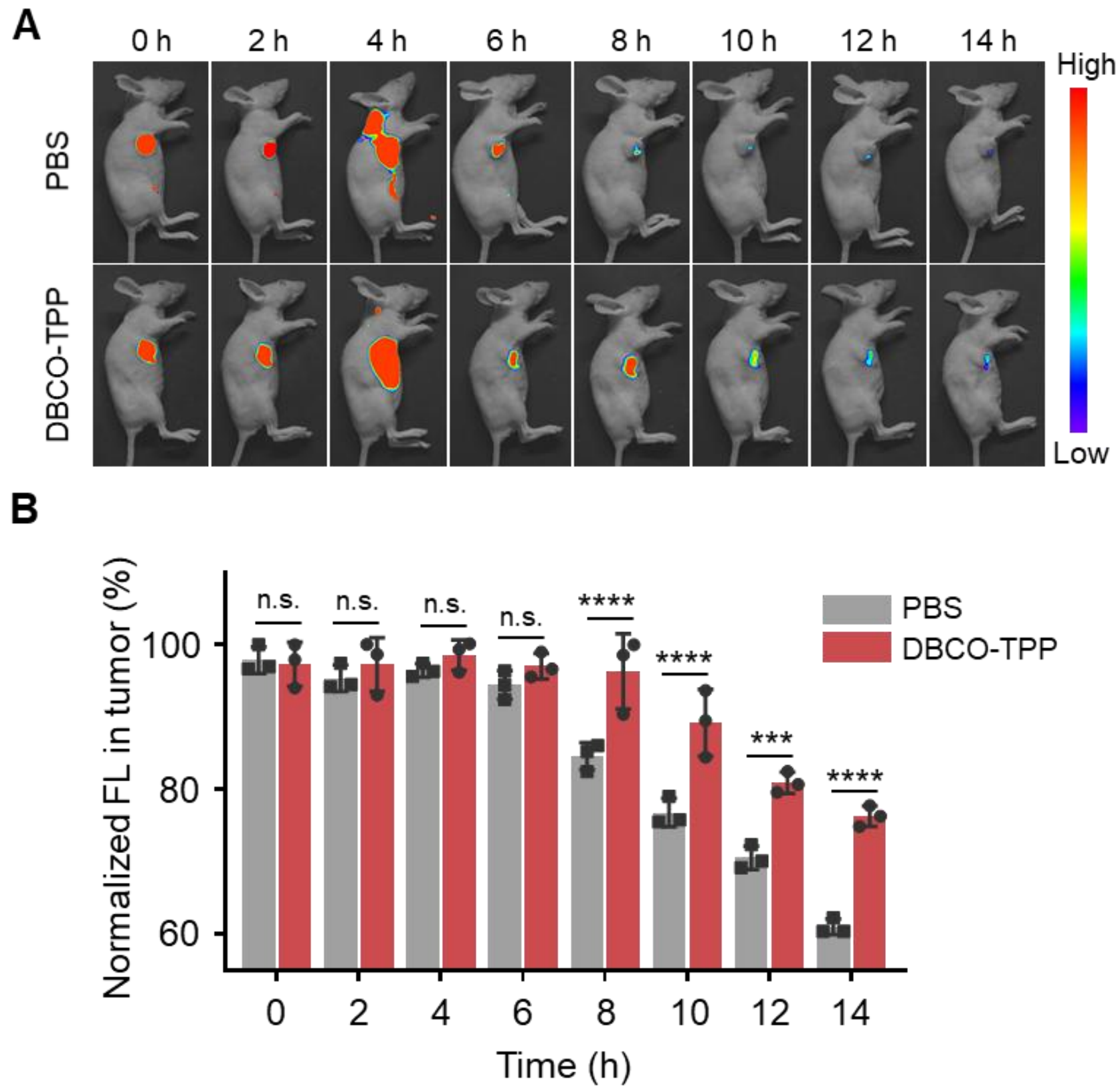

Fig. S46. Evaluation of the retention effect. (A) Fluorescence imaging and **(B)** normalized fluorescence intensity in xenograft model after intratumoral injection of DBCO-TPP (100 µM, 50 µL) and azide-Cy5 (10 µM, 50 µL), as well as PBS and azide-Cy5 (10 µM, 50 µL). The image corresponding to the PBS and DBCO-TPP group in this figure is reused from Fig. 6A.

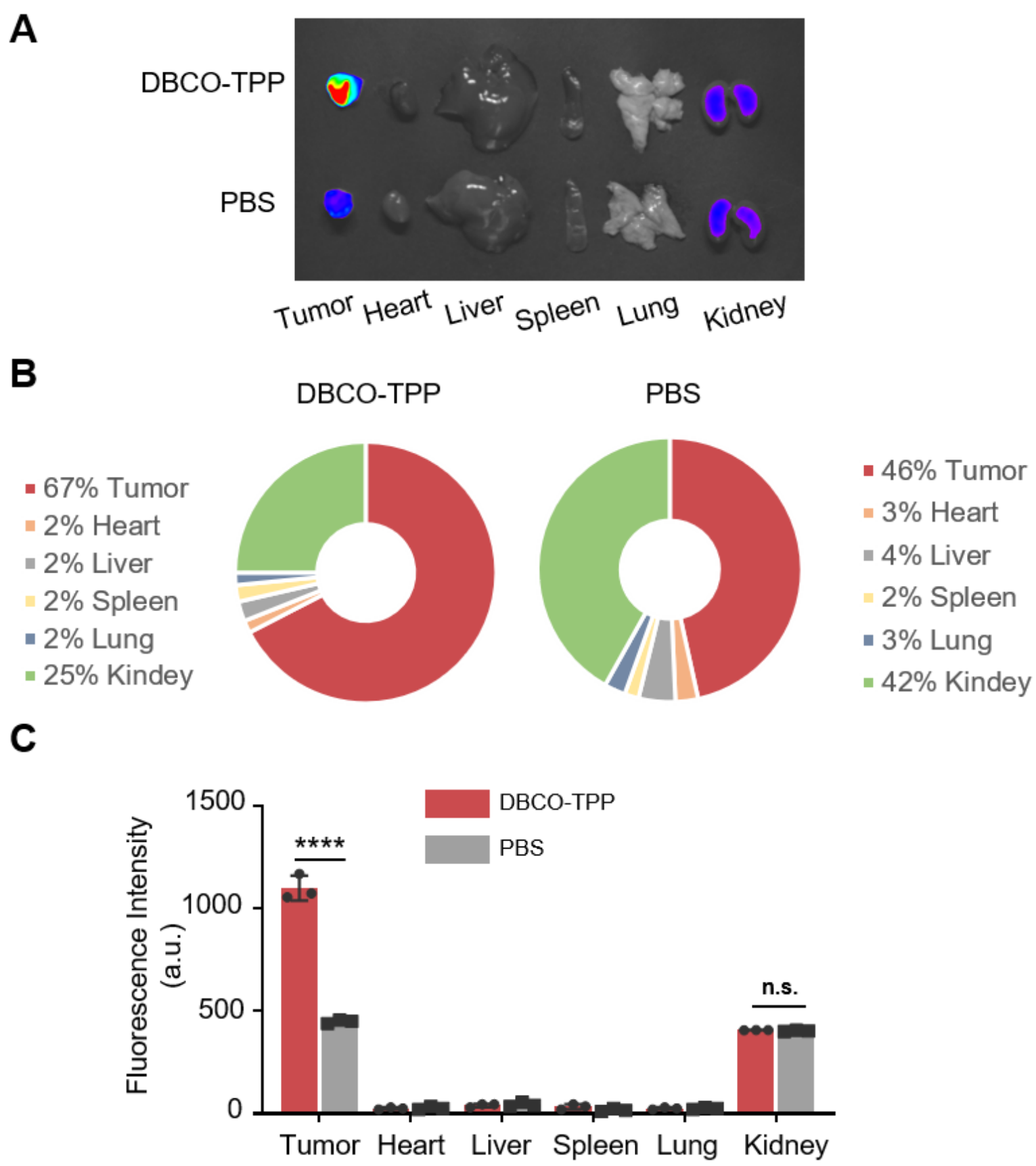

Fig. S47. Fluorescence imaging of isolated organs. (A) Ex vivo imaging, **(B)** distribution, and **(C)** quantitative fluorescence intensity of tumors and major organs 10 hours after intratumoural injection DBCO-TPP (100 μM) + azide-Cy5 (10 μM) and PBS + azide-Cy5 (10 μM). The image corresponding to the PBS and DBCO-TPP group in this figure is reused from Fig. 6C.

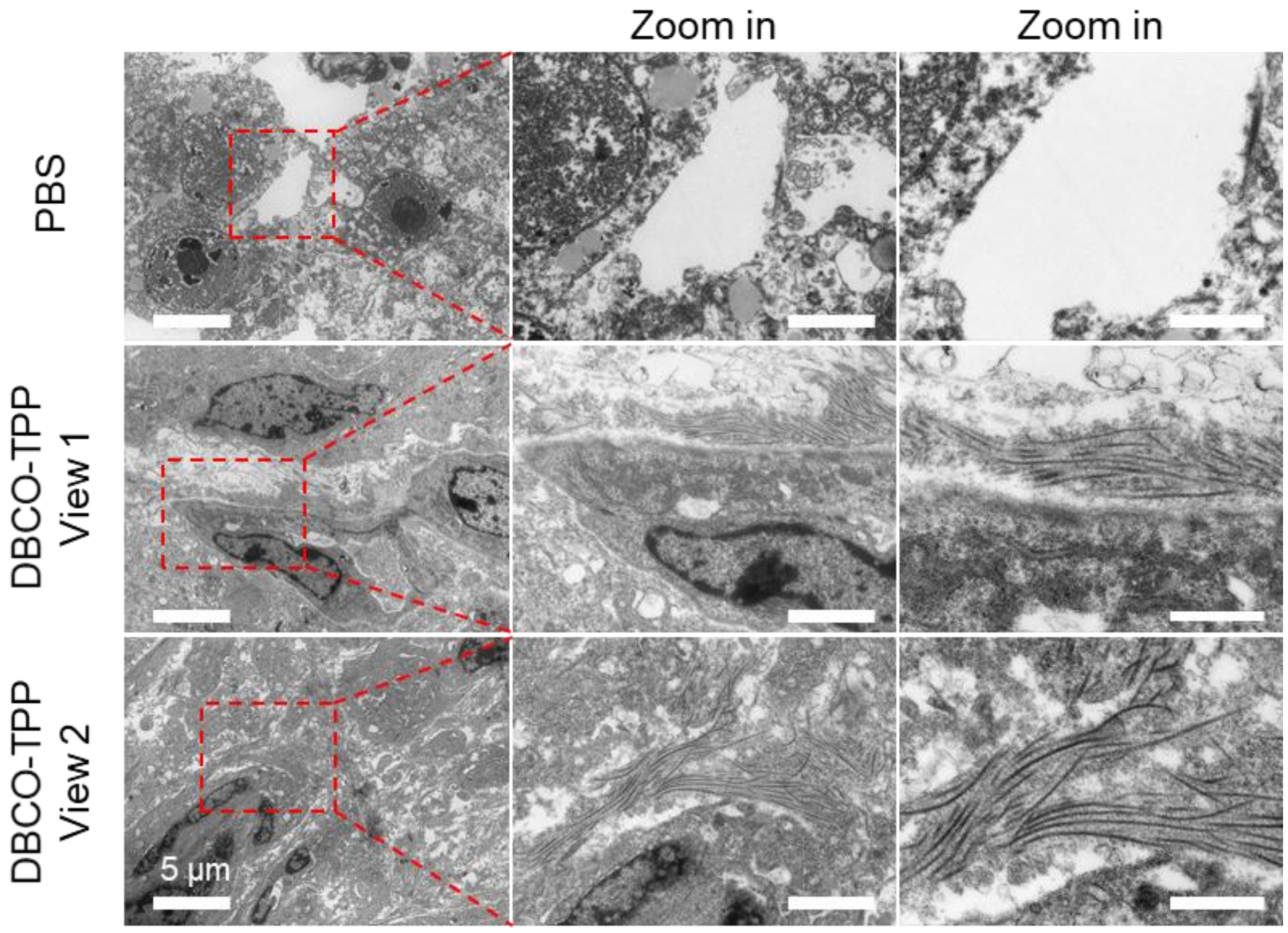

Fig. S48. Bio-TEM characterization. The Bio-TEM images of EJ tumor tissues after treatment with DBCO-TPP (100 μM). The image corresponding to the PBS group in this figure is reused from Fig. 6E.

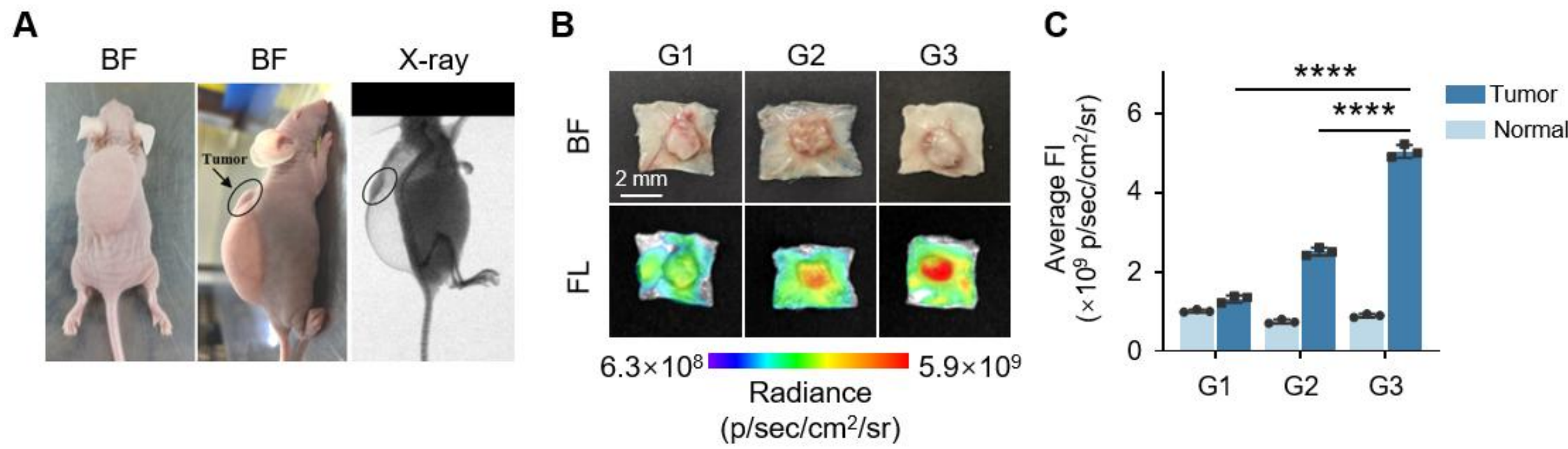

Fig. S49. Establishment of animal models, as well as evaluation of fluorescence imaging performance and biocompatibility. (A) Brighten field and X-ray images of APBC model. **(B)** Bright field and fluorescence imaging of the APBC model treated with G1 (azide-Cy5), G2 (TP-Cy5, 10 μM) and G3 (DBCO-TPP, 50 μM + azide-Cy5, 10 μM). **(C)** Quantitative fluorescence signal in bladder cancer tumor tissue and normal tissue. Data (n = 3) are presented as mean ± SD.

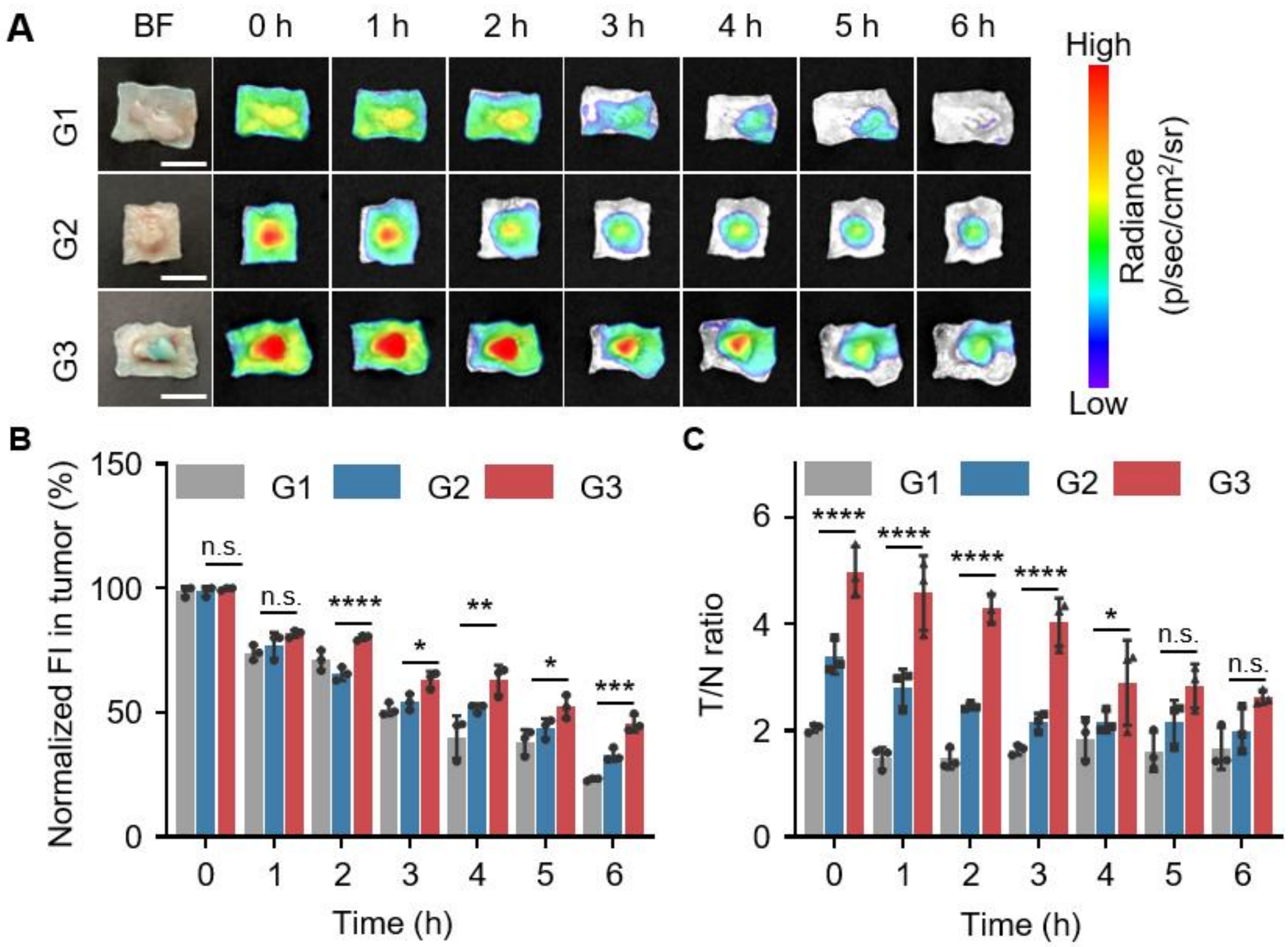

Fig. S50. Evaluation of the photostability. Photostability study of an APBC model. **(A)** Fluorescence imaging, **(B)** corresponding normalized FI in tumor and **(C)** the T/N ratio of APBC imaged with G1 (azide-Cy5, 10 μM), G2 (TP-Cy5, 10 μM), and G3 (DBCO-TPP, 50 μM + azide-Cy5, 10 μM) and then irradiated with 660 nm laser (560 W cm^{-2}) for 0 minute, 1, 2, 3, 4, 5 and 6 hours. Scale bar = 2 mm.

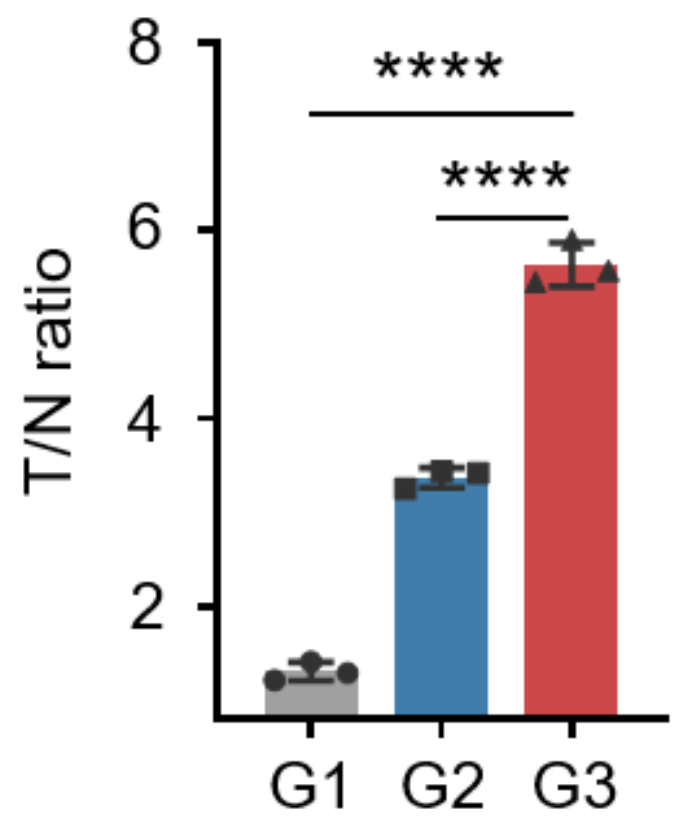

Fig. S51. The T/N ratio of APBC model. The T/N ratio of APBC model treated with G1 (azide-Cy5), G2 (TP-Cy5, 10 μM) and G3 (DBCO-TPP, 50 μM + azide-Cy5, 10 μM). The data are presented as mean ± SD.

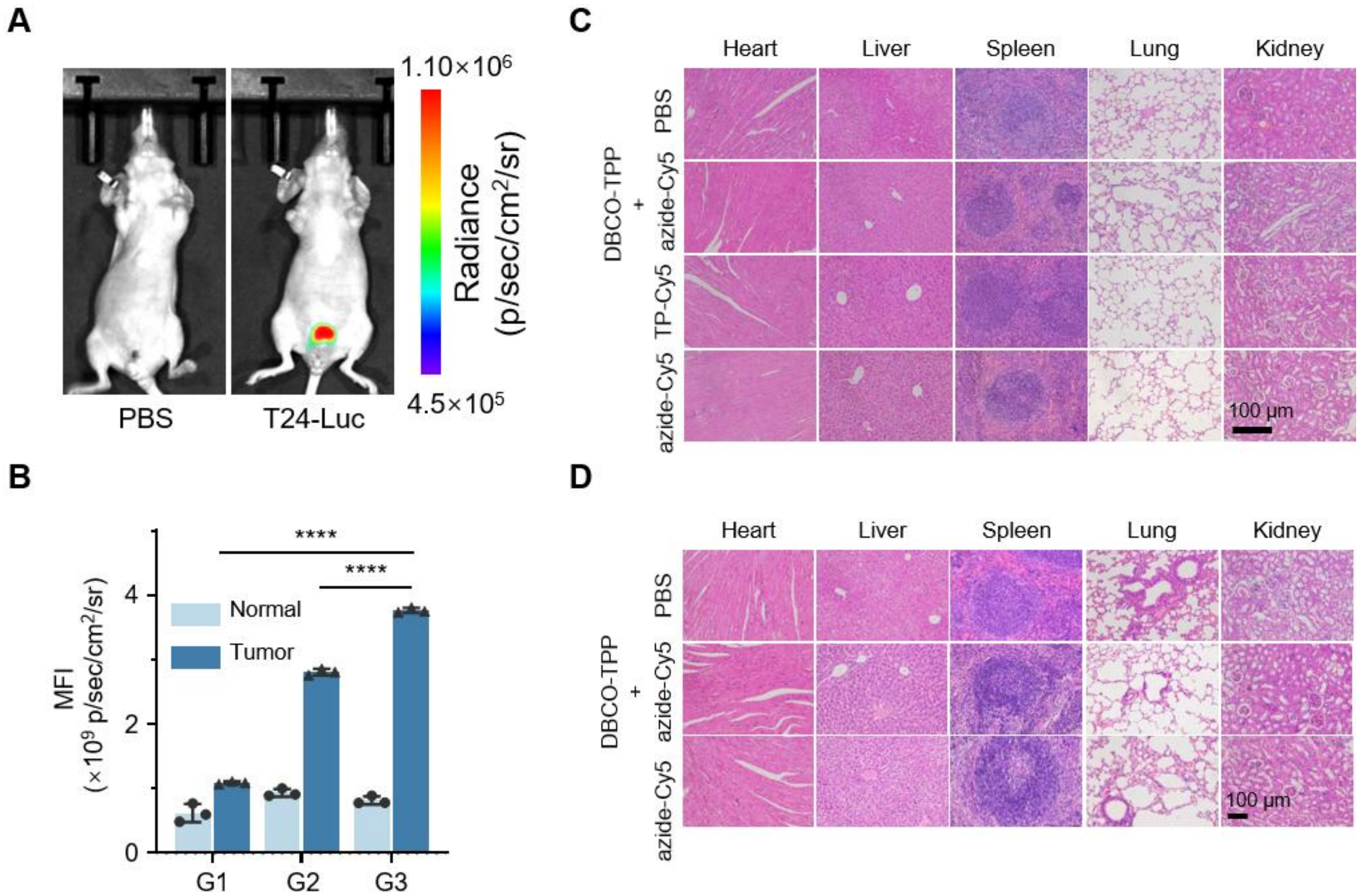

Fig. S52. The bioluminescence image, mean FI and Hematoxylin and eosin (H&E) staining of bladder cancer. (A) Brighten field and bioluminescence image of orthotopic bladder cancer. **(B)** The mean FI of orthotopic bladder cancer model treated with G1 (azide-Cy5, 10 μM), G2 (TP-Cy5, 10 μM), and G3 (DBCO-TPP, 50 μM + azide-Cy5, 10 μM). The data are presented as mean ± SD. **(C)** H&E staining of major organs (heart, liver, spleen, lung and kidney) of orthotopic bladder cancer mice treated with PBS, DBCO-TPP (50 μM) + azide-Cy5 (10 μM), TP-Cy5 (10 μM) and azide-Cy5 (10 μM). **(D)** H&E staining of major organs (heart, liver, spleen, lung and kidney) of air-pouch bladder cancer mice treated with PBS, DBCO-TPP (50 μM) + azide-Cy5 (10 μM), and azide-Cy5 (10 μM).

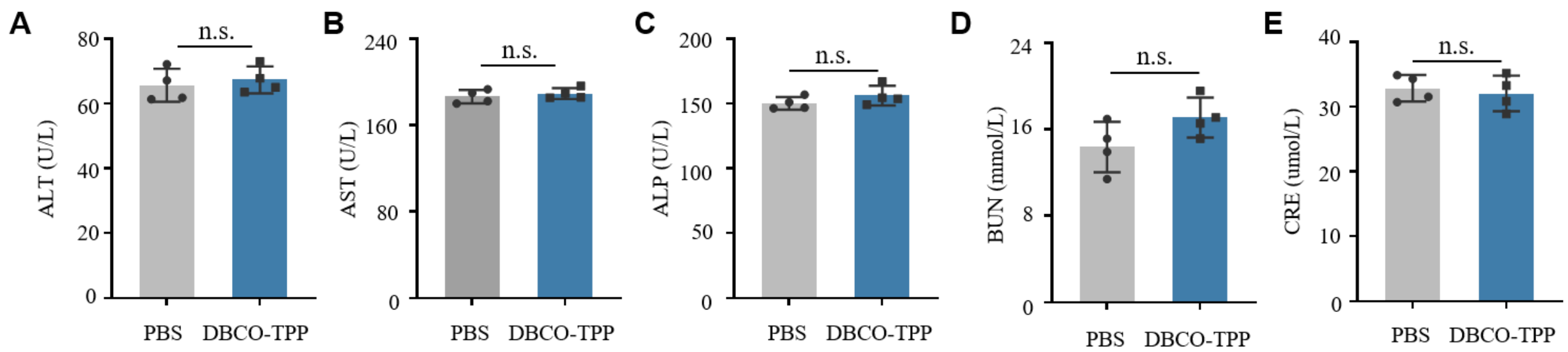

Fig. S53. Evaluation of liver and kidney function. The indicators of liver and kidney function were analyzed in healthy female BALB/c nude mice treated with DBCO-TPP (50 μM), including alanine aminotransferase (ALT), aspartate aminotransferase (AST), alkaline phosphatase (ALP), blood urea nitrogen (BUN) and creatinine (CRE).

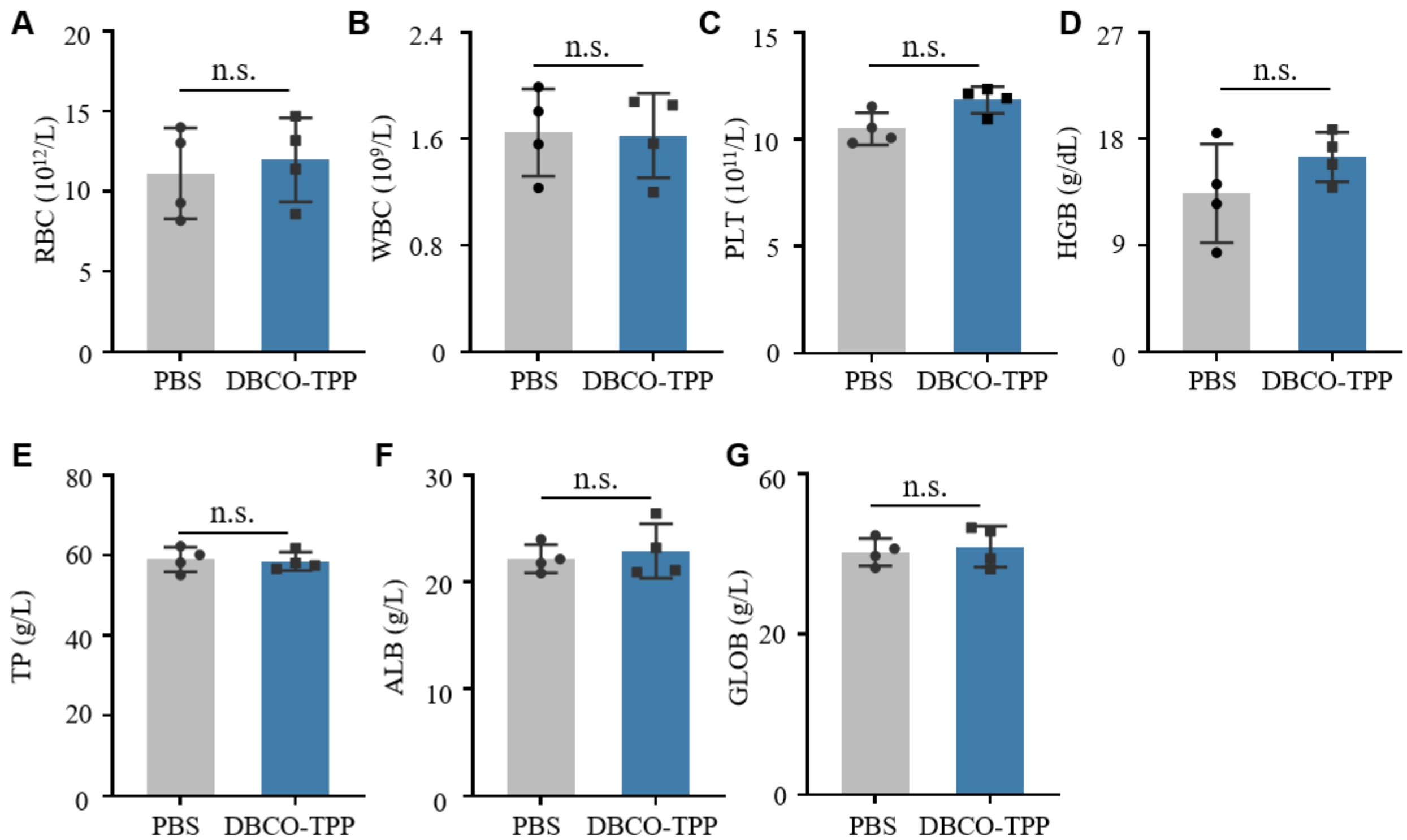

Fig. S54. Evaluation of blood routine and protein biomarkers. The indicators of blood routine and protein biomarkers were examined in healthy female BALB/c nude mice treated with DBCO-TPP (50 μM), including red blood cell (RBC), white blood cell (WBC), platelets (PLT), and hemoglobin (HGB). Other indicators include total protein (TP), albumin (ALB) and globulin (GLOB).

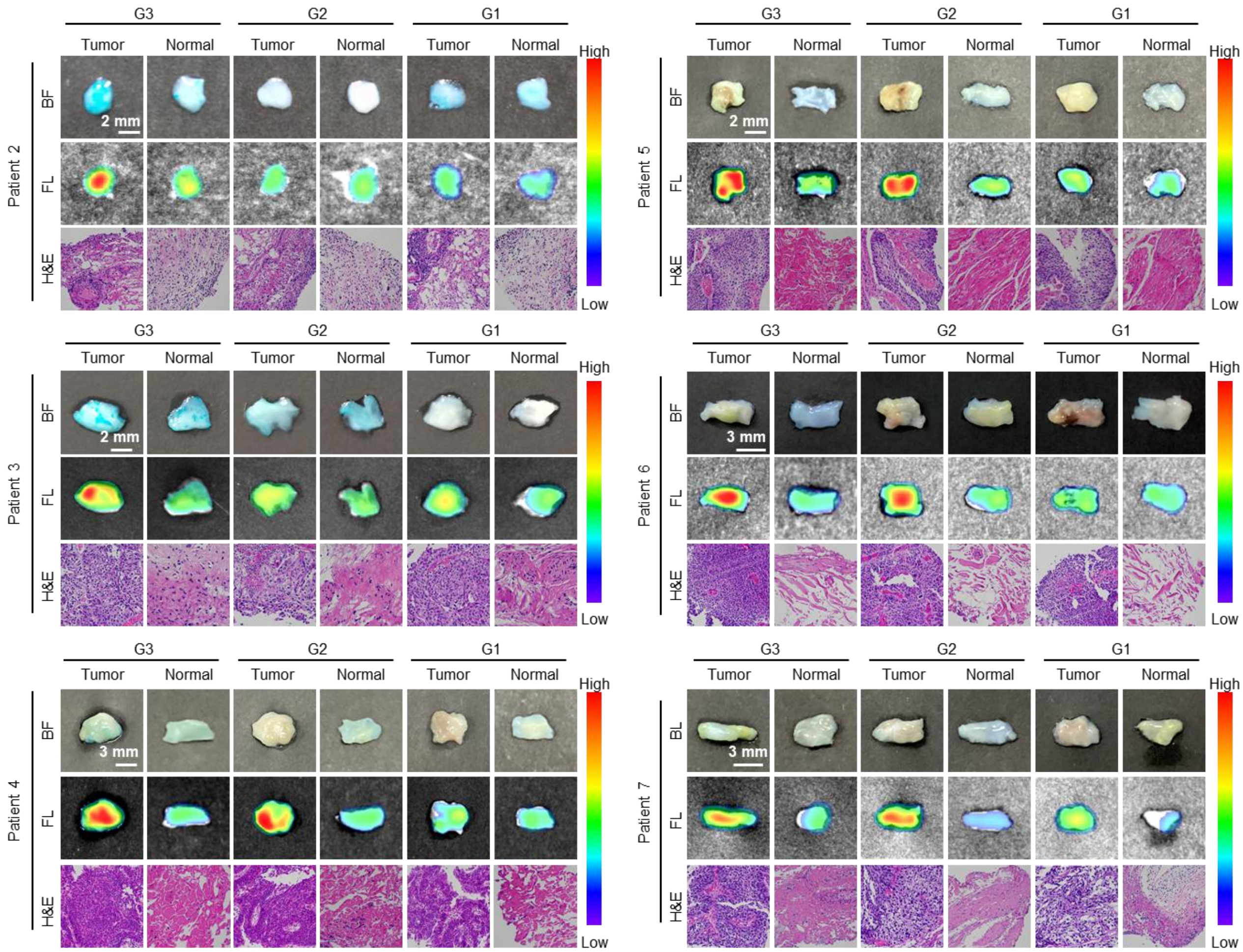

Fig. S55. Fluorescence imaging of tumor and normal tissues in bladder cancer patients. The fluorescence signal imaging of tumor tissues and normal bladder cancer tissues from patient 2 to 7 after incubation with G1 (azide-Cy5), G2 (TP-Cy5), and G3 (DBCO-TPP + azide-Cy5).

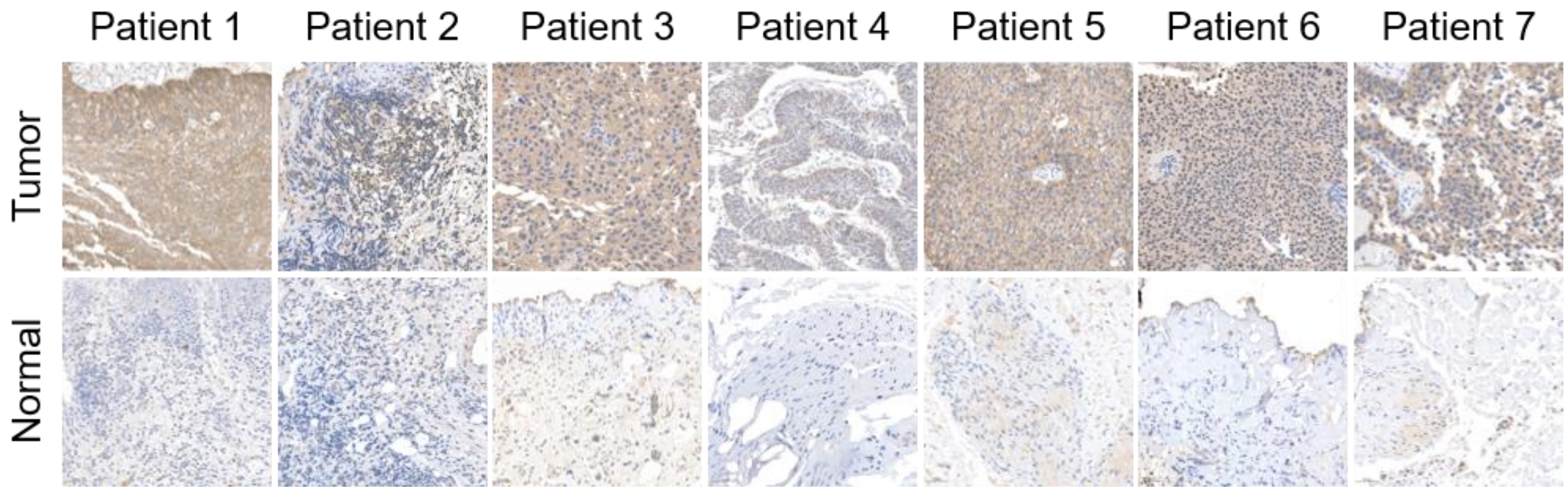

Fig. S56. Expression of CD44v6 by IHC. The expression of CD44v6 in isolated tumor tissues and normal tissues was verified by IHC assay. Original magnification 20×.

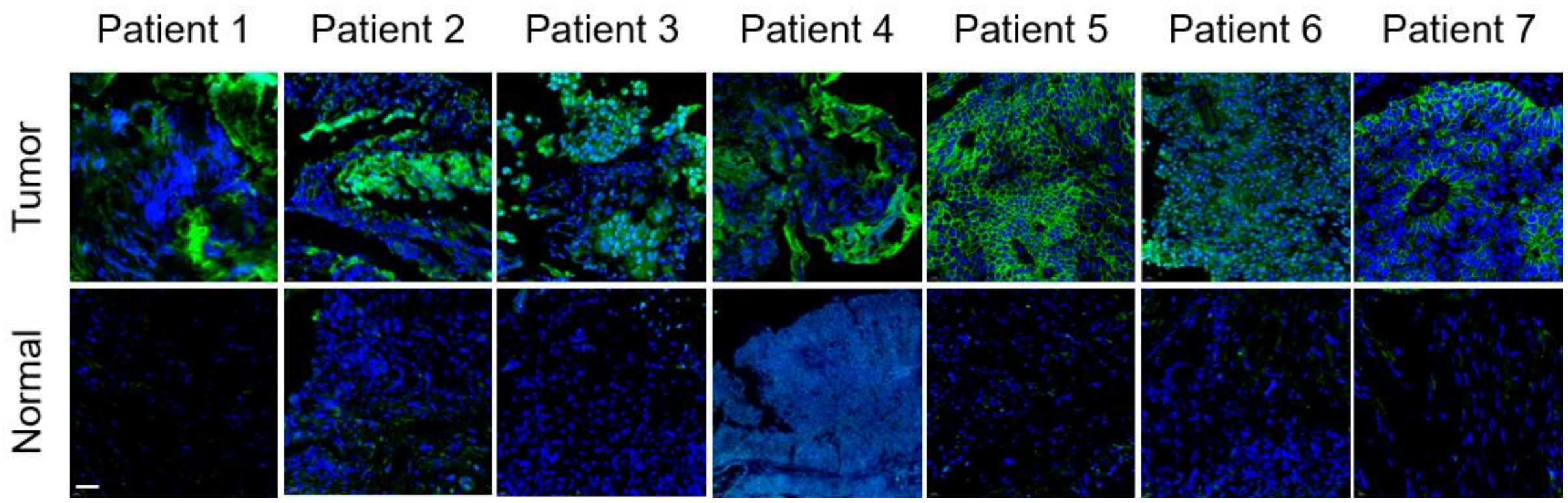

Fig. S57. Expression of CD44v6 by IF. The expression of CD44v6 in isolated tumor tissues and normal tissues was verified by IF assay. Scale bar = 20 μm.

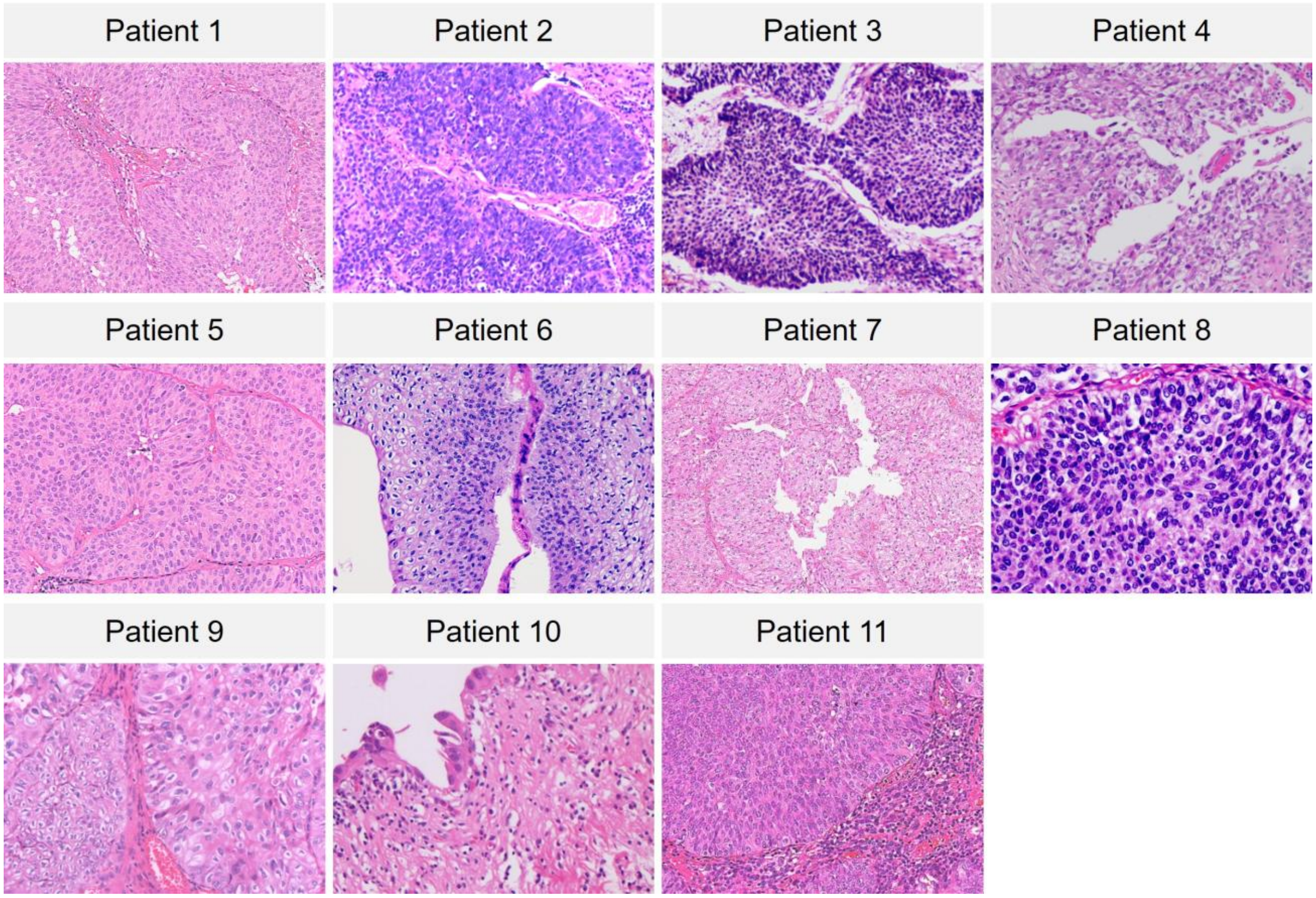

Fig. S58. HE staining of tumor tissue in RNA-seq analysis. Pathological report of HE staining of tumor tissue from NMIBC patients in RNA-seq analysis.

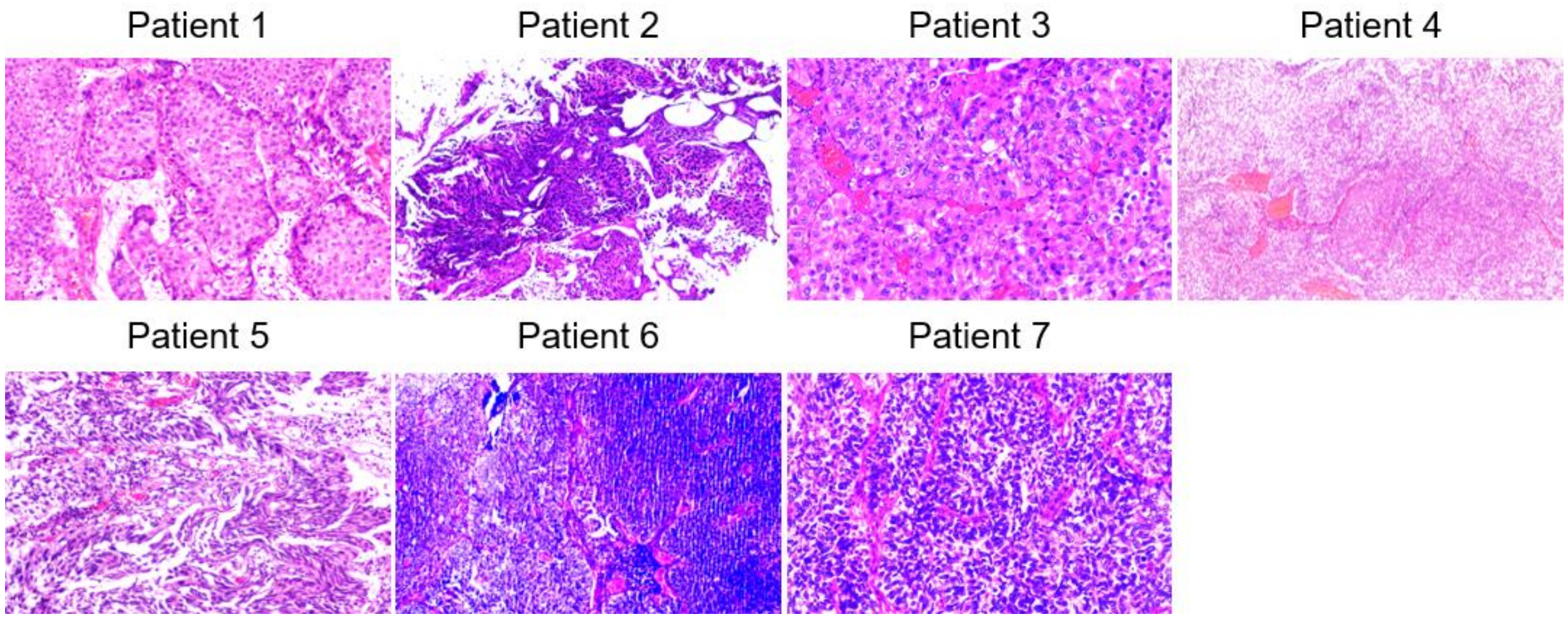

Fig. S59. HE staining of tumor tissue in ex vivo imaging. Pathological report of HE staining of tumor tissue from NMIBC patients of ex vivo imaging in human tumor tissue.

Supplementary Tables

Table S1. The contact area (A^2) of DBCO-RSP with and without G-TP interacting with a lipid bilayer at 45 ns.

**Terms**	**Contact area (A^2)**
DBCO-RSP	0
DBCO-RSP and G-TP	196.8

Table S2. Summary of the ex vivo imaging study of human clinical tissue.

Type	Sum (n)	True positive (n)	True negative (n)	False positive (n)	False negative (n)	Sensitivity (%)	Specificity (%)
clinical tissue	22	9	10	1	2	81.8	90.9

Table S3. Clinicopathological characteristics of NMIBC patients in RNA-seq experiment. Sex: M = male, F = female; Tissue sample is obtained from the patient after TURBT. T = Tumor, N = Normal; Primary/Recurrence: P = Primary, R = Recurrence; Grade: H = High, L = Low; TURBT: Transurethral resection of bladder tumors; LRC: Laparoscopic radical cystectomy;

No.	Sex	Age	Tissue	Primary/ Recurrence	TNM	Grade	Treatment
1	M	68	T	R	T1N0M0	L	TURBT
2	F	70	T	P	T1N0M0	L	TURBT
3	M	61	T	P	T1N0M0	H	TURBT
4	M	81	T	P	T1N0M0	H	TURBT
5	M	71	T	P	T1N0M0	L	TURBT
6	M	77	T	P	T1N0M0	H	TURBT
7	M	71	T	P	T1N0M0	L	TURBT
8	M	50	T	P	T1N0M0	H	TURBT + Gemcitabine + Cisplatin
9	F	59	T	P	T1N0M0	H	TURBT + Gemcitabine + Cisplatin
10	F	75	T	P	T1N0M0	L	TURBT
11	M	87	T	P	T1N0M0	L	TURBT
12	M	78	N	P	T1N0M0	H	TURBT
13	M	50	N	R	T1N0M0	L	TURBT + Gemcitabine + Cisplatin
14	M	77	N	R	T1N0M0	H	LRC
15	M	60	N	R	T1N0M0	L	TURBT
16	M	71	N	P	T1N0M0	L	TURBT
17	M	59	N	P	T1N0M0	L	TURBT
18	M	62	N	P	T1N0M0	L	TURBT + Gemcitabine + Cisplatin
19	M	69	N	P	T1N0M0	L	TURBT
20	M	68	N	P	T1N0M0	L	TURBT
21	M	71	N	P	T1N0M0	L	TURBT
22	M	50	N	P	T1N0M0	L	TURBT

Table S4. Clinicopathological characteristics of NMIBC patients in ex vivo imaging in human tumor tissue.

No.	Sex	Age	Tissue	Primary/ Recurrence	TNM	Grade	Treatment
1	M	74	T	P	T1N0M0	H	TURBT
2	F	79	T	P	T1N0M0	L	TURBT
3	M	66	T	P	T1N0M0	H	TURBT
4	M	70	T	P	T1N0M0	L	TURBT
5	M	75	T	P	T1N0M0	H	Toripalimab + Disitamab Vedotin
6	M	73	T	P	T1N0M0	H	TURBT
7	M	87	T	P	T1N0M0	H	TURBT
